# Supplementary material for: Identification of Potential Host Plants of Sap-Sucking Insects (Hemiptera: Cicadellidae) Using Anchored Hybrid By-Catch Data
Source: Insects. 2021 Oct 23;12(11):964. doi: 10.3390/insects12110964 (PMC8617646; doi:10.3390/insects12110964)
Supplement: Supplementary file 1 [file insects-12-00964-s001.zip › Supplementary file 1.pdf]

Supplementary file 1: retrieved plant sequences.

>P016_WG08_k90.1090 [Draeculacephala minerva, 16S (partial)]
AACTCTGTGCCAGCAGCCGCGGTAAGACAGAGGATGCAAGCGTTATCCGGAATGAT
TGGGCGTAAAGCGTCTGTAGGTGGCTTTTCAAGTCCGCCGTCAAATCCCAGGGCTCA
ACCCTGGACAGGCGGTGGAAACTACCAAGCTGGAGTACGGTAGGGGCAGAGGGAA
TTTCCGGTGGAGCGGTGAAATGCGTTGAGATCGGAAAGAACACCAACGGCGAAAGC
ACTCTGCTGGGCCGACACTGACACTGAGAGACGAAAGCTAGGGGAGCAAATGGGAT
TAGATACCCCAGTAGTCCTAGCCGTAAACGATGGATACTAAGTGCTGTGCGTATCGA
CCCGCGCAGTGCTGTAGCTAACGCGTTAAGTATCCCGCCTGGGGAGTACGTTCGCAA
GAATGAAACTCAAAGGAATTGACGGGGGCCCGCACAAGCGGTGGAGCATGTGGTTC
AATTCGATGCTAAGCGAAGAACCTTACCAGGGCTTGACATGCCGTGAATCCTCCCGA
AAGAGAGGAGTGCCTTCGGGAACGCGGACACAGGTGGTGCATGGCTGTCGTCAGCT
CGTGCCGTAAGGTGTTGGGTTAAGTCCCGCAACGAGCGCAACCCTCGTGTTTAGTTG
CCAGCATTGAGTTTGGAACCCTGAACAGACTGCCGGTGATAAGCCGGAGGAAGGTG
AGGATGACGTCAAGTCATCATGCCCCTTACGCCCTGGGCGACACACGTGCTACAATG
GCCGGGACAAAGGGTCGCGACCCCGCGAGGGCAAGCTAACCTCAAAAACCCGGCCT
CAGTTCGGATTGCAGGCT
>P019_WA10_k29.20710 [Emadiana cuprea, matK (partial)]
ATTCCCCAATCTTCTACCTTCATTTCAAATATACTATTAAATGGAGGAAATCCAAAG
CTATTTACAACTTGATAGATTTCAACAGCCCGGCTTTCTATATCCACTTATCTTTCAG
GAGTATATTTACGGGCTTGTTCATGATTTTAGTTTAAATCGATC
>P019_WA10_k50.13716 [Emadiana cuprea, rbcL (partial)]
GAGCCGCAGTAGCTGCCGAATCTTCTACTGGTACATGGACAACTGTATGGACCGACG
GACTTACCAGTCTTGATCGTTACAAAGGACGATGCTACCACATCGATGCCGTTCCTG
GAGAAGACAATCAATATATTTGTTATGTAGCTTACCCCTTAGACCTTTTTGAAGAAG
GT
>P019_WG02_k90.493 [Cuerna unica, 16S (partial)]
TCTCATGGAGAGTTCGATCCTGGCTCAGGATGAACGCTGGCGGCATGCTTAACACAT
GCAAGTCGGACGGGAAGTGGTGTTTCCAGTGGCGGACGGGTGAGTAACGCGTAAGA
ACCTGCCCTTGGGAGGGGAACAACAGCTGGAAACGGCTGCTAATACCCCGTAGGCT
GAGGAGCAAAAGGAGGAATCCGCCCGAGGAGGGGCTCGCGTCTGATTAGCTAGTTG
GTGAGGCAATAGCTTACCAAGGCGATGATCAGTAGCTGGTCCGAGAGGATGATCAG
CCACACTGGGACTGAGACACGGCCCAGACTCCTACGGGAGGCAGCAGTGGGGAATT
TTCCGCAATGGGCGAAAGCCTGACGGAGCAATGCCGCGTGGAGGTAGAAGGCCTAC
GGGTCGTGAACTTCTTTTCCCGGAGAAGAAGCAATGACGGTATCTGGGGAATAAGC
ATCGGCTAACTCTGTGCCAGCAGCCGCGGTAATACAGAGGATGCAAGCGTTATCCG
GAATGATTGGGCGTAAAGCGTCTGTAGGTGGCTTTTTAAGTCCGCCGTCAAATCCCA
GGGCTCAACCCTGGACAGGCGGTGGAAACTACCAAGCTGGAGTACGGTAGGGGCAG
AGGGAATTTCCGGTGGAGCGGTGAAATGCGTAGAGATCGGAAAGAACACCAACGGC
GAAAGCACTCTGCTGGGCCGACACTGACACTGAGAGACGAAAGCTAGGGGAGCAA
ATGGGATTAGATACCCCAGTAGTCCTAGCCGTAAACGATGGATACTAGGCGCTGTGC
GTATCGACCCGTGCAGTGCTGTAGCTAACGCGTTAAGTATCCCGCCTGGGGAGTACG
TTCGCAAGAATGAAACTCAAAGGAATTGACGGGGGCCCGCACAAGCGGTGGAGCAT
GTGGTTTAATTCGATGCAAAGCGAAGAACCTTACCAGGGCTTGACATGCCGCGAATC
CTCTTGAAAGAGAGGGGTGCCTTCGGGAACGCGGACACAGGTGGTGCATGGCTGTC

GTCAGCTCGTGCCGTAAGGTGTTGGGTTAAGTCCCGCAACGAGCGCAACCCTCGTGT
TTAGTTGCCACCGTTGAGTTTGGAACCCTGAGCAGACTGCCGGTGATAAGCCGGAGG
AAGGTGAGGATGACGTCAAGTCATCATGCCCCTTATGCCCTGGGCGACACACGTGCT
ACAATGGCCGGGACAAAGGGTCGCGATCCCGCGAGGGTGAGCTAACTCCAAAAACC
CGTCCTCAGTTCGGATTGTAGGCTGCAACTCGCCTACATGAAGCCGGAATCGCTAGT
AATCGCCGGTCAGCCATACGGCGGTG
>P019_WG02_k29.49188 [Cuerna unica, rbcL (partial)]
AATGTATTTGGGTTCAAAGCCCTGCGTGCTCTACGTTTGGAGGATTTGCGAATCCCT
GTTGCTTATATAAAAACTTTCCAAGGCCCGCCTCACGGTATCCAAGTTGAGAGAGAT
AAATTGAACAAGTATGGCCGTCCTCTACTGGGATGCACTATTAAGCCGAAATTGGGG
TTATCTGCTAAAAACTATGGTCGAGCGGTTTATGAATGTCTTCGCGGTGGACTTGAT
TTTACCAAAGATGACGAAAACGTGAACTCCCAACCATTTATGCGTTGGAGAGACCGT
TTCTTATTTTGTGCCGAAGCAATTTATAAAGCACAGGCCGAAACAGGTGAAATCAAA
GG
>P019_WE06_k50.2203 [Coelidia sp., rbcL (partial)]
GTTTACTTCCATTGTCGGTAATGTATTTGGGTTCAAAGCCCTGCGTGCTCTACGTTTG
GAGGATTTGCGAATCCCTGTTGCTTATATAAAAACTTTCCAAGGCCCGCCTCACGGT
ATCCAAGTTGAGAGAGATAAATTGAACAAGTATGGCCGTCCTCTACTGGGATGCACT
A
>P019_WE01_k50.7628 [Collasuyusana retrorsa, rbcL (partial)]
GTTCTGTTACTAATATGTTTACTTCCATTGTGGGTAATGTATTTGGGTTCAAAGCCCT
GCGTGCTCTACGTTTGGAGGATTTGCGAATCCCTGTTGCTTATATAAAAACTTTCCAA
GGCCCGCCTCACGGTATCCAAGTTGAGAGAGATAAATTGAACAAGTATGGCCGTCC
TCTACTGGGATGCACTATTAAGCCGAAATTGGGGTTATCTGCTAAAAACTATGGTCG
AGCGGTTTATGAATGTCTTCGCGGTGGACTTGATTTTACCAAAGATGACGAAAACGT
GAACTCCCAACCATTTATGCGTTGGAGAGACCGTTTCTTATTTTGTGCCGAAGCAAT
TTATAAAGCACAGG
>P019_WH08_k29.12093 [Deltolidia discolor, matK (partial)]
TACTTTGTTTTTGACTGTATCGCACTGTGTATCATTGGAAAATTTCCCACTCTTCTAC
CTTCATTTCAAATATACTATTAAATGGAGGAAATCCAAAGCTATTTACAACTTGATA
GATCTCAACAGCCCGGCTTTCTATATCCACTTATTTTTCAGGAGTATGTTTACGGGCT
TGCT
>P019_WE02_k29.46238 [Dialodia pectinata, rbcL (partial)]
AGGTTCTGTTACTAATATGTTTACTTCCATCGTGGGCAATGTATTTGGGTTCAAAGCC
CTGCGTGCTCTACGTTTGGAGGATTTGCGAATCCCTGTTGCTTATATAAAAACTTTCC
AAGGCCCGCCTCACGGTATCCAAGTTGAGAGAGATAAATTGAACAAGTATGGCCGT
CCTCTACTGGGATGCACTATTAAGCCGAAATTGGGGTTATCTGCTAAAAACTAT
>P019_WA02_k29.181165 [Coelidiini New Genus n. sp., rbcL (partial)]
AATGTATTTGGGTTCAAAGCCCTGCGTGCTCTACGTTTGGAGGATTTGCGAATCCCT
GTTGCTTATATAAAAACTTTCCAAGGCCCGCCTCACGGTATCCAAGTTGAGAGAGAT
AAATTGAACAAGTATGGCCGTCCTCTACTGGGATGCACTATTAAGCCGAAATTGGGG
TTATCTGCTAAAAACTATGGTCGAG
>P019_WE04_k29.18236 [Thagria multipars, matK (partial)]
CTTTGGTTCAAATCTAATATCAAATGGAGGAAATCCAAAGATATTTACAGCTTGATA
GATCTCAACAACACGGCTTTCTATATCCACTTATCTTTCAGGAGTATATTTATGCACT

TGCTCATGATCATAGTTTAAACCGATCTATTTTGTTGGAAAATCCAGGTTATGACAA
TCAATTCAGTTTCCTAATTGTGAAACGTTTAATTACTCGAATGTAT
>P019_WE04_k29.29407 [Thagria multipars, rbcL (partial)]
AATGTATTTGGGTTCAAAGCCCTGCGTGCTCTACGTTTGGAGGATTTGCGAATCCCT
GTTGCTTATATAAAAACTTTCCAAGGCCCGCCTCACGGTATCCAAGTTGAGAGAGAT
AAATTGAACAAGTATGGCCGTCCTCTACTGGGATGCACTATTAAGCCGAAATTGGGG
TTATCTGCTAAAAACTATGGTCGAGCGGTTTATGAATGTCTTCGCGGTGGACTT
>P015_WE10_k90.13196 [Acinopterus inornatus, 16S (partial)]
TCTCATGGAGAGTTCGATCCTGGCTCAGGATGAACGCTGGCGGCATGCTTAACACAT
GCAAGTCGGACGGGAAGTGGTGTTTCCAGTGGCGGACGGGTGAGTAACGCGTAAGA
ACCTGCCCTTGGGAGGGGAACAACAGCTGGAAACGGCTGCTAATACCCCGTAGGCT
GAGGAGCAAAAGGAGGAATCCGCCCGAGGAGGGGCTCGCGTCTGATTAGCTAGTTG
GTGAGGCAATAGCTTACCAAGGCGATGATCAGTAGCTGGTCCGAGAGGATGATCAG
CCACACTGGGACTGAGACACGGCCCAGACTCCTACGGGAGGCAGCAGTGGGGAATT
TTCCGCAATGGGCGAAAGCCTGACGGAGCAATGCCGCGTGGAGGTAGAAGGCCCAC
GGGTCGTGAACTTCTTTTCCCGGAGAAGAAGCAATGACGGTATCTGGGGAATAAGC
ATCGGCTAACTCTGTGCCAGCAGCCGCGGTAATACAGAGGATGCAAGCGTTATCCG
GAATGATTGGGCGTAAAGCGTCTGTAGGTGGCTTTTTAAGTCCGCCGTCAAATCCCA
GGGCTCAACCCTGGACAGGCGGTGGAAACTACCAAGCTGGAGTACGGTAGGGGCAG
AGGGAATTTCCGGTGGAGCGGTGAAATGCGTAGAGATCGGAAAGAACACCAACGGC
GAAAGCACTCTGCTGGGCCGACACTGACACTGAGAGGCGAAAGCTAGGGGAGCGA
ATGGGATTAGATACCCCAGTAGTCCTAGCCGTAAACGATGGATACTAGGCGCTGTGC
GTATCGACCCGTGCAGTGCTGTAGCTAACGCGTTAAGTATCCCGCCTGGGGAGTACG
TTCGCAAGAATGAAACTCAAAGGAATTGACGGGGGCCCGCACAAGCGGTGGAGCAT
GTGGTTTAATTCGATGCAAAGCGAAGAACCTTACCAGGGCTTGACATGCCGCGAATC
CTCTTGAAAGAGAGGTGTGCCTTCGGGAACGCGGACACAGGTGGTGCATGGCTGTC
GTCAGCTCGTGCCGTAAGGTGTTGGGTTAAGTCCCGCAACGAGCGCAACCCTCGTGT
TTAGTTGCCACCGTTGAGTTTGGAACCCTGAACAGACTGCCGGTGATAAGCCGGAGG
AAGGTGAGGATGACGTCAAGTCATCATGCCCCTTATGCCCTGGGCGACACACGTGCT
ACAATGGCCGGGACAAAGGGTCGCGATCCCGCGAGGGTGAGCTAACCCCAAAAACC
CGTCCTCAGTTCGGATTGTAGGCTGCAACTCGCCTGCATGAA
>P021_WA11_k29.262 [Acostemma n. sp. 2, matK (partial)]
ATCCAAGGCTATTTACAGCTTGATAGATCTCAACAGCCCGGCTTTCTATATCCACTTA
TCTTTCAGGAGTATATTTACGGACTTGCTCATGATTATAGTTTAAATCGATCTCGCTT
GTTGAAAAATCCGGGTTATGACAATAAATACAGTTTAC
>P004_WE06_k50.44654 [Athysanus argentarius, 16S]
TCTCACGGAGAGTTTGATCCTGGCTCAGGATGAACGCTGGCGGCATGCTTAACACAT
GCAAGTCGTACGGGAAGTGGTGTTTCCAGTGGCGGACGGGTGAGTAACGCGTAAGA
ACCTGCCCTTGGGAGGGGGATAACAGCTGGAAACGGCTGCTAATACCCCGTATGCT
GAGGAGTAAAAGGAGGAATCCGCCCGAGGAGGGGCTTGCGTCTGATTAGCTAGTTG
GTGAGGCAATAGCTTACCAAGGCGATGATCAGTAGCTGGTCTGAGAGGATGATCAG
CCACACTGGGACTGAGATACGGCCCAGACTCCTACGGGAGGCAGCAGTGGGGAATT
CTCCGCAATGGGCGAAAGCCTGACGGGGCAATGCCGCGTGGAGGTAGAAGGCCTAC
GGGTTGTAAACTCCTTTTCCCGGAGAAGAAACAATGACGGTATCTGGGGAATAAGC
ATCGGCTAACTCTGTGCCAGCAGCCGCGGTAAGACAGAGGATGCAAGCGTTATCCG
GAATTATTGGGCGTAAAGCGTCTGTAGGTGGCTTTTTAAGTCCTCCGTTAAATCCCA

GGGCTCAACCCTGGACAGGCGGTGGAAACTGGCAAGCTGGAGTCCGGTAGGGGCAG
AGGGAATTTCCGGTGGAGCGGTTAAATGCGTTGAGATCGGAAAGAACACCAATGGC
GAAAGCACTCTGCTGGGCCGACACTGACACTGAGAGACGAAAGCTATGGGAGCGA
GTGGGATTAGAGACCCCAATAGTCCTGGCCGTAAACGATGGATACTGGGTGTTGCG
CGTATCGACCCGTGCAGTGCTATAGCTAACGCGTTAAGTATCCCGCCTGGGGAGTAC
GTTCGCAAGAATGAAACTCAAAGGAATTGGCGGGGGCCCGCACAAGCGGTGGAGCA
TGTGGTTTAATTCGATGCAAAGCGAAGAACCTTACCAGGGCTTGACATGCCGCGAAT
CCTCTTGAAAGAGAGGGGTGCCCTCGGGAACGCGGACACAGGTGGTGCATGGCTGT
CGTCAGCTCGTGCCGTGAGGTGTTGGGTTGAGTCCCGCAACGAGCGCAACCCTTGTG
CTGAGTTGCCACCATGGAGTTTGGAACCCTTAGCAGACTGCCGGTGATATAGAATGC
GGAGGAAGGCGAGGATAACGTCAAGTCATCATGCCCCTTATGCCCTGGGCGACACA
CGTGCTACAATGGACGGGACAAAGGATCGCGACCCGCGAGGGTGAGCTAACTCCAA
AAACCCGTCCTCAGTTCGGATTGCAGGCTGCAACTCGCCTGCATGAAGCAGGAATC
GCTAGTAATCGCCGGTCAGCCATACGGCGGTGAATTCGTTCCCGGGCCTTGTACACA
CCGCCCGTCACACTAGGGGAGCTGGCCATGTCCGAAGTCTTTAACCTTAACCGCAAG
GAGGGGTCTGCCCAAGGCACGGCTAGTGACTAAAGTGAAGTCGTAACAAGGTAGCC
GTACTGGAAGGTGTGGCTGGATCACCTCCTTT
>P003_WB04_k90.7617 [Euscelis incisa, 16S]
TCTCATGGAGAGTTCGATCCTGGCTCAGGATGAACGCTGGCGGCATGCTTAACACAT
GCAAGTCGGACGGGAAGTGGTGTTTCCAGTGGCGGACGGGTGAGTAACGCGTAAGA
ACCTGCCCTTGGGAGGGGAACAACAGCTGGAAACGGCTGCTAATACCCCGTAGGCT
GAGGAGCAAAAGGAGGAATCCGCCCGAGGAGGGGCTCGCGTCTGATTAGCTAGTTG
GTGAGGCAATAGCTTACCAAGGCGATGATCAGTAGCTGGTCCGAGAGGATGATCAG
CCACACTGGGACTGAGACACGGCCCAGACTCCTACGGGAGGCAGCAGTGGGGAATT
TTCCGCAATGGGCGAAAGCCTGACGGAGCAATGCCGCGTGGAGGTAGAAGGCCCAC
GGGTCGTGAACTTCTTTTCCCGGAGAAGAAGCAATGACGGTATCTGGGGAATAAGC
ATCGGCTAACTCTGTGCCAGCAGCCGCGGTAATACAGAGGATGCAAGCGTTATCCG
GAATGATTGGGCGTAAAGCGTCTGTAGGTGGCTTTTTAAGTCCGCCGTCAAATCCCA
GGGCTCAACCCTGGACAGGCGGTGGAAACTACCAAGCTGGAGTACGGTAGGGGCAG
AGGGAATTTCCGGTGGAGCGGTGAAATGCGTAGAGATCGGAAAGAACACCAACGGC
GAAAGCACTCTGCTGGGCCGACACTGACACTGAGAGACGAAAGCTAGGGGAGCGA
ATGGGATTAGATACCCCAGTAGTCCTAGCCGTAAACGATGGATACTAGGCGCTGTGC
GTATCGACCCGTGCAGTGCTGTAGCTAACGCGTTAAGTATCCCGCCTGGGGAGTACG
TTCGCAAGAATGAAACTCAAAGGAATTGACGGGGGCCCGCACAAGCGGTGGAGCAT
GTGGTTTAATTCGATGCAAAGCGAAGAACCTTACCAGGGATTGACATGCCGCGAAT
CCTCTTGAAAGAGAGGGGTGCCTTCGGGAACGCGGACACAGGTGGTGCATGGCTGT
CGTCAGCTCGTGCCGTAAGGTGTTGGGTTAAGTCCCGCAACGAGCGCAACCCTCGTG
TTTAGTTGCCACCGTTGAGTTTGGAACCCTGAGCAGACTGCCGGTGATAAGCCGGAG
GAAGGTGAGGATGACGTCAAGTCATCATGCCCCTTATGCCCTGGGCGACACACGTG
CTACAATGGCCGGGACAAAGGGTCGCGATCCCGCGAGGGTGAGCTAACTCCAAAAA
CCCGTCCTCAGTTCGGATTGCAGGCTGCAACTCGCCTGCATGAAGCCGGAATCGCTA
GTAATCGCCGGTCAGCCATACGGCGGTGAATTCGTTCCCGGGCCTTGTACACACCGC
CCGTCACACTATGGGAGCTGGCCATGCCCGAAGTCGTTACCTTAACCGCAAGGAGG
GGGATGCCGAAGGCAGGGCTAGTGACTGGAGTGAAGTCGTAACAAGGTAGCCGTAC
TGGAAGGTGCGGCTGGATCACCTCCTTT
>P008_WE05_k90.2341 [Athysanini New Genus GH4 n. sp. 1, 16S]

TCTCATGGAGAGTTTGATCCTGGCTCAGGATGAACGCTGGCGGCATGCTTAACACAT
GCAAGTCGGACGGGAAGTGGTGTTTCCAGTGGCGGACGGGTGAGTAACGCGTAAGA
ACCTGCCCTTGGGAGGGGAACAACAGCTGGAAACGGCTGCTAATACCCCGTAGGCT
GAGGAGCAAAAGGAGGAATCCGCCCGAGGAGGGGCTCGCGTCTGATTAGCTAGTTG
GTGAGGCAATAGCTTACCAAGGCGATGATCAGTAGCTGGTCCGAGAGGATGATCAG
CCACACTGGGACTGAGACACGGCCCAGACTCCTACGGGAGGCAGCAGTGGGGAATT
TTCCGCAATGGGCGAAAGCCTGACGGAGCAATGCCGCGTGGAGGTAGAAGGCCTAC
GGGTCGTTAACTTCTTTTCCCGGAGAAGAAGGAATGACGGTATCTGGGGAATAAGC
ATCGGCTAACTCTGTGCCAGCAGCCGCGGTAAGACAGAGGATGCAAGCGTTATCCG
GAATGATTGGGCGTAAAGCGTCTGTAGGTGGCTTTTTAAGTTCGCCGTCAAATCCCA
GGGCTCAACCCTGGACAGGCGGTGGAAACTACCAAGCTGGAGTACGGTAGGGGCAG
AGGGAATTTCCGGTGGAGCGGTGAAATGCGTAGAGATCGGAAAGAACACCAACGGC
GAAAGCACTCTGCTGGGCCGACACTGACACTGAGAGACGAAAGCTAGGGGAGCGA
ATGGGATTAGATACCCCAGTAGTCCTAGCCGTAAACGATGGATACTAGGCGCTGTGC
GTATCGACCCGTGCAGTGCTGTAGCTAACGCGTTAAGTATCCCGCCTGGGGAGTACG
TTCGCAAGAATGAAACTCAAAGGAATTGACGGGGGCCCGCACAAGCGGTGGAGCAT
GTGGTTTAATTCGATGCAAAGCGAAGAACCTTACCAGGGCTTGACATGCCGCGAATC
CTCTTGAAAGAGAGGGGTGCCTTCGGGAACGCGGACACAGGTGGTGCATGGCTGTC
GTCAGCTCGTGCCGTAAGGTGTTGGGTTAAGTCCCGCAACGAGCGCAACCCTCGTGT
TTAGTTGCCAACGTTGAGTTTGGAACCCTGAGCAGACTGCCGGTGATAAGCCGGAG
GAAGGTGAGGATGACGTCAAGTCATCATGCCCCTTATGCCCTGGGCGACACACGTG
CTACAATGGCCGGGACAAAGGATCGCGATCCCGCGAGGGTGAGCTAACTCCAAAAA
CCCGTCCTCAGTTCGGATTGTAGGCTGCAACTCGCCTGCATGAAGCCGGAATCGCTA
GTAATCGCCGGTCAGCCATACGGCGGTGAATTCGTTCTCGGGCCTTGTACACACAGC
CCGTCACACTATGGGAGCTGGCCATGCCCGAAGTCGTTACCTTAACCGCAAGGAGG
GGGATGCCGAAGGCAGGGCTAGTGACTGGAGTGAAGTCGTAACAAGGTAGCCGTAC
TGGAAGGTGCGGCTGGATCACCTCCTTT
>P014_WE05_k90.2433 [Athysanini New Genus PE10 n. sp. 1, 16S]
TCTCATGGAGAGTTTGATCCTGGCTCAGGATGAACGCTGGCGGCATGCTTAACACAT
GCAAGTCGGACGGGAAGTGGTGTTTCCAGTGGCGGACGGGTGAGTAACGCGTAAGA
ACCTGCCCTTGGGAGGGGAACAACAGCTGGAAACGGCTGCTAATACCCCATAGGCT
GAGGAGCAAAAGGAGGAATCCGCCCAAGGAGGGGCTCGCGTCTGATTAGTTAGTTG
GTGAGGCAATGGCTTACCAAGGCGACGATCAGTAGCTGGTCCGAGAGGATGATCAG
CCACACTGGGACTGAGACACGGCCCAGACTCCTACGGGAGGCAGCAGTGGGGAATT
TTCCGCAATGGGCGAAAGCCTGACGGAGCAATGCCGCGTGAAGGCAGAAGGCCCAC
GGGTCATGAACTTCTTTTCTCGGAGAAGAAACAATGACGGTATCTGAGGAATAAGC
ATCGGCTAACTCTGTGCCAGCAGCCGCGGTAAGACAGAGGATGCAAGCGTTATCCG
GAATGATTGGGCGTAAAGCGTCTGTAGGTGGCTTTTCAAGTCCGCCGTCAAATCCCA
GGGCTCAACCCTGGACAGGCGGTGGAAACTACCAAGCTGGAGTACGGTAGGGGCAG
AGGGAATTTCCGGTGGAGCGGTGAAATGCGTTGAGATCGGAAAGAACACCAACGGC
GAAAGCACTCTGCTGGGCCGACACTGACACTGAGAGACGAAAGCTAGGGGAGCAA
ATGGGATTAGATACCCCAGTAGTCCTAGCCGTAAACGATGGATACTAAGTGCTGTGC
GTATCGACCCGCGCAGTGCTGTAGCTAACGCGTTAAGTATCCCGCCTGGGGAGTACG
TTCGCAAGAATGAAACTCAAAGGAATTGACGGGGGCCCGCACAAGCGGTGGAGCAT
GTGGTTCAATTCGATGCAAAGCGAAGAACCTTACCAGGGCTTGACATGCCGTGAATC
CTCCCGAAAGAGAGGAGTGCCTTCGGGAACGCGGACACAGGTGGTGCATGGCTGTC

GTCAGCTCGTGCCGTAAGGTGTTGGGTTAAGTCCCGCAACGAGCGCAACCCTCGTGT
TTAGTTGCCAGCATTGAGTTTGGAACCCTGAACAGACTGCCGGTGATAAGCCGGAG
GAAGGTGAGGATGACGTCAAGTCATCATGCCCCTTACGCCCTGGGCGACACACGTG
CTACAATGACCGGGACAAAGGGTCGCGACCCCGCGAGGGCAAGCTAACCTCAAAAA
CCCGGCCTCAGTTCGGATTGCAGGCTGCAACTCGCCTGCATGAAGCCGGAATCGCTA
GTAATCGCCGGTCAGCCATACGGCGGTGAATCCGTTCCCGGGCCTTGTACACACCGC
CCGTCACACTATGGGAGCTGGCCATGCCCCAAGTCGTTACCTTAACCGCAAGGAGG
GGGATGCCGAAGGCTGGGCTAGTGACTGGAGTGAAGTCGTAACAAGGTAGCCGTAC
TGGAAGGTGCGGCTGGATCACCTCCTTT
>P007_WA01_k90.1332 [Athysanini New Genus ZA2 n. sp. 1, 16S]
TCTCATGGAGAGTTCGATCCTGGCTCAGGATGAACGCTGGCGGCATGCCTTACACAT
GCAAGTCGGACGGGAAGTGGTGTTTCCAGTGGCGGACGGGTGAGTAACGCGTAAGA
ACCTACCCTTGGGAGGGGAACAACAGCTGGAAACGGCTGCTAATACCCCGTAGGCT
GAGGAGCAAAAGGAGGAATCCGCCCGAGGAGGGGCTCGCGTCTGATTAGCTAGTTG
GTGAGGCAATAGCTTACCAAGGCGATGATCAGTAGCTGGTCCGAGAGGATGATCAG
CCACACTGGGACTGAGACACGGCCCAGACTCCTACGGGAGGCAGCAGTGGGGAATT
TTCCGCAATGGGCGAAAGCCTGACGGAGCAATGCCGCGTGAAGGTAGAAGGCCTAC
GGGTCATGAACTTCTTTTCCCGGAGAAGAAGCAATGACGGTATCCGGGGAATAAGC
ATCGGCTAACTCTGTGCCAGCAGCCGCGGTAAGACAGAGGATGCAAGCGTTATCCG
GAATGATTGGGCGTAAAGCGTCTGTAGGTGGCTTTTTAAGTTCGCCGTCAAATCCCA
GGGCTCAACCCTGGACAGGCGGTGGAAACTACCAAGCTGGAGTACGGTAGGGGCAG
AGGGAATTTCCGGTGGAGCGGTGAAATGCGTAGAGATCGGAAAGAACACCAACGGC
GAAAGCACTCTGCTGGGCCGACACTGACACTGAGAGACGAAAGCTAGGGGAGCGA
ATGGGATTAGATACCCCAGTAGTCCTAGCCGTAAACGATGGATACTAGGCGCTGTGC
GTATCGACCCGTGCAATGCTGTAGCTAACGCGTTAAGTATCCCGCCTGGGGAGTACG
TTCGCAAGAATGAAACTCAAAGGAATTGACGGGGGCCCGCACAAGCGGTGGAGCAT
GTGGTTTAATTCGATGCAAAGCGAAGAACCTTACCAGGGCTTGACATGCCGCGAATC
CTCTTGAAAGAGAGGGGTGCCTTCGGGAACGCGGACACAGGTGGTGCATGGCTGTC
GTCAGCTCGTGCCGTAAGGTGTTGGGTTAAGTCCCGCAACGAGCGCAACCCTCGTGT
TTAGTTGCCAACATTTAGTTTGGAACCCTGAGCAGACTGCCGGTGATAAGCCGGAGG
AAGGTGAGGATGACGTCAAGTCATCATGCCCCTTATGCCCTGGGCGACACACGTGCT
ACAATGGACGGGACAAAGGATCGCGATCCCGCGAGGGTGAGCTAACTCCAAAAACC
CGTCCTCAGTTCGGATTGTAGGCTGCAACTCGCCTGCATGAAGCCGGAATCGCTAGT
AATCGCCGGTCAGCCATACGGCGGTGAATTCGTTCCCGGGCCTTGTACACACCGCCC
GTCACACTATGGGAGCTGGCCATGCCCGAAGTCGTTACCTTAACCGCAAGGAGGGG
GATGCCGAAGGCAGGGCTAGTGACTGGAGTGAAGTCGTAACAAGGTAGCCGTACTG
GAAGGTGCGGCTGGATCACCTCCTTT
>P015_WE07_k90.5204 [Ollarianus strictus, 16S]
TCTCATGGAGAGTTCGATCCTGGCTCAGGATGAACGCTGGCGGCATGCTTAACACAT
GCAAGTCGGACGGGAAGTGGTGTTTCCAGTGGCGGACGGGTGAGTAACGCGTAAGA
ACCTGCCCTTGGGAGGGGAACAACAGCTGGAAACGGCTGCTAATACCCCGTAGGCT
GAGGAGCAAAAGGAGGAATCCGCCCGAGGAGGGGCTCGCGTCTGATTAGCTAGTTG
GTGAGGCAATAGCTTACCAAGGCGATGATCAGTAGCTGGTCCGAGAGGATGATCAG
CCACACTGGGACTGAGACACGGCCCAGACTCCTACGGGAGGCAGCAGTGGGGAATT
TTCCGCAATGGGCGAAAGCCTGACGGAGCAATGCCGCGTGGAGGTAGAAGGCCCAC
GGGTCGTGAACTTCTTTTCCCGGAGAAGAAGCAATGACGGTATCTGGGGAATAAGC

ATCGGCTAACTCTGTGCCAGCAGCCGCGGTAATACAGAGGATGCAAGCGTTATCCG
GAATGATTGGGCGTAAAGCGTCTGTAGGTGGCTTTTAAGTCCGCCGTCAAATCCCA
GGGCTCAACCCTGGACAGGCGGTGGAAACTACCAAGCTGGAGTACGGTAGGGGCAG
AGGGAATTTCCGGTGGAGCGGTGAAATGCGTAGAGATCGGAAAGAACACCAACGGC
GAAAGCACTCTGCTGGGCCGACACTGACACTGAGAGGCGAAAGCTAGGGGAGCGA
ATGGGATTAGATACCCCAGTAGTCCTAGCCGTAAACGATGGATACTAGGCGCTGTGC
GTATCGACCCGTGCAGTGCTGTAGCTAACGCGTTAAGTATCCCGCCTGGGGAGTACG
TTCGCAAGAATGAAACTCAAAGGAATTGACGGGGGCCCGCACAAGCGGTGGAGCAT
GTGGTTTAATTCGATGCAAAGCGAAGAACCTTACCAGGGCTTGACATGCCGCGAATC
CTCTTGAAAGAGAGGTGTGCCTTCGGGAACGCGGACACAGGTGGTGCATGGCTGTC
GTCAGCTCGTGCCGTAAGGTGTTGGGTTAAGTCCCGCAACGAGCGCAACCCTCGTGT
TTAGTTGCCACCGTTGAGTTTGGAACCCTGAACAGACTGCCGGTGATAAGCCGGAGG
AAGGTGAGGATGACGTCAAGTCATCATGCCCCTTATGCCCTGGGCGACACACGTGCT
ACAATGGCCGGGACAAAGGGTCGCGATCCCGCGAGGGTGAGCTAACTCCAAAAACC
CGTCCTCAGTTCGGATTGCAGGCTGCAACTCGCCTGCATGAAGCCGGAATCGCTAGT
AATCGCCGGTCAGCCATACGGCGGTGAATTCGTTCCCGGGCCTTGTACACACCGCCC
GTCACACTATGGGAGCTGGCCATGCCCGAAGTCGTTACCTTAACCGCAAGGAGGGG
GATGCCGAAGGCAGGGCTAGTGACTGGAGTGAAGTCGTAACAAGGTAGCCGTACTG
GAAGGTGCGGCTGGATCACCTCCTTT
>P001_WC10_k90.15932 [Thamnotettix dilutior, 16S (partial)]
AGGCCTACGGGTCGTGAACTTCTTTTCCCGGAGAAGAAGCAATGACGGTATCTGGG
GAATAAGCATCGGCTAACTCTGTGCCAGCAGCCGCGGTAATACAGAGGATGCAAGC
GTTATCCGGAATGATTGGGCGTAAAGCGTCTGTAGGTGGCTTTTAAGTCCGCCGTC
AAATCCCAGGGCTCAACTCTGGACAGGCGGTGGAAACTACCAAGCTGGAGTACGGT
AGGGGCAGAGGGAATTTCCGGTGGAGCGGTGAAATGCGTAGAGATCGGAAAGAAC
ACCAACGGCGAAAGCACTCTGCTGGGCCGACACTGACACTGAGAGACGAAAGCTAG
GGGAGCGAATGGGATTAGATACCCCAGTAGTCCTAGCCGTAAACGATGGATACTAG
GCGCTGTGCGTATCGACCCGTGCAGTGCTGTAGCTAACGCGTTAAGTATCCCGCCTG
GGGAGTACGTTCGCAAGAATGAAACTCAAAGGAATTGACGGGGGCCCGCACAAGCG
GTGGAGCATGTGGTTTAATTCGATGCAAAGCGAAGAACCTTACCAGGGCTTGACAT
GCCGCGAATCCTCTTGAAAGAGAGGGGTGCCTTCGGGAACGCGGACACAGGTGGTG
CATGGCTGTCGTCAGCTCGTGCCGTAAGGTGTTGGGTTAAGTCCCGCAACGAGCGCA
ACCCTCGTGTTTAGTTGCCATCATTGAGTTTGGAACCCTGAACAGACTGCCGGTGAT
AAGCCGGAGGAAGGTGAGGATGACGTCAAGTCATCATGCCCCTTATGCCCTGGGCG
ACACACGTGCTACAATGGCCGGGACAAAGGGTCGCGATCCCGCGAGGGTGAGCTAA
CTCCAAAAACCCGTCCTCAGTTCGGATTGCAGGCTGCAACTCGCCTGCATGAAGCCG
GAATCGCTAGTAATCGCCGGTCAGCCATACGGCGGTGAATCCGTTCCCGGGCCTTGT
ACACACCGCCCGTCACACTATGGGAGCTGGCCATGCCCGAAGTCGTTACCTTAACCG
CAAGGAGGGGGATGCCGAAGGCAGGGCTAGTGACTGGAGTGAAGTCGTAACAAGG
TAGCCGTACTGGAAGGTGCGGCTGGATCACCTCCTTT
>P006_WD11_k90.19170 [Bonaspeiini New Genus SA3 n. sp., 16S]
TCTCATGGAGAGTTCGATCCTGGCTCAGGATGAACGCTGGCGGCATGCTTGACACAT
GCAAGTCGGACGGGAAGTGGTGTTTCCAGTGGCGGACGGGTGAGTAACGCGTAAGA
ACCTGCCCTTGGGAGGGGAACAACAGCTGGAAACGGCTGCTAATACCCCGTAGGCT
GAGGGGCAAAAAGGAGGAATCCGCCCGAGGAGGGGCTCGCGTCTGATTAGCTAGTT
GGTGAGGCAATAGCTTACCAAGGCTATGATCAGTAGCTGGTCCGAGAGGATGATCA

GCCACACTGGGACTGAGACACGGCCCAGACTCCTACGGGAGGCAGCAGTGCGGAAT
TTTCCGCAATGGGCGAAAGCCTGACGGAGCAATGCCGCGTGGAGGTAGAAGGCCCA
CGGGTCGTAAACTTCTTTTCCCGGAGAAGAAGCAATGACGGTATCTGGGGAATAAG
CATCGGCTAACTCTGTGCCAGCAGCCGCGGTAAGACAGAGGATGCAAGCGTTATCC
GGAATGATTGGGCGTAAAGCGTCTGTAGGTGGCTTTTTAAGTCCGCCGTCAAATCCC
AGGGCTCAACCCTGGACAGGCGGTGGAAACTACCAAGCTGGAGTACGGTAGGGGCA
GAGGGAATTTCCGGTGGAGCGGTGAAATGCGTAGAGATCGGAAAGAACACCAACG
GCGAAAGCACTCTGCTGGGCCGACACTGACACTGAGAGACGAAAGCTAGGGGAGC
GAGCGGGATTAGAGACCCCAGTAGTCCTAGCCGTAAACGATGGATACTAGGTGCTG
TGCGTATCCACCCGTGCAGTGCTGTAGCTAACGCGTTAAGTATCCCGCCTGGGGAGT
ACGTTCGCAAGAATGAAACTCAAAGGAATTGACGGGGGCCCGCACAAGCGGTGGAG
CATGTGGTTTAATTCGATGCAAAGCGAAGAACCTTACCAGGGCTTGACATGCCGCGA
ACCCTCTTGAAAGAGAGGGGCGCCTTCGGGAACGCGGACACAGGTGGTGCATGGCT
GTCGTCAGCTCGTGCCGTAAGGTGTTGGGTTAAGTCCCGCAACGAGCGCAACCCTCG
TGTTTAGTTGCCATCGTTGAGTTTGGAACCCTGAACAGACTGCCGGTGATAAGCCGG
AGGAAGGTGAGGATGACGTCAAGTCATCATGCCCCTTATGCCCTGGGCGACACACG
TGCTACAATGGCCGGGACAAAGGGTCGCGATCCCGCGAGGGTTAGCTAACTCCAAA
AACCCGTCCTCAGTTCGGATTGCAGGCTGCAACTCGCCTGCATGAAGCCGGAATCGC
TAGTAATCGCCGGTCAGCCATACGGCGGTGAATTCGTTCCCGGGCCTTGTACACACC
GCCCGTCACACTATGGGAGCTGGCCATGTCCGAAGTCGTTACCTTAACCGTCGGAGG
GGTATGCCGAAGGCAGGGCTAGTGATCGGAGTGAAGTCGTAACAAGGTAGCCGTAC
TGGAAGGTGCGGCTGGATCACCTCCTTT
>P007_WD11_k90.32499 [Aconurella compta, 16S (partial)]
CCAAGCTGGAGTACGGTAGGGGCAGAGGGAATTTCCGGTGGAGCGGTGAAATGCGT
AGAGATCGGAAAGAACACCAACGGCGAAAGCACTCTGCTGGGCCGACACTGACACT
GAGAGACGAAAGCTAGGGGAGCGAATGGGATTAGATACCCCAGTAGTCCTAGCCGT
AAACGATGGATACTAGGCGCTGTGCGTATCGACCCGTGCAGTGTTGTAGCTAACGCG
TTAAGTATCCCGCCTGGGGAGTACGTTCGCAAGAATGAAACTCAAAGGAATTGACG
GGGGCCCGCACAAGCGGTGGAGCATGTGGTTTAATTCGATGCAAAGCGAAGAACCT
TACCAGGGCTTGACATGCCGCGAATCCTCTTGAAAGAGAGGGGTGCCTTCGGGAAC
GCGGACACAGGTGGTGCATGGCTGTCGTCAGCTCGTGCCGTAAGGTGTTGGGTTAAG
TCCCGCAACGAGCGCAACCCTCGTGTTTAGTTGCCGACGTTGAGTTTGGAACCCTGA
ACAGACTGCCGGTGATAAGCCGGAGGAAGGTGAGGATGACGTCAAGTCATCATGCC
CCTTATGCCCTGGGCGACACACGTGCTACAATGGCCGGGACAAAGGGTCGCGATCC
CGCGAGGGTGAGCTAACCCCAAAAACCCGTCCTCAGTTCGGATTGCAGGCTGCAAC
TCGCCTGCATGAAGCCGGAATCGCTAGTAATCGCCGGTCAGCCATACGGCGGTGAA
TTCGTTCCCGGGCCTTGTACACACCGCCCGTCACACTATGGGAGCTGGCCATGCCCG
AAGTCGTTACCTTAACCGCAAGGAGGGGGATGCCGAAGGC
>P004_WH04_k90.4463 [Aconurella prolixa, 16S (partial)]
AGAACACCAACGGCGAAAGCACTCTGCTGGGCCGACACTGACACTGAGAGACGAA
AGCTAGGGGAGCGAATGGGATTAGATACCCCAGTAGTCCTAGCCGTAAACGATGGA
TACTAGGCGCTGTGCGTATCGACCCGTGCAGTGCTGTAGCTAACGCGTTAAGTATCC
CGCCTGGGGAGTACGTTCGCAAGAATGAAACTCAAAGGAATTGACGGGGGCCCGCA
CAAGCGGTGGAGCATGTGGTTTAATTCGATGCAAAGCGAAGAACCTTACCAGGGCT
TGACATGCCGCGAATCCTCTTGAAAGAGAGGGGTGCCTTCGGGAACGCGGACACAG
GTGGTGCATGGCTGTCGTCAGCTCGTGCCGTAAGGTGTTGGGTTAAGTCCCGCAACG

AGCGCAACCCTCGTGTTTAGTTGCCATCGTTGAGTTTGGAACCCTGAACAGACTGCC
GGTGATAAGCCGGAGGAAGGTGAGGATGACGTCAAGTCATCATGCCCCTTATGCCC
TGGGCGACACACGTGCTACAATGGCCGGGACAAAGGGTCGCGATCCCGCGAGGGTG
AGCTAACTCCAAAAACCCGTCCTCAGTTCGGATTGCAGGCTGCAACTCGCCTGCATG
AAGCCGGAATCGCTAGTAATCGCCGGTCAGCCATACGGCGGTGAATTCGTTCCCGG
GCCTTGTACACACCGCCCGTCACACTATGGGAGCTGGCCATGCCCGAAGTCGTTACC
TTAACCGCAAGGAGGGGGATGCCGAAGGCAGGGCTAGTGACTGGAGTGAAGTCGTA
ACAAGGTAGCCGTACTGGAAGGTGCGGCTGGATCACCTCCTTT
>P015_WE09_k90.804 [Exitianus apophysisiosus, 16S]
TCTCATGGAGAGTTCGATCCTGGCTCAGGATGAACGCTGGCGGCATGCTTAACACAT
GCAAGTCGGACGGGAAGTGGTGTTTCCAGTGGCGGACGGGTGAGTAACGCGTAAGA
ACCTGCCCTTGGGAGGGGAACAACAGCTGGAAACGGCTGCTAATACCCCGTAGGCT
GAGGAGCAAAAGGAGGAATCCGCCCGAGGAGGGGCTCGCGTCTGATTAGCTAGTTG
GTGAGGCAATAGCTTACCAAGGCGATGATCAGTAGCTGGTCCGAGAGGATGATCAG
CCACACTGGGACTGAGACACGGCCCAGACTCCTACGGGAGGCAGCAGTGGGGAATT
TTCCGCAATGGGCGAAAGCCTGACGGAGCAATGCCGCGTGGAGGTAGAAGGCCCAC
GGGTCGTGAACTTCTTTTCCCGGAGAAGAAGCAATGACGGTATCTGGGGAATAAGC
ATCGGCTAACTCTGTGCCAGCAGCCGCGGTAATACAGAGGATGCAAGCGTTATCCG
GAATGATTGGGCGTAAAGCGTCTGTAGGTGGCTTTTTAAGTCCGCCGTCAAATCCCA
GGGCTCAACCCTGGACAGGCGGTGGAAACTACCAAGCTGGAGTACGGTAGGGGCAG
AGGGAATTTCCGGTGGAGCGGTGAAATGCGTAGAGATCGGAAAGAACACCAACGGC
GAAAGCACTCTGCTGGGCCGACACTGACACTGAGAGGCGAAAGCTAGGGGAGCGA
ATGGGATTAGATACCCCAGTAGTCCTAGCCGTAAACGATGGATACTAGGCGCTGTGC
GTATCGACCCGTGCAGTGCTGTAGCTAACGCGTTAAGTATCCCGCCTGGGGAGTACG
TTCGCAAGAATGAAACTCAAAGGAATTGACGGGGGCCCGCACAAGCGGTGGAGCAT
GTGGTTTAATTCGATGCAAAGCGAAGAACCTTACCAGGGCTTGACATGCCGCGAATC
CTCTTGAAAGAGAGGTGTGCCTTCGGGAACGCGGACACAGGTGGTGCATGGCTGTC
GTCAGCTCGTGCCGTAAGGTGTTGGGTTAAGTCCCGCAACGAGCGCAACCCTCGTGT
TTAGTTGCCACCGTTGAGTTTGGAACCCTGAACAGACTGCCGGTGATAAGCCGGAGG
AAGGTGAGGATGACGTCAAGTCATCATGCCCCTTATGCCCTGGGCGACACACGTGCT
ACAATGGCCGGGACAAAGGGTCGCGATCCCGCGAGGGTGAGCTAACTCCAAAAACC
CGTCCTCAGTTCGGATTGCAGGCTGCAACTCGCCTGCATGAAGCCGGAATCGCTAGT
AATCGCCGGTCAGCCATACGGCGGTGAATTCGTTCCCGGGCCTTGTACACACCGCCC
GTCACACTATGGGAGCTGGCCATGCCCGAAGTCGTTACCTTAACCGCAAGGAGGGG
GATGCCGAAGGCAGGGCTAGTGACTGGAGTGAAGTCGTAACAAGGTAGCCGTACTG
GAAGGTGCGGCTGGATCACCTCCTTT
>P011_WE06_k90.1223 [Nephotettix modulatus, 16S (partial)]
TGCTTAACACATGCAAGTCGGACGGGAAGTGGTGTTTCCAGTGGCGGACGGGTGAG
TAACGCGTAAGAACCTGCCCTTGGGAGGGGAACAACAGCTGGAAACGGCTGCTAAT
ACCCCGTAGGCTGAGGAGCAAAAGGAGGAATCCGCCCGAGGAGGGGCTCGCGTCTG
ATTAGCTAGTTGGTGAGGCAATAGCTTACCAAGGCGATGATCAGTAGCTGGTCCGA
GAGGATGATCAGCCACACTGGGACTGAGACACGGCCCAGACTCCTACGGGAGGCAG
CAGTGGGGAATTTTCCGCAATGGGCGAAAGCCTGACGGAGCAATGCCGCGTGGAGG
TAGAAGGCCTACGGGTCGTGAACTTCTTTTCCCGGAGAAGAAGCAATGACGGTATCT
GGGGAATAAGCATCGGCTAACTCTGTGCCAGCAGCCGCGGTAAGACAGAGGATGCA
AGCGTTATCCGGAATGATTGGGCGTAAAGCGTCTGTAGGTGGCTTTTTAAGTCCGCC

GTCAAATCCCAGGGCTCAACCCTGGACAGGCGGTGGAAACTACCAAGCTGGAGTAC
GGTAGGGGCAGAGGGAATTTCCGGTGGAGCGGTGAAATGCGTAGAGATCGGAAAG
AACACCAACGGCGAAAGCACTCTGCTGGGCCGACACTGACACTGAGAGACGAAAGC
TAGGGGAGCGAATGGGATTAGATACCCCAGTAGTCCTAGCCGTAAACGATGGATAC
TAGGCGCTGTGCGTATCGACCCGTGCAGTGCTGTAGCTAACGCGTTAAGTATCCCGC
CTGGGGAGTACGTTCGCAAGAATGAAACTCAAAGGAATTGACGGGGGCCCGCACAA
GCGGTGGAGCATGTGGTTTAATTCGATGCAAAGCGAAGAACCTTACCAGGGCTTGA
CATGCCGCGAATCCTCTTGAAAGAGAGGGGTGCCTTCGGGAACGCGGACACAGGTG
GTGCATGGCTGTCGTCAGCTCGTGCCGTAAGGTGTTGGGTTAAGTCCCGCAACGAGC
GCAACCCTCGTGTTTAGTTGCCACCGTTGAGTTTGGAACCCTGAGCAGACTGCCGGT
GATAAGCCGGAGGAAGGTGAGGATGACGTCAAGTCATCATGCCCCTTATGCCCTGG
GCGACACACGTGCTACAATGGCCGGGACAAAGGGTCGCGACCCCGCGAGGGTGAGC
TAACTCCAAAAACCCGTC
>P015_WE06_k90.1930 [Cicadula sp., 16S]
TCTCATGGAGAGTTCGATCCTGGCTCAGGATGAACGCTGGCGGCATGCTTAACACAT
GCAAGTCGGACGGGAAGTGGTGTTTCCAGTGGCGGACGGGTGAGTAACGCGTAAGA
ACCTGCCCTTGGGAGGGGAACAACAGCTGGAAACGGCTGCTAATACCCCGTAGGCT
GAGGAGCAAAAGGAGGAATCCGCCCGAGGAGGGGCTCGCGTCTGATTAGCTAGTTG
GTGAGGCAATAGCTTACCAAGGCGATGATCAGTAGCTGGTCCGAGAGGATGATCAG
CCACACTGGGACTGAGACACGGCCCAGACTCCTACGGGAGGCAGCAGTGGGGAATT
TTCCGCAATGGGCGAAAGCCTGACGGAGCAATGCCGCGTGGAGGTAGAAGGCCCAC
GGGTCGTGAACTTCTTTTCCCGGAGAAGAAGCAATGACGGTATCTGGGGAATAAGC
ATCGGCTAACTCTGTGCCAGCAGCCGCGGTAATACAGAGGATGCAAGCGTTATCCG
GAATGATTGGGCGTAAAGCGTCTGTAGGTGGCTTTTTAAGTCCGCCGTCAAATCCCA
GGGCTCAACCCTGGACAGGCGGTGGAAACTACCAAGCTGGAGTACGGTAGGGGCAG
AGGGAATTTCCGGTGGAGCGGTGAAATGCGTAGAGATCGGAAAGAACACCAACGGC
GAAAGCACTCTGCTGGGCCGACACTGACACTGAGAGGCGAAAGCTAGGGGAGCGA
ATGGGATTAGATACCCCAGTAGTCCTAGCCGTAAACGATGGATACTAGGCGCTGTGC
GTATCGACCCGTGCAGTGCTGTAGCTAACGCGTTAAGTATCCCGCCTGGGGAGTACG
TTCGCAAGAATGAAACTCAAAGGAATTGACGGGGGCCCGCACAAGCGGTGGAGCAT
GTGGTTTAATTCGATGCAAAGCGAAGAACCTTACCAGGGCTTGACATGCCGCGAATC
CTCTTGAAAGAGAGGTGTGCCTTCGGGAACGCGGACACAGGTGGTGCATGGCTGTC
GTCAGCTCGTGCCGTAAGGTGTTGGGTTAAGTCCCGCAACGAGCGCAACCCTCGTGT
TTAGTTGCCACCGTTGAGTTTGGAACCCTGAACAGACTGCCGGTGATAAGCCGGAGG
AAGGTGAGGATGACGTCAAGTCATCATGCCCCTTATGCCCTGGGCGACACACGTGCT
ACAATGGCCGGGACAAAGGGTCGCGATCCCGCGAGGGTGAGCTAACTCCAAAAACC
CGTCCTCAGTTCGGATTGCAGGCTGCAACTCGCCTGCATGAAGCCGGAATCGCTAGT
AATCGCCGGTCAGCCATACGGCGGTGAATTCGTTCCCGGGCCTTGTACACACCGCCC
GTCACACTATGGGAGCTGGCCATGCCCGAAGTCGTTACCTTAACCGCAAGGAGGGG
GATGCCGAAGGCAGGGCTAGTGACTGGAGTGAAGTCGTAACAAGGTAGCCGTACTG
GAAGGTGCGGCTGGATCACCTCCTTT
>P015_WE06_k29.63010 [Cicadula sp., matK (partial)]
AATATATACTTTGACCTTGTGTTAAAACTTTGGCTCGTAAACACAAAAAGACTATAC
GCACTTGTTAAAAAAGATTAGGTTTGGAATTTTTGGATTTAGATGCTTCTTCTATTAT
TTTCTTTTCCTCGGCGCAGCTAGGCCATCCTGGACT
>P008_WA06_k90.6039 [Cicadulini New Genus SA1 n. sp., 16S (partial)]

AAGCATCGGCTAACTCTGTGCCAGCAGCCGCGGTAAGACAGAGGATGCAAGCGTTA
TCCGGAATGATTGGGCGTAAAGCGTCTGTAGGTGGCTTTTCAAGTCCGCCGTCAAAT
CCCAGGGCTCAACCCTGGACAGGCGGTGGAAACTACCAAGCTGGAGTACGGTAGGG
GCAGAGGGAATTTCCGGTGGAGCGGTGAAATGCGTAGAGATCGGAAAGAACACCA
ACGGCGAAAGCACTCTGCTGGGCCGACACTGACACTGAGAGACGAAAGCTAGGGG
AGCGAATGGGATTAGATACCCCAGTAGTCCTAGCCGTAAACGATGGATACTAGGCG
CTGTGCGTATCGACCCGTGCAGTGCTGTAGCTAACGCGTTAAGTATCCCGCCTGGGG
AGTACGTTCGCAAGAATGAAACTCAAAGGAATTGACGGGGGCCCGCACAAGCGGTG
GAGCATGTGGTTTAATTCGATGCAAAGCGAAGAACCTTACCAGGGCTTGACATGCC
GCGAATCCTCTTGAAAGAGAGGGGTGCCTTCGGGAACGCGGACACAGGTGGTGCAT
GGCTGTCGTCAGCTCGTGCCGTAAGGTGTTGGGTTAAGTCCCGCAACGAGCGCAACC
CTCGTGTTTAGTTGCCATCATTGAGTTTGGAACCCTGAACAGACTGCCGGTGATAAG
CCGGAGGAAGGTGAGGATGACGTCAAGTCATCATGCCCCTTATGCCCTGGGCGACA
CACGTGCTACAATGGCCGGGACAAAGGGTCGCGATCCCGCGAGGGTGAGCTAACTC
CAAAAACCCGTCCTCAGTTCGGATTGCAGGCTGCAACTCGCCTGCATGAAGCCGGA
ATCGCTAGTAATCGCCGGTCAGCCATACGGCGGTGAATTCGTTCCCGGGCCTTGTAC
ACACCGCCCGTCACACTATGGGAGCTGGCCATGCCCGAAGTCGTTACCTTAACCGCA
AGGAGGGGGATGCCGAAGGCAGGGCTAGTGACTGGAGTGAAGTCGTAACAAGGTA
GCCGTACTGGAAGGTGCGGCTGGATCACCTCCTTT
>P007_WH12_k90.1652 [Loeia tamtipi, 16S (partial)]
TCTCATGGAGAGTTCGATCCTGGCTCAGGATGAACGCTGGCGGCATGCCTTACACAT
GCAAGTCGGACGGGAAGTGGTGTTTCCAGTGGCGGACGGGTGAGTAACGCGTAAGA
ACCTACCCTTGGGAGGGGAACAACAGCTGGAAACGGCTGCTAATACCCCGTAGGCT
GAGGAGCAAAAGGAGGAATCCGCCCGAGGAGGGGCTCGCGTCTGATTAGCTAGTTG
GTGAGGCAATAGCTTACCAAGGCGATGATCAGTAGCTGGTCCGAGAGGATGATCAG
CCACACTGGGACTGAGACACGGCCCAGACTCCTACGGGAGGCAGCAGTGGGGAATT
TTCCGCAATGGGCGAAAGCCTGACGGAGCAATGCCGCGTGAAGGTAGAAGGCCTAC
GGGTCATGAACTTCTTTTCCCGGAGAAGAAGCAATGACGGTATCCGGGGAATAAGC
ATCGGCTAACTCTGTGCCAGCAGCCGCGGTAAGACAGAGGATGCAAGCGTTATCCG
GAATGATTGGGCGTAAAGCGTCTGTAGGTGGCTTTTTAAGTTCGCCGTCAAATCCCA
GGGCTCAACCCTGGACAGGCGGTGGAAACTACCAAGCTGGAGTACGGTAGGGGCAG
AGGGAATTTCCGGTGGAGCGGTGAAATGCGTAGAGATCGGAAAGAACACCAACGGC
GAAAGCACTCTGCTGGGCCGACACTGACACTGAGAGACGAAAGCTAGGGGAGCGA
ATGGGATTAGATACCCCAGTAGTCCTAGCCGTAAACGATGGATACTAGGCGCTGTGC
GTATCGACCCGTGCAATGCTGTAGCTAACGCGTTAAGTATCCCGCCTGGGGAGTACG
TTCGCAAGAATGAAACTCAAAGGAATTGACGGGGGCCCGCACAAGCGGTGGAGCAT
GTGGTTTAATTCGATGCAAAGCGAAGAACCTTACCAGGGCTTGACATGCCGCGAATC
CTCTTGAAAGAGAGGGGTGCCTTCGGGAACGCGGACACAGGTGGTGCATGGCTGTC
GTCAGCTCGTGCCGTAAGGTGTTGGGTTAAGTCCCGCAACGAGCGCAACCCTCGTGT
TTAGTTGCCAACATTTAGTTTGGAACCCTGAGCAGACTGCCGGTGATAAGCCGGAGG
AAGGTGAGGATGACGTCAAGTCATCATGCCCCTTATGCCCTGGGCGACACACGTGCT
ACAATGGACGGGACAAAGGATCGCGATCCCGCGAGGGTGAGCTAACTCCAAAAACC
CGTCCTCAGTTCGGATTGTAGGCTGCAACTCGCCTGCATGAAGCCGGAATCGCTAGT
AATCGCCGGTCAGCCATACGGCGGTGAATTCGTTCCCGGGCCTTGTACACACCGCCC
GTCACACTATGGGAGCTGGCCATGCCCGAAGTCGTTACCTTAACCGCAAGGAGGGG

GATGCCGAAGGCAGGGCTAGTGACTGGAGTGAAGTCGTAACAAGGTAGCCGTACTG
GAAGGTGCG
>P021_WA04_k50.1850 [Deltocephalini New Genus n. sp., matK (partial)]
CAAAATCTTCTACCTTTGGTTCAAATCTAATATCAAATGGAGGAAATCCAAAGATAT
TTACAGCTTGATAGATCTCAACAACACGGCTTTCTATATCCACTTATCTTTCAGGAGT
ATATTTATGCACTTGCTCATGATCATATTTTAAACCGATCTATTTTGTTAGATCGGAA
GAGCACACGTCTGAACTCCAGTCACCAGATCTGATCTC
>P008_WC05_k90.262 [Deltocephalini New Genus PE2 n. sp. 1, 16S]
TCTCATGGAGAGTTCGATCCTGGCTCAGGATGAACGCTGGCGGCATGCTTAACACAT
GCAAGTCGAACGGGAAGTGGTGTTTCCAGTGGCGAACGGGTGAGTAACGCGTAAGA
ACCTGCCCTTGGGAGGGGAACAACAACTGGAAACGGTTGCTAATACCCCGTAGGCT
GAGGAGCAAAAGGAGAAATCCGCCCAAGGAGGGGCTCGCGTCTGATTAGCTAGTTG
GTGAGGCAATAGCTTACCAAGGCGATGATCAGTAGCTGGTCCGAGAGGATGATCAG
CCACACTGGGACTGAGACACGGCCCAGACTCCTACGGGAGGCAGCAGTGGGGAATT
TTCCGCAATGGGCGAAAGCCTGACGGAGCAATGCCGCGTGGAGGTGGAAGGCCTAC
GGGTCGTCAACTTCTTTTCTCGGAGAAGAAACAATGACGGTATCTGAGGAATAAGC
ATCGGCTAACTCTGTGCCAGCAGCCGCGGTAAGACAGAGGATGCAAGCGTTATCCG
GAATGATTGGGCGTAAAGCGTCTGTAGGTGGCTTTTCAAGTCCGCCGTCAAATCCCA
GGGCTCAACCCTGGACAGGCGGTGGAAACTACCAAGCTGGAGTACGGTAGGGGCAG
AGGGAATTTCCGGTGGAGCGGTGAAATGCATTGAGATCGGAAAGAACACCAACGGC
GAAAGCACTCTGCTGGGCCGACACTGACACTGAGAGACGAAAGCTAGGGGAGCAA
ATGGGATTAGAGACCCCAGTAGTCCTAGCCGTAAACGATGGATACTAGGTGCTGTG
CGACTCGACCCGTGCAGTGCTGTAGCTAACGCGTTAAGTATCCCGCCTGGGGAGTAC
GTTCGCAAGAATGAAACTCAAAGGAATTGACGGGGGCCCGCACAAGCGGTGGAGCA
TGTGGTTTAATTCGATGCAAAGCGAAGAACCTTACCAGGGCTTGACATGCCGCGAAT
CCTCTTGAAAGAGAGGGGTGCCCTCGGGAACGCGGACACAGGTGGTGCATGGCTGT
CGTCAGCTCGTGCCGTAAGGTGTTGGGTTAAGTCTCGCAACGAGCGCAACCCTCGTG
TTTAGTTGCCACTATGAGTTTGGAACCCTGAACAGACCGCCGGTGTTAAGCCGGAGG
AAGGAGAGGATGAGGCCAAGTCATCATGCCCCTTATGCCCTGGGCGACACACGTGC
TACAATGGGCGGGACAAAGGGTCGCGATCTCGCGAGGGTGAGCTAACTCCAAAAAC
CCGTCCTCAGTTCGGATTGCAGGCTGCAACTCGCCTGCATGAAGCAGGAATCGCTAG
TAATCGCCGGTCAGCCATACGGCGGTGAATCCGTTCCCGGGCCTTGTACACACCGCC
CGTCACACTATAGGAGCTGGCCAGGTTTGAAGTCATTACCCTTAACCGTAAGGAGGG
GGATGCCTAAGGCTAGGCTTGCGACTGGAGTGAAGTCGTAACAAGGTAGCCGTACT
GGAAGGTGCGGCTGGATCACCTCCTTT
>P014_WA09_k90.12158 [Polyamia delongi, 16S (partial)]
GGCGGACGGGTGAGTAACGCGTAAGAACCTGCCCTTGGGAGGGGAACAACAGCTGG
AAACGGCTGCTAATACCCCGTAGGCTGAGGAGCAAAAGGAGGAATCCGCCCGAGG
AGGGGCTCGCGTCTGATTAGCTAGTTGGTGAGGCAATAGCTTACCAAGGCGATGATC
AGTAGCTGGTCCGAGAGGATGATCAGCCACACTGGGACTGAGACACGGCCCAGACT
CCTACGGGAGGCAGCAGTGGGGAATTTTCCGCAATGGGCGAAAGCCTGACGGAGCA
ATGCCGCGTGGAGGTAGAAGGCCCACGGGTCGTGAACTTCTTTTCCCGGAGAAGAA
GCAATGACGGTATCTGGGGAATAAGCATCGGCTAACTCTGTGCCAGCAGCCGCGGT
AATACAGAGGATGCAAGCGTTATCCGGAATGATTGGGCGTAAAGCGTCTGTAGGTG
GCTTTTTAAGTCCGCCGTCAAATCCCAGGGCTCAACCCTGGACAGGCGGTGGAAACT
ACCAAGCTGGAGTACGGTAGGGGCAGAGGGAATTTCCGGTGGAGCGGTGAAATGCG

TAGAGATCGGAAAGAACACCAACGGCGAAAGCACTCTGCTGGGCCGACACTGACAC
TGAGAGACGAAAGCTAGGGGAGCGAATGGGATTAGATACCCCAGTAGTCCTAGCCG
TAAACGATGGATACTAGGCGCTGTGCGTATCGACCCGTGCAGTGCTGTAGCTAACGC
GTTAAGTATCCCGCCTGGGGAGTACGTTCGCAAGAATGAAACTCAAAGGAATTGAC
GGGGGCCCGCACAAGCGGTGGAGCATGTGGTTTAATTCGATGCAAAGCGAAGAACC
TTACCAGGGCTTGACATGCCGCGAATCCTCTTGAAAGAGAGGGGTGCCTTCGGGAA
CGCGGACACAGGTGGTGCATGGCTGTCGTCAGCTCGTGCCGTAAGGTGTTGGGTTAA
GTCCCGCAACGAGCGCAACCCTCGTGTTTAGTTGCCACCGTTGAGTTTGGAACCCTG
AGCAGACTGCCGGTGATAAGCCGGAGGAAGGTGAGGATGACGTCAAGTCATCATGC
CCCTTATGCCCTGGGCGACACACGTGCTACAATGGCCGGGACAAAGGGTCGCGATC
CCGCGAGGGTGAGCTAACCCCAAAAACCCGTCCTCAGTTCGGATTGTAGGCTGCAA
CTCGCCTGCATGAAGCCGGAATCGCTAGTAATCGCCGGTCAGCCATACGGCGGTGA
ATTCGTTCCCGGGCCTTGTACACACCGCCCGTCACACTATGGGAGCTGGCCATGCCC
GAAGTCGTTACCTTAACCACAAGGAGGGGGATGCCGAAGGCAGGGCTAGTGACTGG
AGTGAAGTCGTAACAAGGTAGCCGTACTGGAAGGTGCG
>P008_WA11_k90.4854 [Drabescini New Genus ML6 n. sp. 1, 16S]
TCTCATGGAGAGTTCGATCCTGGCTCAGGATGAACGCTGGCGGCATGCTTAACACAT
GCAAGTCGGACGGGAAGTGGTGTTTCCAGTGGCGGACGGGTGAGTAACGCGTAAGA
ACCTGCCCTTGGGAGGGGAACAACAGCTGGAAACGGCTGCTAATACCCCGTAGGCT
GAGGAGCAAAAGGAGGAATCCGCCCGAGGAGGGGCTCGCGTCTGATTAGCTAGTTG
GTGAGGCAATAGCTTACCAAGGCGATGATCAGTAGCTGGTCCGAGAGGATGATCAG
CCACACTGGGACTGAGACACGGCCCAGACTCCTACGGGAGGCAGCAGTGGGGAATT
TTCCGCAATGGGCGAAAGCCTGACGGAGCAATGCCGCGTGGAGGTAGAAGGCCCAC
GGGTCGTGAACTTCTTTTCCCGGAGAAGAAGCAATGACGGTATCTGGGGAATAAGC
ATCGGCTAACTCTGTGCCAGCAGCCGCGGTAATACAGAGGATGCAAGCGTTATCCG
GAATGATTGGGCGTAAAGCGTCTGTAGGTGGCTTTTTAAGTCCGCCGTCAAATCCCA
GGGCTCAACCCTGGACAGGCGGTGGAAACTACCAAGCTGGAGTACGGTAGGGGCAG
AGGGAATTTCCGGTGGAGCGGTGAAATGCGTAGAGATCGGAAAGAACACCAACGGC
GAAAGCACTCTGCTGGGCCGACACTGACACTGAGAGACGAAAGCTAGGGGAGCGA
ATGGGATTAGATACCCCAGTAGTCCTAGCCGTAAACGATGGATACTAGGCGCTGTGC
GTATCGACCCGTGCAGTGCTGTAGCTAACGCGTTAAGTATCCCGCCTGGGGAGTACG
TTCGCAAGAATGAAACTCAAAGGAATTGACGGGGGCCCGCACAAGCGGTGGAGCAT
GTGGTTTAATTCGATGCAAAGCGAAGAACCTTACCAGGGCTTGACATGCCGCGAATC
CTCTTGAAAGAGAGGGGTGCCTTCGGGAACGCGGACACAGGTGGTGCATGGCTGTC
GTCAGCTCGTGCCGTAAGGTGTTGGGTTAAGTCCCGCAACGAGCGCAACCCTCGTGT
TTAGTTGCCATCATTGAGTTTGGAACCCTGAACAGACTGCCGGTGATAAGCCGGAGG
AAGGTGAGGATGACGTCAAGTCATCATGCCCCTTATGCCCTGGGCGACACACGTGCT
ACAATGGCCGGGACAAAGGGTCGCGATCCCGCGAGGGTGAGCTAACTCCAAAAACC
CGTCCTCAGTTCGGATTGCAGGCTGCAACTCGCCTGCATGAAGCCGGAATCGCTAGT
AATCGCCGGTCAGCCATACGGCGGTGAATTCGTTCCCGGGCCTTGTACACACCGCCC
GTCACACTATGGGAGCTGGCCATGCCCGAAGTCGTTACCTTAACCGCAAGGAGGGG
GATGCCGAAGGCAGGGCTAGTGACTGGAGTGAAGTCGTAACAAGGTAGCCGTACTG
GAAGGTGCGGCTGGATCACCTCCTTT
>P021_WG09_k29.6675 [Paradorydium n. sp., matK (partial)]
AGAATACTTTGTTTTTGACTGTATCGCACTGTGTATCATTGGAAAATTTCCCAACCTT
CTACCTTCATTTCAAATATACTATTAAATGGAGGAAATCCAAAGCTATTTACAACTT

GATAGATCTCAACAGCCCGGCTTTCTATATCCACTTATTTTTCAGGAGTATATTTACG
GGCTTGCTCATGATTATATTTTAAATCGATCTCGCTTGTTGGGAAATCCGGGTT
>P019_WF02_k50.2776 [Placotettix taeniatifrons, matK (partial)]
TATCAAATGGAGGAAATCCAAAGATATTTACAGCTTGATAGATCTCAACAACACGG
CTTTCTATATCCACTTATCTTTCAGGAGTATATTTATGCACTTGCTCATGATCATAGT
TTAAACCGATCTATTTTGTTGGAAAATCCAGGTTATGACAATCAATTCAGTTTCCTAA
TTGTGAAACG
>P011_WH08_k90.15381 [Hecalus major, 16S (partial)]
TCTCATGGAGAGTTCGATCCTGGCTCAGGATGAACGCTGGCGGCATGCTTAACACAT
GCAAGTCGGACGGGAAGTGGTGTTTCCAGTGGCGGACGGGTGAGTAACGCGTAAGA
ACCTGCCCTTGGGAGGGGAACAACAGCTGGAAACGGCTGCTAATACCCCGTAGGCT
GAGGAGCAAAAGGAGGAATCCGCCCGAGGAGGGGCTCGCGTCTGATTAGCTAGTTG
GTGAGGCAATAGCTTACCAAGGCGATGATCAGTAGCTGGTCCGAGAGGATGATCAG
CCACACTGGGACTGAGACACGGCCCAGACTCCTACGGGAGGCAGCAGTGGGGAATT
TTCCGCAATGGGCGAAAGCCTGACGGAGCAATGCCGCGTGGAGGTAGAAGGCCCAC
GGGTCGTGAACTTCTTTTCCCGGAGAAGAAGCAATGACGGTATCTGGGGAATAAGC
ATCGGCTAACTCTGTGCCAGCAGCCGCGGTAATACAGAGGATGCAAGCGTTATCCG
GAATGATTGGGCGTAAAGCGTCTGTAGGTGGCTTTTTAAGTCCGCCGTCAAATCCCA
GGGCTCAACCCTGGACAGGCGGTGGAAACTACCAAGCTGGAGTACGGTAGGGGCAG
AGGGAATTTCCGGTGGAGCGGTGAAATGCGTAGAGATCGGAAAGAACACCAACGGC
GAAAGCACTCTGCTGGGCCGACACTGACACTGAGAGACGAAAGCTAGGGGAGCGA
ATGGGATTAGATACCCCAGTAGTCCTAGCCGTAAACGATGGATACTAGGCGCTGTGC
GTATCGACCCGTGCAGTGCTGTAGCTAACGCGTTAAGTATCCCGCCTGGGGAGTACG
TTCGCAAGAATGAAACTCAAAGGAATTGACGGGGGCCCGCACAAGCGGTGGA
>P021_WB02_k29.30957 [Pinopona sp., matK (partial)]
AACTATATCGCACTATGTATCATTTGATAATCCCAAAATCTTCTACCTTTGGTTCAAA
TCTAATATCAAATGGAGGAAATCCAAAGATATTTACAGCTTGATAGATCTCAACAAC
ACGGCTTTCTATATCCACTTATCTTTCAGGAGTATATTTAT
>P019_WA11_k90.3129 [Balclutha chloe, 16S (partial)]
TCTCATGGAGAGTTCGATCCTGGCTCAGGATGAACGCTGGCGGCATGCTTAACACAT
GCAAGTCGGACGGGAAGTGGTGTTTCCAGTGGCGGACGGGTGAGTAACGCGTAAGA
ACCTGCCCTTGGGAGGGGAACAACAGCTGGAAACGGCTGCTAATACCCCGTAGGCT
GAGGAGCAAAAGGAGGAATCCGCCCGAGGAGGGGCTCGCGTCTGATTAGCTAGTTG
GTGAGGCAATAGCTTACCAAGGCGATGATCAGTAGCTGGTCCGAGAGGATGATCAG
CCACACTGGGACTGAGACACGGCCCAGACTCCTACGGGAGGCAGCAGTGGGGAATT
TTCCGCAATGGGCGAAAGCCTGACGGAGCAATGCCGCGTGGAGGTAGAAGGCCCAC
GGGTCGTGAACTTCTTTTCCCGGAGAAGAAGCAATGACGGTATCTGGGGAATAAGC
ATCGGCTAACTCTGTGCCAGCAGCCGCGGTAATACAGAGGATGCAAGCGTTATCCG
GAATGATTGGGCGTAAAGCGTCTGTAGGTGGCTTTTTAAGTCCGCCGTCAAATCCCA
GGGCTCAACCCTGGACAGGCGGTGGAAACTACCAAGCTGGAGTACGGTAGGGGCAG
AGGGAATTTCCGGTGGAGCGGTGAAATGCGTAGAGATCGGAAAGAACACCAACGGC
GAAAGCACTCTGCTGGGCCGACACTGACACTGAGAGACGAAAGCTAGGGGAGCGA
ATGGGATTAGATACCCCAGTAGTCCTAGCCGTAAACGATGGATACTAGGCGCTGTGC
GTATCGACCCGTGCAGTGCTGTAGCTAACGCGTTAAGTATCCCGCCTGGGGAGTACG
TTCGCAAGAATGAAACTCAAAGGAATTGACGGGGGCCCGCACAAGCGGTGGAGCAT
GTGGTTTAATTCGATGCAAAGCGAAGAACCTTACCAGGGCTTGACATGCCGCGAATC

CTCTTGAAAGAGAGGGGTGCCTTCGGGAACGCGGACACAGGTGGTGCATGGCTGTC
GTCAGCTCGTGCCGTAAGGTGTTGGGTTAAGTCCCGCAACGAGCGCAACCCTCGTGC
TTAGTTGCCACCGTCGAGTTTGGAACCCTGAGCAGACTGCCGGTGATAAGCCGGAG
GAAGGTGAGGATGACGTCAAGTCATCATGCCCCTTATGCCCTGGGCGACACACGTG
CTAC
>P019_WA11_k50.15260 [Balclutha chloe, matK (partial)]
GCGCTGTGTATCATTCGAAAATTCCCAAATCTTATGCCTTCATTTCAAATATACTATT
AAATGGAGGAAATCCAAGGCTATTTACAGCTTGATAGATCTCAACAGCCCGGCTTTC
TATATCCACTTATCTTTCAGGAGTATATTTACGGACTTGCTCATGATTATAGTTTAAA
TCGATCTCGCTTGTTGAAAAATCCGGGTTATGACAATAAATACAGTTTACTACTTGT
GAAACGTTTAATTACTCGAATGTATGGA
>P015_WE08_k90.14268 [Hishimonus fuscomaculatus, 16S]
TCTCATGGAGAGTTCGATCCTGGCTCAGGATGAACGCTGGCGGCATGCTTAACACAT
GCAAGTCGGACGGGAAGTGGTGTTTCCAGTGGCGGACGGGTGAGTAACGCGTAAGA
ACCTGCCCTTGGGAGGGGAACAACAGCTGGAAACGGCTGCTAATACCCCGTAGGCT
GAGGAGCAAAAGGAGGAATCCGCCCGAGGAGGGGCTCGCGTCTGATTAGCTAGTTG
GTGAGGCAATAGCTTACCAAGGCGATGATCAGTAGCTGGTCCGAGAGGATGATCAG
CCACACTGGGACTGAGACACGGCCCAGACTCCTACGGGAGGCAGCAGTGGGGAATT
TTCCGCAATGGGCGAAAGCCTGACGGAGCAATGCCGCGTGGAGGTAGAAGGCCCAC
GGGTCGTGAACTTCTTTTCCCGGAGAAGAAGCAATGACGGTATCTGGGGAATAAGC
ATCGGCTAACTCTGTGCCAGCAGCCGCGGTAATACAGAGGATGCAAGCGTTATCCG
GAATGATTGGGCGTAAAGCGTCTGTAGGTGGCTTTTTAAGTCCGCCGTCAAATCCCA
GGGCTCAACCCTGGACAGGCGGTGGAAACTACCAAGCTGGAGTACGGTAGGGGCAG
AGGGAATTTCCGGTGGAGCGGTGAAATGCGTAGAGATCGGAAAGAACACCAACGGC
GAAAGCACTCTGCTGGGCCGACACTGACACTGAGAGGCGAAAGCTAGGGGAGCGA
ATGGGATTAGATACCCCAGTAGTCCTAGCCGTAAACGATGGATACTAGGCGCTGTGC
GTATCGACCCGTGCAGTGCTGTAGCTAACGCGTTAAGTATCCCGCCTGGGGAGTACG
TTCGCAAGAATGAAACTCAAAGGAATTGACGGGGGCCCGCACAAGCGGTGGAGCAT
GTGGTTTAATTCGATGCAAAGCGAAGAACCTTACCAGGGCTTGACATGCCGCGAATC
CTCTTGAAAGAGAGGTGTGCCTTCGGGAACGCGGACACAGGTGGTGCATGGCTGTC
GTCAGCTCGTGCCGTAAGGTGTTGGGTTAAGTCCCGCAACGAGCGCAACCCTCGTGT
TTAGTTGCCACCGTTGAGTTTGGAACCCTGAACAGACTGCCGGTGATAAGCCGGAGG
AAGGTGAGGATGACGTCAAGTCATCATGCCCCTTATGCCCTGGGCGACACACGTGCT
ACAATGGCCGGGACAAAGGGTCGCGATCCCGCGAGGGTGAGCTAACTCCAAAAACC
CGTCCTCAGTTCGGATTGCAGGCTGCAACTCGCCTGCATGAAGCCGGAATCGCTAGT
AATCGCCGGTCAGCCATACGGCGGTGAATTCGTTCCCGGGCCTTGTACACACCGCCC
GTCACACTATGGGAGCTGGCCATGCCCGAAGTCGTTACCTTAACCGCAAGGAGGGG
GATGCCGAAGGCAGGGCTAGTGACTGGAGTGAAGTCGTAACAAGGTAGCCGTACTG
GAAGGTGCGGCTGGATCACCTCCTTT
>P021_WE04_k29.22888 [Neoaliturus alboflavovittatus, matK (partial)]
ATCCCAAAATCTTCTACCTTTGGTTCAAATCTAATATCAAATGGAGGAAATCCAAAG
ATATTTACAGCTTGATAGATCTCAACAACACGGCTTTCTATATCCACTTATCTTTCAG
GAGTATATTTATGCACTTGCTCATGATCATAGTTTAAACCGATCTATTTTGTTGGAAA
ATCCAGGTTATGACAATCAATTCAGTTTCCTAATTGTGAAAC
>P021_WD01_k90.2091 [Neoaliturus tenellus, matK (partial)]

TATCATTTGATAATCCCAAAATCTTCTACCTTTGGTTCAAATCTAATATCAAATGGAG
GAAATCAAAAGATATTTACAGCTTGATAGATCTCAACAACACGGCTTTCTATATCCA
CTTATCTTTCAGGAGTATATTTATGCACTTGCTCATGATCATAGTTTAAACCGATCTA
TTTTGTTGGAAAATCCAGGTTATGACAATCAATTCAGTTTCC
>P021_WD03_k50.2952 [Neoalituris tenellus, matK (partial)]
TTTGGTTTAACTATATCGCACTATGTATCATTTGATAATCCCAAAATCTTCTACCTTT
GGTTCAAATCTAATATCAAATGGAGGAAATCCAAAGATATTTACAGCTTGATAGATC
TCAACAACACGGCTTTCTATATCCACTTATCTTTCAGGAGTATATTTATGCACTTGCT
CATGATCATAGTTTAAACCGATCTATTTTGTTGGAAAATCCAGGTTATGACAATCAA
TTCAGTTTCCTAATTGTGAAAC
>P021_WD04_k29.44833 [Neoalituris tenellus, matK (partial)]
AAAAAAAAAAGAGAGGATAGAGAATCTGTTGATAAGTTTACCCCCGTCTCCGAGGT
ATTCTATTCTTACTAGAATACTTTGGTTTAACTATATCGCACTATGTATCATTTGATA
ATCCCAAAATCTTCTACCTTTGGTTCAAATCTAATATCAAATGGAGGAAATCCAAAG
ATATTTACAGCTTGATAGATCTCAACAACACGGCTTTCTATATCC
>P021_WD04_k50.22385 [Neoalituris tenellus, matK (partial)]
TCTTGTTATCCTATAACATAGACTAAAAATGAAAAAAAAAAAGAGAGGATAGAGAA
TCTGTTGATAAGTTTACCCCCGTCTCCGAGGTATTCTATTCTTACTAGAATACTTTGG
TTTAACTATATCGCACTATGTATCATTTGATAATCCCAAAATCTTCTACCTTTGGTTC
AAATCTAATATCAAATGGAGGAAATCCAAAGATATTTACAGCTTGATAGATCTCAAC
AACACGGCTTTCTATATCCACTTATCTTTCAGGAGTATAT
>P021_WD04_k50.2986 [Neoalituris tenellus, matK (partial)]
GATCTCAACAACACGGCTTTCTATATCCACTTATCTTTCAGGAGTATATTTATGCACT
TGCTCATGATCATAGTTTAAACCGATCTATTTTGTTGGAAAATCCAGGTCATGACAA
TCAATTCAGTTTCCTAATTGTGAAACGTTTAATTACTCGAATGTATCAACAAAATCAT
TTTATTATTTTTGCTAATGATTCTAATCAAAATCCATTTTTTGGTCGCAACAAGAATT
TATATTCTCAAACGATATCAGAAGGATTTGCATTTATTGTGGAAATTCCATTTTATAT
ACAATTAATATCTTCTCAAGCGGGGAAAGGTATATTAAAATCTTATAATTTAGGATC
AATTCATTCACTATTTCCTTTCTTAGAGAACAATTTGTCACATTTAAATTCTGTGTTA
AA
>P021_WD04_k90.78 [Neoalituris tenellus, matK (partial)]
AAAAAAAAAAGAGAGGATAGAGAATCTGTTGATAAGTTTACCCCCGTCTCCGAGGT
ATTCTATTCTTACTAGAATACTTTGGTTTAACTATATCGCACTATGTATCATTTGATA
ATCCCAAAATCTTCTACCTTTGGTTCAAATCTAATATCAAATGGAGGAAATCCAAAG
ATATTTACAGCTTGATAGATCTCAACAACACGGCTTTCTATATCCACTTATCTTTCAG
GAGTATATTTATGCACTTGCTCATGATCATAGTTTAAACCGATCTATTTTGTTGGAAA
ATCCAGGTCATGACAATCAATTCAGTTTCCTAATTGTGAAACGTTTAATTACTCGAA
TGTATCAACAAAATCATTTTATTATTTTTGCTAATGATTCTAATCAAAATCCATTTTT
TGGTCGCAACAAGAATTTATATTCTCAAACGATATCAGAAGGATTTGCATTTATTGT
GGAAATTCCATTTTATATACAATTAATATCTTC
>P021_WE10_k90.3344 [Neoalituris tenellus, matK (partial)]
ATCCTCTTTGCTACCCCAAGCATATCCTAAAAATGGAAAAGACGAAGATAGAGAAT
CTGTTGATAAGTTTCTACTCGCCTCCGAGGTATCTATTCTTAAGAGAATACTTTGTTT
TTGACTGTATCGCACTGTGTATCATTGGAAAATTTCCCAATCTTCTAACTTCATTTCA
AATATACTATTAAATGGAGGAAATCCAAAGCTATTTACAACTTGATAGATCTCAACA
GCCCGGCTTTCTATATCCACTTATTTTTCAGGAGTATATTTACGGGCTTGCTCATGAT

TATATTTTAAATCGATCTCGCTTGTTGGGAAATCCGGGTTATGACAATAAATACAGT
TTACTATTTGTGAAACGTTTAATTACTCGAATGTATGGACAAAATCATTTTATTATTT
TTGCTAATGATTCTAATAAAAATTACTTTTTTGGTC
>P021_WE01_k29.19529 [Neoaliturus fenestratus, matK (partial)]
ATAATCTCAAAATCTTCTACCTTTGGTTCAAATCTAATATCAAATGGAGGAAATCAA
AAGATATTTGCAGCTTGATAGATCTCAACAACACGGCTTTCTATATCCACTTATCTTT
CAGGAGTATATTTATGCACTTGCTCATGATCATAGTTTAAACCGAT
>P021_WE02_k29.6966 [Neoaliturus fenestratus, matK (partial)]
AGGAAATCCAAAGATATTTACAGCTTGATAGATCTCAACAACACGGCTTTCTATATC
CACTTATCTTTCAGGAGTATATTTATGCACTTGCTCATGATCATAGTTTAAACCGATA
ACCCGGCATTGGCCGCGCTCACCGGGGTCTCAACGCCCGCTGAACCCGCCCGTTTCA
ATGCGGCAGAATTGGCCGCCATAGAAGCCAGACGAGAAGCGAAAG
>P021_WE05_k29.12485 [Neoaliturus inscriptus, rbcL (partial)]
CGTTACAAAGGACGATGCTACCACATCGATGCCGTTCCTGGAGAAGACAATCAATA
TATTTGTTATGTAGCTTACCCCTTAGACCTTTTTGAAGAAGGTTCTGTTACTAATATG
TTTACTTCCATCGTGGGCAATGTATTTGGGTTCAAAGCCCTGCGTG
>P014_WE06_k90.1540 [Nesophrosyne n. sp., 16S (partial)]
CGGTGAAATGCGTAGAGATCGGAAAGAACACCAACGGCGAAAGCACTCTGCTGGGC
CGACACTGACACTGAGAGGCGAAAGCTAGGGGAGCGAATGGGATTAGATACCCCAG
TAGTCCTAGCCGTAAACGATGGATACTAGGCGCTGTGCGTATCGACCCGTGCAGTGC
TGTAGCTAACGCGTTAAGTATCCCGCCTGGGGAGTACGTTCGCAAGAATGAAACTCA
AAGGAATTGACGGGGGCCCGCACAAGCGGTGGAGCATGTGGTTTAATTCGATGCAA
AGCGAAGAACCTTACCAGGGCTTGACATGCCGCGAATCCTCTTGAAAGAGAGGTGT
GCCTTCGGGAACGCGGACACAGGTGGTGCATGGCTGTCGTCAGCTCGTGCCGTAAG
GTGTTGGGTTAAGTCCCGCAACGAGCGCAACCCTCGTGTTTAGTTGCCACCGTTGAG
TTTGGAACCCTGAACAGACTGCCGGTGATAAGCCGGAGGAAGGTGAGGATGACGTC
AAGTCATCATGCCCCTTATGCCCTGGGCGACACACGTGCTACAATGGCCGGGACAA
AGGGTCGCGATCCCGCGAGGGTGAGCTAACTCCAAAAACCCGTCCTCAGTTCGGAT
TGCAGGCTGCAACTCGCCTGCATGAAGCCGGAATCGCTAGTAATCGCCGGTCAGCC
ATACGGCGGTGAATTCGTTCCCGGGCCTTGTACACACCGCCCGTCACACTATGGGAG
CTGGCCATGCCCGAAGTCGTTACCTTAACCGCAAGGAGGGGGATGCCGAAGGCAGG
GCTAGTGACTGGAGTGAAGTCGTAACAAGGTAGCCGTACTGGAAGGTGCGGCTGGA
TCACCTCCTTT
>P004_WA04_k90.1006 [Aflexia rubranura, 16S]
TCTCATGGAGAGTTTGATCCTGGCTCAGGATGAACGCTGGCGGCATGCTTAACACAT
GCAAGTCGGACGGGAAGTGGTGTTTCCAGTGGCGGACGGGTGAGTAACGCGTAAGA
ACCTGCCCTTGGGAGGGGAACAACAGCTGGAAACGGCTGCTAATACCCCATAGGCT
GAGGAGCAAAAGGAGGAATCCGCCCAAGGAGGGGCTCGCGTCTGATTAGTTAGTTG
GTGAGGCAATGGCTTACCAAGGCGACGATCAGTAGCTGGTCCGAGAGGATGATCAG
CCACACTGGGACTGAGACACGGCCCAGACTCCTACGGGAGGCAGCAGTGGGGAATT
TTCCGCAATGGGCGAAAGCCTGACGGAGCAATGCCGCGTGAAGGCAGAAGGCCCAC
GGGTCATGAACTTCTTTTCTCGGAGAAGAAAAAATGACGGTATCTGAGGAATAAGC
ATCGGCTAACTCTGTGCCAGCAGCCGCGGTAAGACAGAGGATGCAAGCGTTATCCG
GAATGATTGGGCGTAAAGCGTCTGTAGGTGGCTTTTCAAGTCCGCCGTCAAATCCCC
GGGCTCAACCCTGGACAGGCAGTGGAAACTACCAAGCTGGAGTACGGTAGGGGCAG
AGGGAATTTCCGGTGGAGCGGTGAAATGCGTTGAGATCGGAAAGAACACCAACGGC

GAAAGCACTCTGCTGGGCCGACACTGACACTGAGAGACGAAAGCTAGGGGAGCAA
ATGGGATTAGATACCCCAGTAGTCCTAGCCGTAAACGATGGATACTAAGTGCTGTGC
GTATCGACCCGCGCAGTGCTGTAGCTAACGCGTTAAGTATCCCGCCTGGGGAGTACG
TTCGCAAGAATGAAACTCAAAGGAATTGACGGGGGCCCGCACAAGCGGTGGAGCAT
GTGGTTCAATTCGATGCAAAGCGAAGAACCTTACCAGGGCTTGACATGCCGTGAATC
CTCCCGAAAGAGAGGAGTGCCTTCGGGAACGCGGACACAGGTGGTGCATGGCTGTC
GTCAGCTCGTGCCGTAAGGTGTTGGGTTAAGTCCCGCAACGAGCGCAACCCTCGTGT
TTAGTTGCCAGCATTTAGTTTGGAACCCTGAACAGACTGCCGGTGATAAGCCGGAGG
AAGGTGAGGATGACGTCAAGTCATCATGCCCCTTACGCCCTGGGCGACACACGTGC
TACAATGACCGGGACAAAGGGTCGCGACCCCGCGAGGGCAAGCTAACCTCAAAAAC
CCGGCCTCAGTTCGGATTGCAGGCTGCAACTCGCCTGCATGAAGCCGGAATCGCTAG
TAATCGCCGGTCAGCCATACGGCGGTGAATCCGTTCCCGGGCCTTGTACACACCGCC
CGTCACACTATGGGAGCTGGCCATGCCCCAAGTCGTTACCTTAACCGCAAGGAGGG
GGATGCCGAAGGCTGGGCTAGTGACTGGAGTGAAGTCGTAACAAGGTAGCCGTACT
GGAAGGTGCGGCTGGATCACCTCCTTT
>P021_WA10_k50.10528 [Myittana n. sp. T1, matK (partial)]
AATATTACTCATTTCAAATATACTATTAAATGGAGGAAATCCAAAGCTATTTACAAC
TTGATAGATTTCAACAGCCCGGCTTTCTATATCCACTTATCTTTCAGGAGTATATTTA
CGGGCTTGTTCATGATTTAGTTTAAATCGATCTCGCTTGTTGGAAAATCCGGGTTAT
GGCAATAAATACAGTTTACTACTTGTGAAACGTTTAATTA
>P006_WE02_k90.9091 [Paralimnini New Genus AU2 n. sp. 1, 16S (partial)]
CGCCGTCAAATCCCAGGGCTCAACCCTGGACAGGCGGTGGAAACTACCAAGCTGGA
GTACGGTAGGGGCAGAGGGAATTTCCGGTGGAGCGGTGAAATGCGTAGAGATCGGA
AAGAACACCAACGGCGAAAGCACTCTGCTGGGCCGACACTGACACTGAGAGACGA
AAGCTAGGGGAGCGAATGGGATTAGATACCCCAGTAGTCCTAGCCGTAAACGATGG
ATACTAGGCGCTGTGCGTATCGACCCGTGCAGTGCTGTAGCTAACGCGTTAAGTATC
CCGCCTGGGGAGTACGTTCGCAAGAATGAAACTCAAAGGAATTGACGGGGGCCCGC
ACAAGCGGTGGAGCATGTGGTTTAATTCGATGCAAAGCGAAGAACCTTACCAGGGA
TTGACATGCCGCGAATCCTCTTGAAAGAGAGGGGTGCCTTCGGGAACGCGGACACA
GGTGGTGCATGGCTGTCGTCAGCTCGTGCCGTAAGGTGTTGGGTTAAGTCCCGCAAC
GAGCGCAACCCTCGTGTTTAGTTGCCACCGTTGAGTTTGGAACCCTGAGCAGACTGC
CGGTGATAAGCCGGAGGAAGGTGAGGATGACGTCAAGTCATCATGCCCCTTATGCC
CTGGGCGACACACGTGCTACAATGGCCGGGACAAAGGGTCGCGATCCCGCGAGGGT
GAGCTAACTCCAAAAACCCGTCCTCAGTTCGGATTGCAGGCTGCAACTCGCCTGCAT
GAAGCCGGAATCGCTAGTAATCGCCGGTCAGCCATACGGCGGTGAATTCGTTCCCG
GGCCTTGTACACACCGCCCGTCACACTATGGGAGCTGGCCATGCCCGAAGTCGTTAC
CTTAACCGCAAGGAGGGGGATGCCGAAGGCAGGGCTAGTGACTGGAGTGAAGTCGT
AACAAGGTAGC
>P008_WH01_k90.2022 [Paralimnini New Genus T2 n. sp. 2, 16S]
TCTCATGGAGAGTTCGATCCTGGCTCAGGATGAACGCTGGCGGCATGCCTTACACAT
GCAAGTCGGACGGGAAGTGGTGTTTCCAGTGGCGGACGGGTGAGTAACGCGTAAGA
ACCTACCCTTGGGAGGGGAACAACAGCTGGAAACGGCTGCTAATACCCCGTAGGCT
GAGGAGCAAAAGGAGGAATCCGCCCGAGGAGGGGCTCGCGTCTGATTAGCTAGTTG
GTGAGGCAATAGCTTACCAAGGCGATGATCAGTAGCTGGTCCGAGAGGATGATCAG
CCACACTGGGACTGAGACACGGCCCAGACTCCTACGGGAGGCAGCAGTGGGGAATT
TTCCGCAATGGGCGAAAGCCTGACGGAGCAATGCCGCGTGAAGGTAGAAGGCCTAC

GGGTCATGAACTTCTTTTCCCGGAGAAGAAGCAATGACGGTATCCGGGGAATAAGC
ATCGGCTAACTCTGTGCCAGCAGCCGCGGTAAGACAGAGGATGCAAGCGTTATCCG
GAATGATTGGGCGTAAAGCGTCTGTAGGTGGCTTTTTAAGTTCGCCGTCAAATCCCA
GGGCTCAACCCTGGACAGGCGGTGGAAACTACCAAGCTGGAGTACGGTAGGGGCAG
AGGGAATTTCCGGTGGAGCGGTGAAATGCGTAGAGATCGGAAAGAACACCAACGGC
GAAAGCACTCTGCTGGGCCGACACTGACACTGAGAGACGAAAGCTAGGGGAGCGA
ATGGGATTAGATACCCCAGTAGTCCTAGCCGTAAACGATGGATACTAGGCGCTGTGC
GTATCGACCCGTGCAATGCTGTAGCTAACGCGTTAAGTATCCCGCCTGGGGAGTACG
TTCGCAAGAATGAAACTCAAAGGAATTGACGGGGGCCCGCACAAGCGGTGGAGCAT
GTGGTTTAATTCGATGCAAAGCGAAGAACCTTACCAGGGCTTGACATGCCGCGAATC
CTCTTGAAAGAGAGGGGTGCCTTCGGGAACGCGGACACAGGTGGTGCATGGCTGTC
GTCAGCTCGTGCCGTAAGGTGTTGGGTTAAGTCCCGCAACGAGCGCAACCCTCGTGT
TTAGTTGCCAACATTTAGTTTGGAACCCTGAGCAGACTGCCGGTGATAAGCCGGAGG
AAGGTGAGGATGACGTCAAGTCATCATGCCCCTTATGCCCTGGGCGACACACGTGCT
ACAATGGACGGGACAAAGGATCGCGATCCCGCGAGGGTGAGCTAACTCCAAAAACC
CGTCCTCAGTTCGGATTGTAGGCTGCAACTCGCCTGCATGAAGCCGGAATCGCTAGT
AATCGCCGGTCAGCCATACGGCGGTGAATTCGTTCCCGGGCCTTGTACACACCGCCC
GTCACACTATGGGAGCTGGCCATGCCCGAAGTCGTTACCTTAACCGCAAGGAGGGG
GATGCCGAAGGCAGGGCTAGTGACTGGAGTGAAGTCGTAACAAGGTAGCCGTACTG
GAAGGTGCGGCTGGATCACCTCCTTT
>P007_WH01_k90.2668 [Pravistylus trunculidiscus, 16S (partial)]
TCTCATGGAGAGTTCGATCCTGGCTCAGGATGAACGCTGGCGGCATGCCTTACACAT
GCAAGTCGGACGGGAAGTGGTGTTTCCAGTGGCGGACGGGTGAGTAACGCGTAAGA
ACCTACCCTTGGGAGGGGAACAACAGCTGGAAACGGCTGCTAATACCCCGTAGGCT
GAGGAGCAAAAGGAGGAATCCGCCCGAGGAGGGGCTCGCGTCTGATTAGCTAGTTG
GTGAGGCAATAGCTTACCAAGGCGATGATCAGTAGCTGGTCCGAGAGGATGATCAG
CCACACTGGGACTGAGACACGGCCCAGACTCCTACGGGAGGCAGCAGTGGGGAATT
TTCCGCAATGGGCGAAAGCCTGACGGAGCAATGCCGCGTGAAGGTAGAAGGCCTAC
GGGTCATGAACTTCTTTTCCCGGAGAAGAAGCAATGACGGTATCCGGGGAATAAGC
ATCGGCTAACTCTGTGCCAGCAGCCGCGGTAAGACAGAGGATGCAAGCGTTATCCG
GAATGATTGGGCGTAAAGCGTCTGTAGGTGGCTTTTTAAGTTCGCCGTCAAATCCCA
GGGCTCAACCCTGGACAGGCGGTGGAAACTACCAAGCTGGAGTACGGTAGGGGCAG
AGGGAATTTCCGGTGGAGCGGTGAAATGCGTAGAGATCGGAAAGAACACCAACGGC
GAAAGCACTCTGCTGGGCCGACACTGACACTGAGAGACGAAAGCTAGGGGAGCGA
ATGGGATTAGATACCCCAGTAGTCCTAGCCGTAAACGATGGATACTAGGCGCTGTGC
GTATCGACCCGTGCAATGCTGTAGCTAACGCGTTAAGTATCCCGCCTGGGGAGTACG
TTCGCAAGAATGAAACTCAAAGGAATTGACGGGGGCCCGCACAAGCGGTGGAGCAT
GTGGTTTAATTCGATGCAAAGCGAAGAACCTTACCAGGGCTTGACATGCCGCGAATC
CTCTTGAAAGAGAGGGGTGCCTTCGGGAACGCGGACACAGGTGGTGCATGGCTGTC
GTCAGCTCGTGCCGTAAGGTGTTGGGTTAAGTCCCGCAACGAGCGCAACCCTCGTGT
TTAGTTGCCAACATTTAGTTTGGAACCCTGAGCAGACTGCCGGTGATAAGCCGGAGG
AAGGTGAGGATGACGTCAAGTCATCATGCCCCTTATGCCCTGGGCGACACACGTGCT
ACAATGGACGGGACAAAGGATCGCGATCCCGCGAGGGTGAGCTAACTCCAAAAACC
CGTCCTCAGTTCGGATTGTAGGCTGCAACTCGCCTGCATGAAGCCGGAATCGCTAGT
AATCGCCGGTCAGCCATACGGCGGTGAATTCGTTCCCGGGCCTTGTACACACCGCCC
GTCACACTATGGGAGCTGGCCATGCCCGAAGTCGTTACCTTAACCGCAAGGAGGGG

GATGCCGAAGGCAGGGCTAGTGACTGGAGTGAAGTCGTAACAAGGTAGCCGTACTG
GAAGGTGCGGCTGGATCAC
>P015_WE12_k90.2893 [Sorhoanus pratensis, 16S]
TCTCATGGAGAGTTCGATCCTGGCTCAGGATGAACGCTGGCGGCATGCTTAACACAT
GCAAGTCGGACGGGAAGTGGTGTTTCCAGTGGCGGACGGGTGAGTAACGCGTAAGA
ACCTGCCCTTGGGAGGGGAACAACAGCTGGAAACGGCTGCTAATACCCCGTAGGCT
GAGGAGCAAAAGGAGGAATCCGCCCGAGGAGGGGCTCGCGTCTGATTAGCTAGTTG
GTGAGGCAATAGCTTACCAAGGCGATGATCAGTAGCTGGTCCGAGAGGATGATCAG
CCACACTGGGACTGAGACACGGCCCAGACTCCTACGGGAGGCAGCAGTGGGGAATT
TTCCGCAATGGGCGAAAGCCTGACGGAGCAATGCCGCGTGGAGGTAGAAGGCCCAC
GGGTCGTGAACTTCTTTTCCCGGAGAAGAAGCAATGACGGTATCTGGGGAATAAGC
ATCGGCTAACTCTGTGCCAGCAGCCGCGGTAATACAGAGGATGCAAGCGTTATCCG
GAATGATTGGGCGTAAAGCGTCTGTAGGTGGCTTTTTAAGTCCGCCGTCAAATCCCA
GGGCTCAACCCTGGACAGGCGGTGGAAACTACCAAGCTGGAGTACGGTAGGGGCAG
AGGGAATTTCCGGTGGAGCGGTGAAATGCGTAGAGATCGGAAAGAACACCAACGGC
GAAAGCACTCTGCTGGGCCGACACTGACACTGAGAGGCGAAAGCTAGGGGAGCGA
ATGGGATTAGATACCCCAGTAGTCCTAGCCGTAAACGATGGATACTAGGCGCTGTGC
GTATCGACCCGTGCAGTGCTGTAGCTAACGCGTTAAGTATCCCGCCTGGGGAGTACG
TTCGCAAGAATGAAACTCAAAGGAATTGACGGGGGCCCGCACAAGCGGTGGAGCAT
GTGGTTTAATTCGATGCAAAGCGAAGAACCTTACCAGGGCTTGACATGCCGCGAATC
CTCTTGAAAGAGAGGTGTGCCTTCGGGAACGCGGACACAGGTGGTGCATGGCTGTC
GTCAGCTCGTGCCGTAAGGTGTTGGGTTAAGTCCCGCAACGAGCGCAACCCTCGTGT
TTAGTTGCCACCGTTGAGTTTGGAACCCTGAACAGACTGCCGGTGATAAGCCGGAGG
AAGGTGAGGATGACGTCAAGTCATCATGCCCCTTATGCCCTGGGCGACACACGTGCT
ACAATGGCCGGGACAAAGGGTCGCGATCCCGCGAGGGTGAGCTAACTCCAAAAACC
CGTCCTCAGTTCGGATTGCAGGCTGCAACTCGCCTGCATGAAGCCGGAATCGCTAGT
AATCGCCGGTCAGCCATACGGCGGTGAATTCGTTCCCGGGCCTTGTACACACCGCCC
GTCACACTATGGGAGCTGGCCATGCCCGAAGTCGTTACCTTAACCGCAAGGAGGGG
GATGCCGAAGGCAGGGCTAGTGACTGGAGTGAAGTCGTAACAAGGTAGCCGTACTG
GAAGGTGCGGCTGGATCACCTCCTTT
>P002_WG08_k90.17236 [Turrutus personatus, 16S (partial)]
TCTCATGGAGAGTTCGATCCTGGCTCAGGATGAACGCTGGCGGCATGCTTAACACAT
GCAAGTCGGACGGGAAGTGGTGTTTCCAGTGGCGGACGGGTGAGTAACGCGTAAGA
ACCTGCCCTTGGGAGGGGAACAACAGCTGGAAACGGCTGCTAATACCCCGTAGGCT
GAGGAGCAAAAGGAGGAATCCGCCCGAGGAGGGGCTCGCGTCTGATTAGCTAGTTG
GTGAGGCAATAGCTTACCAAGGCGATGATCAGTAGCTGGTCCGAGAGGATGATCAG
CCACACTGGGACTGAGACACGGCCCAGACTCCTACGGGAGGCAGCAGTGGGGAATT
TTCCGCAATGGGCGAAAGCCTGACGGAGCAATGCCGCGTGGAGGTAGAAGGCCCAC
GGGTCGTGAACTTCTTTTCCCGGAGAAGAAGCAATGACGGTATCTGGGGAATAAGC
ATCGGCTAACTCTGTGCCAGCAGCCGCGGTAATACAGAGGATGCAAGCGTTATCCG
GAATGATTGGGCGTAAAGCGTCTGTAGGTGGCTTTTTAAGTCCGCCGTCAAATCCCA
GGGCTCAACCCTGGACAGGCGGTGGAAACTACCAAGCTGGAGTACGGTAGGGGCAG
AGGGAATTTCCGGTGGAGCGGTGAAATGCGTAGAGATCGGAAAGAACACCAACGGC
GAAAGCACTCTGCTGGGCCGACACTGACACTGAGAGACGAAAGCTAGGGGAGCGA
ATGGGATTAGATACCCCAGTAGTCCTAGCCGTAAACGATGGATACTAGGCGCTGTGC
GTATCGACCCGTGCAGTGCTGTAGCTAACGCGTTAAGTATCCCGCCTGGGGAGTACG

TTCGCAAGAATGAAACTCAAAGGAATTGACGGGGGCCCGCACAAGCGGTGGAGCAT
GTGGTTTAATTCGATGCAAAGCGAAGAACCTTACCAGGGATTGACATGCCGCGAAT
CCTCTTGAAAGAGAGGGGTGCCTTCGGGAACGCGGACACAGGTGGTGCATGGCTGT
CGTCAGCTCGTGCCGTAAGGTGTTGGGTTAAGTCCCGCAACGAGCGCAACCCTCGTG
TTTAGTTGCCACCGTTGAGTTTGGAACCCTGAGCAGACTGCCGGTGATAAGCCGGAG
GAAGGTGAGGATGACGTCAAGTCATCATGCCCCTTATGCCCTGGGCGACACACGTG
CTACAATGGCCGGGACAAAGGGTCGCGATCCCGCGAGGGTGAGCTAACTCCAAAAA
CCCGTCCTCAGTTCGGATTGCAGGCT
>P011_WF09_k90.1305 [Chlorotettix rugicollis, 16S]
TCTCATGGAGAGTTCGATCCTGGCTCAGGATGAACGCTGGCGGCATGCTTAACACAT
GCAAGTCGGACGGGAAGTGGTGTTTCCAGTGGCGGACGGGTGAGTAACGCGTAAGA
ACCTGCCCTTGGGAGGGGAACAACAGCTGGAAACGGCTGCTAATACCCCGTAGGCT
GAGGAGCAAAAGGAGGAATCCGCCCGAGGAGGGGCTCGCGTCTGATTAGCTAGTTG
GTGAGGCAATAGCTTACCAAGGCGATGATCAGTAGCTGGTCCGAGAGGATGATCAG
CCACACTGGGACTGAGACACGGCCCAGACTCCTACGGGAGGCAGCAGTGGGGAATT
TTCCGCAATGGGCGAAAGCCTGACGGAGCAATGCCGCGTGGAGGTAGGAGGCCTAC
GGGTCGTGAACTTCTTTTCCCGGAGAAGAAGCAATGACGGTATCTGGGGAATAAGC
ATCGGCTAACTCTGTGCCAGCAGCCGCGGTAATACAGAGGATGCAAGCGTTATCCG
GAATGATTGGGCGTAAAGCGTCTGTAGGTGGCTTTTTAAGTCCGCCGTCAAATCCCA
GGGCTCAACCCTGGACAGGCGGTGGAAACTGCCAAGCTCGAGTACGGTAGGGGCAG
AGGGAATTTCCGGTGGAGCGGTGAAATGCGTAGAGATCGGAAAGAACACCAACGGC
GAAAGCACTCTGCTGGGCCGACACTGACACTGAGAGACGAAAGCTAGGGGAGCGA
ATGGGATTAGATACCCCAGTAGTCCTAGCCGTAAACGATGGATACTGGGCGCTGTGC
GTATCGACCCGTGCAGTGCTGTAGCTAACGCGTTAAGTATCCCGCCTGGGGAGTACG
TTCGCAAGAATGAAACTCAAAGGAATTGACGGGGGCCCGCACAAGCGGTGGAGCAT
GTGGTTTAATTCGATGCAAAGCGAAGAACCTTACCAGGGCTTGACATGCCGCGAATC
CTCTTGAAAGAGAGGGGTGCCTTCGGGAACGCGGACACAGGTGGTGCATGGCTGTC
GTCAGCTCGTGCCGTAAGGTGTTGGGTTAAGTCCCGCAACGAGCGCAACCCTCGTGT
TTAGTTGCCACTGTTGAGTTTGGAACCCTGAGCAGACTGCCGGTGATAAGCCGGAGG
AAGGTGAGGATGACGTCAAGTCATCATGCCCCTTATGCCCTGGGCGACACACGTGCT
ACAATGGCCGGGACAAAGGGTCGCGATCCCGCGAGGGTGAGCTAACCCCAAAAACC
CGTCCTCAGTTCGGATTGCAGGCTGCAACTCGCCTGCATGAAGGCGGAATCGCTAGT
AATCGCCGGTCAGCCATACGGCGGTGAATTCGTTCCCGGGCCTTGTACACACCGCCC
GTCACACTATGGGAGCTGGCCATGCCCGAAGTCGTTACCTTAACCGCAAGGAGGGG
GATGCCGAAGGCAGGGCTAGTGACTGGAGTGAAGTCGTAACAAGGTAGCCGTACTG
GAAGGTGCGGCTGGATCACCTCCTTT
>P004_WF05_k90.2455 [Copididonus hyalinipennis, 16S (partial)]
GATCCTGGCTCAGGATGAACGCTGGCGGCATGCTTAACACATGCAAGTCGAACGGG
AAGTGGTGTTTCCAGTGGCGAACGGGTGAGTAACGCGTAAGAACCTGCCCTTGGGA
GGGGAACAACAACTGGAAACGGTTGCTAATACCCCGTAGGCTGAGGAGCAAAAGG
AGAAATCCGCCCAAGGAGGGGCTCGCGTCTGATTAGCTAGTTGGTGAGGCAATAGC
TTACCAAGGCGATGATCAGTAGCTGGTCCGAGAGGATGATCAGCCACACTGGGACT
GAGACACGGCCCAGACTCCTACGGGAGGCAGCAGTGGGGAATTTTCCGCAATGGGC
GAAAGCCTGACGGAGCAATGCCGCGTGGAGGTGGAAGGCCTACGGGTCGTCAACTT
CTTTTCTCGGAGAAGAAACAATGACGGTATCTGAGGAATAAGCATCGGCTAACTCTG
TGCCAGCAGCCGCGGTAAGACAGAGGATGCAAGCGTTATCCGGAATGATTGGGCGT

AAAGCGTCTGTAGGTGGCTTTTCAAGTCCGCCGTCAAATCCCAGGGCTCAACCCTGG
ACAGGCGGTGGAAACTACCAAGCTGGAGTACGGTAGGGGCAGAGGGAATTTCCGGT
GGAGCGGTGAAATGCATTGAGATCGGAAAGAACACCAACGGCGAAAGCACTCTGCT
GGGCCGACACTGACACTGAGAGACGAAAGCTAGGGGAGCAAATGGGATTAGAGAC
CCCAGTAGTCCTAGCCGTAAACGATGGATACTAGGTGCTGTGCGACTCGACCCGTGC
AGTGCTGTAGCTAACGCGTTAAGTATCCCGCCTGGGGAGTACGTTCGCAAGAATGA
AACTCAAAGGAATTGACGGGGGCCCGCACAAGCGGTGGAGCATGTGGTTTAATTCG
ATGCAAAGCGAAGAACCTTACCAGGGCTTGACATGCCGCGAATCCTCTTGAAAGAG
AGGGGTGCCCTCGGGAACGCGGACACAGGTGGTGCATGGCTGTCGTCAGCTCGTGC
CGTAAGGTGTTGGGTTAAGTCTCGCAACGAGCGCAACCCTCGTGTTTAGTTGCCACT
ATGAGTTTGGAACCCTGAACAGACCGCCGGTGTTAAGCCGGAGGAAGGAGAGGATG
AGGCCAAGTCATCATGCCCCTTATGCCCTGGGCGACACACGTGCTACAATGGGCGG
GACAAAGGGTCGCGATCTCGCGAGGGTGAGCTAACTCCAAAAACCCGTCCTCAGTT
CGGATTGCAGGCTGCAACTCGCCTGCATGAAGCAGGAATCGCTAGTAATCGCCGGT
CAGCCATACGGCGGTGAATCCGTTCCCGGGCCTTGTACACACCGCCCGTCACACTAT
AGGAGCTGGCCAGGTTTGAAGTCATTACCCTTAACCGTAAGGAGGGGGATGCCTAA
GGCTAGGCTTGCGACTGGAGTGAAGTCGTAACAAGGTAGCCGTACTGGAAGGTGCG
GCTGGATCACCTCCTTT
>P019_WE07_k90.1014 [Neodartus vinulus, 16S]
TCTCATGGAGAGTTCGATCCTGGCTCAGGATGAACGCTGGCGGCATGCTTAACACAT
GCAAGTCGGACGGGAAGTGGTGTTTCCAGTGGCGGACGGGTGAGTAACGCGTAAGA
ACCTGCCCTTGGGAGGGGAACAACAACTGGAAACGGTTGCTAATACCCCGTAGGCT
GAGGAGCAAAAGGAGGAATCCGCCCGAGGAGGGGCTTGCGTCTGATTAGCTAGTTG
GTGAGGTAATAGCTTACCAAGGCGATGATCAGTAGCTGGTCCGAGAGGATGATCAG
CCACACTGGGACTGAGACACGGCCCAGACTCCTACGGGAGGCAGCAGTGGGGAATT
TTCCGCAATGGGCGAAAGCCTGACGGAGCAATGCCGCGTGGAGGTAGAAGGCCCAC
GGGTCGTGAACTTCTTTTCTCGGAGAAGAAACAATGACGGTATCTGAGGAATAAGC
ATCGGCTAACTCTGTGCCAGCAGCCGCGGTAAGACAGAGGATGCAAGCGTTATCCG
GAATGATTGGGCGTAAAGCGTCTGTAGGTGGCTTTTCAAGTCCGCCGTCAAATCCCA
GGGCTCAACCCTGGACAGGCGGTGGAAACTACCAAGCTGGAGTACGGTAGGGGCAG
AGGGAATTTCCGGTGGAGCGGTGAAATGCGTAGAGATCGGAAAGAACACCAACGGC
GAAAGCACTCTGCTGGGCCGACACTGACACTGAGAGACGAAAGCTAGGGGAGCAA
ATGGGATTAGATACCCCAGTAGTCCTAGCCGTAAACGATGGATACTAGGCGCTGTGC
GTATCGACCCGTGCAGTGCTGTAGCTAACGCGTTAAGTATCCCGCCTGGGGAGTACG
TTCGCAAGAATGAAACTCAAAGGAATTGACGGGGGCCCGCACAAGCGGTGGAGCAT
GTGGTTTAATTCGATGCAAAGCGAAGAACCTTACCAGGGCTTGACATGCCGTGAATC
CTCTTGAAAGAGAGGGGTGCCTTCGGGAACGCGGACACAGGTGGTGCATGGCTGTC
GTCAGCTCGTGCCGTAAGGTGTTGGGTTAAGTCCCGCAACGAGCGCAACCCTCGTGT
TTAGTTGCCACCATTGAGTTTGGAACCCTGAACAGACTGCCGGTGATAAGCCGGAGG
AAGGTGGGGATGACGTCAAGTCATCATGCCCCTTATGCCCTGGGCGACACACGTGCT
ACAATGGCCGGGACAAAGGGTCGCGATCCCGCGAGGGTGAGCTAACCCCAAAAACC
CGTCCTCAGTTCGGATTGCAGGCTGCAACTCGCCTGCATGAAGCCGGAATCGCTAGT
AATCGCCGGTCAGCCATACGGCGGTGAATTCGTTCCCGGGCCTTGTACACACCGCCC
GTCACACTATGGGAGCTGGCCATGCCCGAAGTCGTTACCTTAACCGCAAGGAGGGG
GATGCCGAAGGCGGGGCTAGTGACTGGAGTGAAGTCGTAACAAGGTAGCCGTACTG
GAAGGTGCGGCTGGATCACCTCCTTT

>P006_WE09_k90.1355 [Scaphoideini New Genus ZA3 n. sp. 1, 16S (partial)]
AGGGGCAGAGGGAATTTCCGGTGGAGCGGTGAAATGCGTAGAGATCGGAAAGAAC
ACCAACGGCGAAAGCACTCTGCTGGGCCGACACTGACACTGAGAGACGAAAGCTAG
GGGAGCGAATGGGATTAGATACCCCAGTAGTCCTAGCCGTAAACGATGGATACTAG
GCGCTGTGCGTATCGACCCGTGCAGTGCTGTAGCTAACGCGTTAAGTATCCCGCCTG
GGGAGTACGTTCGCAAGAATGAAACTCAAAGGAATTGACGGGGGCCCGCACAAGCG
GTGGAGCATGTGGTTTAATTCGATGCAAAGCGAAGAACCTTACCAGGGATTGACAT
GCCGCGAATCCTCTTGAAAGAGAGGGGTGCCTTCGGGAACGCGGACACAGGTGGTG
CATGGCTGTCGTCAGCTCGTGCCGTAAGGTGTTGGGTTAAGTCCCGCAACGAGCGCA
ACCCTCGTGTTTAGTTGCCACCGTTGAGTTTGGAACCCTGAGCAGACTGCCGGTGAT
AAGCCGGAGGAAGGTGAGGATGACGTCAAGTCATCATGCCCCTTATGCCCTGGGCG
ACACACGTGCTACAATGGCCGGGACAAAGGGTCGCGATCCCGCGAGGGTGAGCTAA
CTCCAAAAACCCGTCCTCAGTTCGGATTGCAGGCTGCAACTCGCCTGCATGAAGCCG
GAATCGCTAGTAATCGCCGGTCAGCCATACGGCGGTGAATTCGTTCCCGGGCCTTGT
ACACACCGCCCGTCACACTATGGGAGCTGGCCATGCCCGAAGTCGTTACCTTAACCG
CAAGGAGGGGGATGCCGAAGGCAGGGCTAGTGACTGGAGTGAAGTCGTAACAAGG
TAGCCGTACTGGAAGGTGCGGCTGGATCACCTCCTTT
>P015_WH05_k90.23 [Scaphoideini New Genus ZA5 n. sp. 1, 16S (partial)]
TGGGAGGGGAACAACAGCTGGAAACGGCTGCTAATACCCCGTAGGCTGAGGAGCAA
AAGGAGGAATCCGCCCGAGGAGGGGCTCGCGTCTGATTAGCTAGTTGGTGAGGCAA
TAGCTTACCAAGGCGATGATCAGTAGCTGGTCCGAGAGGATGATCAGCCACACTGG
GACTGAGACACGGCCCAGACTCCTACGGGAGGCAGCAGTGGGGAATTTTCCGCAAT
GGGCGAAAGCCTGACGGAGCAATGCCGCGTGGAGGTAGAAGGCCCACGGGTCGTG
AACTTCTTTTCCCGGAGAAGAAGCAATGACGGTATCTGGGGAATAAGCATCGGCTA
ACTCTGTGCCAGCAGCCGCGGTAATACAGAGGATGCAAGCGTTATCCGGAATGATT
GGGCGTAAAGCGTCTGTAGGTGGCTTTTTAAGTCCGCCGTCAAATCCCAGGGCTCAA
CCCTGGACAGGCGGTGGAAACTACCAAGCTGGAGTACGGTAGGGGCAGAGGGAATT
TCCGGTGGAGCGGTGAAATGCGTAGAGATCGGAAAGAACACCAACGGCGAAAGCA
CTCTGCTGGGCCGACACTGACACTGAGAGACGAAAGCTAGGGGAGCGAATGGGATT
AGATACCCCAGTAGTCCTAGCCGTAAACGATGGATACTAGGCGCTGTGCGTATCGAC
CCGTGCAGTGCTGTAGCTAACGCGTTAAGTATCCCGCCTGGGGAGTACGTTCGCAAG
AATGAAACTCAAAGGAATTGACGGGGGCCCGCACAAGCGGTGGAGCATGTGGTTTA
ATTCGATGCAAAGCGAAGAACCTTACCAGGGATTGACATGCCGCGAATCCTCTTGA
AAGAGAGGGGTGCCTTCGGGAACGCGGACACAGGTGGTGCATGGCTGTCGTCAGCT
CGTGCCGTAAGGTGTTGGGTTAAGTCCCGCAACGAGCGCAACCCTCGTGTTTAGTTG
CCACCGTTGAGTTTGGAACCCTGAGCAGACTGCCGGTGATAAGCCGGAGGAAGGTG
AGGATGACGTCAAGTCATCATGCCCCTTATGCCCTGGGCGACACACGTGCTACAATG
GCCGGGACAAAGGGTCGCGATCCCGCGAGGGTGAGCTAACTCCAAAAACCCGTCCT
CAGTTCGGATTGCAGGCTGCAACTCGCCTGCATGAAGCCGGAATCGCTAGTAATCGC
CGGTCAGCCATACGGCGGTGAATTCGTTCCCGGGCCTTGTACACACCGCCCGTCACA
CTATGGGAGCTGGCCATGCCCGAAGTCG
>P003_WA09_k90.19172 [Osbornellus auronitens, 16S (partial)]
GTGGCGGACGGGTGAGTAACGCGTAAGAACCTGCCCTTGGGAGGGGAACAACAGCT
GGAAACGGCTGCTAATACCCCGTAGGCTGAGGAGCAAAAGGAGGAATCCGCCCGA
GGAGGGGCTCGCGTCTGATTAGCTAGTTGGTGAGGCAATAGCTTACCAAGGCGATG
ATCAGTAGCTGGTCCGAGAGGATGATCAGCCACACTGGGACTGAGACACGGCCCAG

ACTCCTACGGGAGGCAGCAGTGGGGAATTTTCCGCAATGGGCGAAAGCCTGACGGA
GCAATGCCGCGTGGAGGTAGAAGGCCCACGGGTCGTGAACTTCTTTTCCCGGAGAA
GAAGCAATGACGGTATCCGGGGAATAAGCATCGGCTAACTCTGTGCCAGCAGCCGC
GGTAATACAGAGGATGCAAGCGTTATCCGGAATGATTGGGCGTAAAGCGTCTGTAG
GTGGCTTTTTAAGTCCGCCGTCAAATCCCAGGGCTCAACCCTGGACAGGCGGTGGAA
ACTACCAAGCTGGAGTACGGTAGGGGCAGAGGGAATTTCCGGTGGAGCGGTGAAAT
GCGTAGAGATCGGAAAGAACACCAACGGCGAAAGCACTCTGCTGGGCCGACACTGA
CACTGAGAGACGAAAGCTAGGGGAGCGAATGGGATTAGATACCCCAGTAGTCCTAG
CCGTAAACGATGGATACTAGGCGCTGTGCGTATCGACCCGTGCAGTGCTGTAGCTAA
CGCGTTAAGTATCCCGCCTGGGGAGTACGTTCGCAAGAATGAAACTCAAAGGAATT
GACGGGGGCCCGCACAAGCGGTGGAGCATGTGGTTTAATTCGATGCAAAGCGAAGA
ACCTTACCAGGGCTTGACATGCCGCGAATCCTCTTGAAAGAGAGGGGTGCCTTCGGG
AACGCGGACACAGGTGGTGCATGGCTGTCGTCAGCTCGTGCCGTAAGGTGTTGGGTT
AAGTCCCGCAACGAGCGCAACCCTCGTGTTTAGTTGCCA
>P021_WA12_k29.7562 [Scaphoideus micellus, rbcL (partial)]
CATCGATGCCGTTCCTGGAGAAGACAATCAATATATTTGTTATGTAGCTTACCCCTT
AGACCTTTTTGAAGAAGGTTCTGTTACTAATATGTTTACTTCCATCGTGGGCAATGTA
TTTGGGTTCAAAGCCCTGCGTGCTCTACGTTTGGAGGATTTGCGAATCCCTGTTGCT
>P018_WE10_k29.11454 [Scaphytopius trilineatus, matK (partial)]
TTTCCCAATCTTCTAACTTCATTTCAAATATACTATTAAATGGAGGAAATCCAAAGCT
ATTTACAACTTGATAGATCTCAACAGCCCGGCTTTCTATATCCACTTATTTTTCAGGA
GTATATTTACGGGCTTGCTCATGATTATATTTTAAATCGATCTCGCTTGTTGGGAAAT
C
>P019_WB01_k29.56277 [Scaphytopius n. sp. GU1, rbcL (partial)]
AGCTGCCGAATCTTCTACTGGTACATGGACAACTGTATGGACCGACGGACTTACCAG
TCTTGATCGTTACAAAGGACGATGCTACCACATCGATGCCGTTCCTGGAGAAGACAA
TCAATATATTTGTTATGTAGCTTACCCCTTAGACCTTTTTGAAGAAGGTTCTGTTACT
AATATGTTTACTTCCATTGTGGGTAATGTATTTGGGTTCAAAGCCCTGCGTG
>P021_WA09_k29.27824 [Bumka amphitryon, rbcL (partial)]
TGTATTTGGGTTCAAAGCCCTGCGTGCTCTACGTTTGGAGGATTTGCGAATCCCTGTT
GCTTATATAAAAACTTTCCAAGGCCCGCCTCACGGTATCCAAGTTGAGAGAGATAAA
TTGAACAAGTATGGCCGTCCTCTACTGGGATGCACTATTAAGCCGAAATTGGGGTTA
TCTGCTAAAAACTATGGTCGAGCGGTTTATGAATGTCTTCGCGGTGGACTTGATTTT
AC
>P019_WB02_k29.6303 [Cyrta orientalis, rbcL (partial)]
CCCCAGGATACCGATATCTTGGCAGCATTCCGAGTAACTCCTCAACCAGGAGTTCCG
TCAGAAGAAGCAGGAGCCGCAGTAGCTGCCGAATCTTCTACTGGTACATGGACAAC
TGTATGGACCGACGGACTTACCAGTCTTGATCGTTACAAAGGACGATGCTACCCAC
>P021_WG10_k50.79047 [Vartini New Genus n. sp., matK (partial)]
GTATCGCGCTGTGTATCATTCGAAAATTCCCAAATTTTATACCTTCATTTCAAATATA
CTATTCAATGGAGGAAATCCAAGGCTATTTACAGCTTGATAGATCTCAACAGCCCGG
CTTTATATATCCACTTATCTTTCAGGAGTATATTTACGGACTTGCTCATGATTATAGT
TTAAATCGATCTCG
>P018_WC03_k90.1501 [Chileanoscopus sp., 16S]
TCTCATGGAGAGTTCGATCCTGGCTCAGGATGAACGCTGGCGGCATGCTTAACACAT
GCAAGTCGGACGGGAAGTGGTGTTTCCAGTGGCGGACGGGTGAGTAACGCGTAAGA

ACCTGCCCTTGGGAGGGGAACAACAGCTGGAAACGGCTGCTAATACCCCGTAGGCT
GAGGAGCAAAAGGAGGAATCCGCCCGAGGAGGGGCTCGCGTCTGATTAGCTAGTTG
GTGAGGTAATAGCTTACCAAGGCGATGATCAGTAGCTGGTCCGAGAGGATGATCAG
CCACACTGGGACTGAGACACGGCCCAGACTCCTACGGGAGGCAGCAGTGGGGAATT
TTCCGCAATGGGCGAAAGCCTGACGGAGCAATGCCGCGTGGAGGTAGAAGGCCCAC
GGGTCATGAACTTCTTTTCCCGGAGAAGAAGCAATGACGGTATCTGGGGAATAAGC
ATCGGCTAACTCTGTGCCAGCAGCCGCGGTAATACAGAGGATGCAAGCGTTATCCG
GAATGATTGGGCGTAAAGCGTCTGTAGGTGGCTTTTTAAGTCCGCCGTCAAATCCCA
GGGCTCAACTCTGGACAGGCGGTGGAAACTACCAAGCTGGAGTACGGTAGGGGCAG
AGGGAATTTCCGGTGGAGCGGTGAAATGCGTAGAGATCGGAAAGAACACCAACGGC
GAAAGCACTCTGCTGGGCCGACACTGACACTGAGAGACGAAAGCTAGGGGAGCGA
ATGGGATTAGATACCCCAGTAGTCCTAGCCGTAAACGATGGATACTAGGCGCTGTGC
GTATCGACCCGTGCAGTGCTGTAGCTAACGCGTTAAGTATCCCGCCTGGGGAGTACG
TTCGCAAGAATGAAACTCAAAGGAATTGACGGGGGCCCGCACAAGCGGTGGAGCAT
GTGGTTTAATTCGATGCAAAGCGAAGAACCTTACCAGGGCTTGACATGCCGCGAATC
CTCTTGAAAGAGAGGGGTGCCTTCGGGAACGCGGACACAGGTGGTGCATGGCTGTC
GTCAGCTCGTGCCGTAAGGTGTTGGGTTAAGTCCCGCAACGAGCGCAACCCTCGTGT
TTAGTTGCCATCATTGAGTTTGGAACCCTGAACAGACTGCCGGTGATAAGCCGGAGG
AAGGTGAGGATGACGTCAAGTCATCATGCCCCTTATGCCCTGGGCGACACACGTGCT
ACAATGGCCGGGACAAAGGGTCGCGATCCCGCGAGGGTGAGCTAACTCCAAAAACC
CGTCCTCAGTTCGGATTGCAGGCTGCAACTCGCCTGCATGAAGCCGGAATCGCTAGT
AATCGCCGGTCAGCCATACGGCGGTGAATCCGTTCCCGGGCCTTGTACACACCGCCC
GTCACACTATGGGAGCTGGCCATGCCCGAAGTCGTTACCTTAACCGCAAGGAGGGG
GATGCCGAAGGCAGGGCTAGTGACTGGAGTGAAGTCGTAACAAGGTAGCCGTACTG
GAAGGTGCGGCTGGATCACCTCCTTT
>P021_WB01_k29.8312 [Ipoella porriginosa, matK (partial)]
ATACTTTGGTTTGACTATATCGCACTATGTATCATTTGATAATCCCAAAATCTTCTAC
CTTTGGTTCAAATCTAATATCAAATGGAGGAAATCAAAAGATATTTACAGCTTGATA
GATCTCAACAACACGGCTTTCTATATCCACTTATCTTTCAGGAGTATATTTA
>P021_WB01_k50.7154 [Ipoella porriginosa, matK (partial)]
AAATTCCCAAATCTTATACCTTCATTTCAAATATACTATTAAATGGAGGAAATCCAA
GGCTATTTACAGCTTGATAGATCTCAACAGCCCGGCTTTCTATATCCACTTATCTTTC
AGGAGTATATTTACGGACTTGCTCATGATTATAGTTTAAATCGATCTCGCTTGTTGAA
AAATCCGGGTTATGACAATAAATACAG
>P019_WG01_k29.43585 [Katipo rubrivenosa, matK (partial)]
AATATACTATTAAATGGAGGAAATCCAAAGCTATTTACAACTTGATAGATCTCAACA
GCCCGGCTTTCTATATCCACTTATTTTTCAGGAGTATATTTACGGGCTTGCTCATGAT
TATATTTTAAATCGATCTCGCTTGTTGGGAAATCCGGGTT
>P019_WG01_k90.3824 [Katipo rubrivenosa, matK (partial)]
TGGTTTAACTATATCGCACTATGTATCATTTGATAATCCCAAAATCTTCTACCTTTGG
TTCAAATCTAATATTAAATGGAGGAAATCCAAAGATATTTACAGCTTGATAGATCTC
AACAACACGGCTTTCTATATCCACTTATCTTTCAGGAGTATATTTATGCACTTGCTCA
TGATCATAGTTTAAACCGATCTATTTTGTTGGAAAATCTAGGTTATGACAATCAATTC
AGTTTCCTAATTGTGAAACGTTTAATTACTC
>P019_WE12_k29.19536 [Idiocerus sp., rbcL (partial)]

TTACCAGTCTTGATCGTTACAAAGGACGATGCTACCACATCGATGCCGTTCCTGGAG
AAGACAATCAATATATTTGTTATGTAGCTTACCCCTTAGACCTTTTTGAAGAAGGTTC
TGTTACTAATATGTTTACTTCCATCGTGGGCAATGTATTTGGGTTCAAAGCCCTGCGT
G
>P019_WD03_k90.1378 [Idiocerini New Genus MG1 n. sp. 2, matK (partial)]
GTTGATAAGTTTACCCCCGTCTCCGAGGTATTCTATTCTTACTAGAATACTTTGGTTT
AACTATATCGCACTATGTATCATTTGATAATCCCAAAATCTTCTACCTTTGGTTCAAA
TCTAATATCAAATGGAGGAAATCCAAAGATATTTACAGCTTGATAGATCTCAACAAC
ACGGCTTTCTATATCCACTTATCTTTCAGGAGTATATTTATGCACTTGCTCATGATCA
TAGTTTAAACCGATCTATTTTGTTGGAAAATCCAGGTTATGACAATCAATTCAGTTTC
CTAATTGTGAAACGTTTAATTACTCGAATGTATCAACAAAATCATTTTATTATTTTTG
CTAATGATTCTAATCAAAA
>P019_WC07_k29.41635 [Rhytidodus sp., rbcL (partial)]
CAGTCTTGATCGTTACAAAGGACGATGCTACCACATCGATGCCGTTCCTGGAGAAGA
CAATCAATATATTTGTTATGTAGCTTACCCCTTAGACCTTTTTGAAGAAGGTTCTGTT
ACTAATATGTTTACTTCCATTGTGGGTAATGTATTTGGGTTCA
>P019_WC07_k29.12585 [Rhytidodus sp., rbcL (partial)]
CGCGGTGGACTTGATTTTACCAAAGATGACGAAAACGTGAACTCCCAACCATTTATG
CGTTGGAGAGACCGTTTCTTATTTTGTGCCGAAGCAATTTATAAAGCACAGGCCGAA
ACAGGTGAAATCAAAGGGCATTACTTGAATGCTACCGCAGGTACATGCGAAGAAAT
GATAAAAAGGGCTGTATTTGCCAGAGAATTGGGTGTTC
>P019_WD04_k50.14044 [Kopamerra haupti, matK (partial)]
ATGTATCATTTGATAATCCCAAAATCTTCTACCTTTGGTTCAAATCTAATATCAAATG
GAGGAAATCCAAAGATATTTACAGCTTGATAGATCTCAACAACACGGCTTTCTATAT
CCACTTATCTTTCAGGAGTATATTTATGCACTTGCTCATGATCATAGTTTAAACCGAT
AGATCGGAAGAGCACACGTCTGAACTCCAGTCACCTAAGGTCAT
>P019_WD07_k29.96960 [Macropsidius niger, rbcL (partial)]
AATGTATTTGGGTTCAAAGCCCTGCGTGCTCTACGTTTGGAGGATTTGCGAATCCCT
GTTGCTTATATAAAAACTTTCCAAGGCCCGCCTCACGGTATCCAAGTTGAGAGAGAT
AAATTGAACAAGTATGGCCGTCCTCTACTGGGATGCACTATTAAGCCGAAATTGGGG
TTATCTGCTAAAAACTATGGTCGAGCGGTTTATGAATGTCTTCGCGGTGGACTTGAT
TTTACCAAAGATGACGAAAACGTGAACTCCCAACCATTTATGCGTTGGAGAGACCGT
TTCTTATTTTGTGCCGAAGCAATTTATAAAGCACAGGCCGAAACAGGTGAAATCAAA
GG
>P016_WB05_k90.3300 [Processus bifasciatus, 16S (partial)]
GGTGGAAACTACCAAGCTGGAGTACGGTAGGGGCAGAGGGAATTTCCGGTGGAGCG
GTGAAATGCGTAGAGATCGGAAAGAACACCAACGGCGAAAGCACTCTGCTGGGCCG
ACACTGACACTGAGAGACGAAAGCTAGGGGAGCGAATGGGATTAGATACCCCAGTA
GTCCTAGCCGTAAACGATGGATACTAGGCGCTGTGCGTATCGACCCGTGCAGTGCTG
TAGCTAACGCGTTAAGTATCCCGCCTGGGGAGTACGTTCGCAAGAATGAAACTCAA
AGGAATTGACGGGGGCCCGCACAAGCGGTGGAGCATGTGGTTTAATTCGATGCAAA
GCGAAGAACCTTACCAGGGCTTGACATGCCGCGAATCTTCTTGAAAGAGAGGGGTG
CCTTCGGGAACGCGGACACAGGTGGTGCATGGCTGTCGTCAGCTCGTGCCGTAAGGT
GTTGGGTTAAGTCCCGCAACGAGCGCAACCCTCGTGTTTAGTTGCCGCCGTTGAGTT
TGGAACCCTGAACAGACTGCCGGTGATAAGCCGGAGGAAGGTGAGGATGACGTCAA
GTCATCATGCCCCTTATGCCCTGGGCGACACACGTGCTACAATGGCCGGGACAAAG

GGTCGCGATCCCGCGAGGGTAAGCTAACTCCAAAAACCCGTCCTCAGTTCGGATTGC
AGGCTGCAACTCGCCTGCATGAAGCCGGAATCGCTAGTAATCGCCGGTCAGCCATA
CGGCGGTGAATTCGTTCCCGGGCCTTGTACACACCGCCCGTCACACTATGGGAGCTG
GCCATGCCCGAAGTCGTTACCTTAACCACAAGGAGGGGGATGCCGAAGGCGGGGCT
AGTGACTGGAGTGAAGTCGTAACAAGGTAGCCGTACTGGAAGGTGTGGCTGGATCA
CCTCCTTT
>P019_WF05_k29.28927 [Antillonirvana freytagi, rbcL (partial)]
AATGTATTTGGGTTCAAAGCCCTGCGTGCTCTACGTTTGGAGGATTTGCGAATCCCT
GTTGCTTATATAAAAACTTTCCAAGGCCCGCCTCACGGTATCCAAGTTGAGAGAGAT
AAATTGAACAAGTATGGCCGTCCTCTACTGGGATGCACTATTAAGCCGAAATTGGGG
>P019_WF06_k29.4407 [Carchariacephalus sp., rbcL (partial)]
GTTCTGTTACTAATATGTTTACTTCCATTGTGGGTAATGTATTTGGGTTCAAAGCCCT
GCGTGCTCTACGTTTGGAGGATTTGCGAATCCCTGTTGCTTATATAAAAACTTTCCAA
GGCCCGCCTCACGGTATCCAAGTTGAGAGAGATAAATTGAACAAGTATGGCCGTCC
TCTACTGGGATGCACTATTAAGCCGAAATTGGGGTTATCTGCTAAAAACTATGGTCG
AGCGGTTTATGAATGTCTTCGCGGTGGACTTGATTTTACCAAAGATGACGAAAACGT
GAACTCCCAA
>P019_WF07_k29.85586 [Decursusnirvana sp., rbcL (partial)]
CCATTTATGCGTTGGAGAGACCGTTTCTTATTTGTGCCGAAGCAATTTATAAAGCA
CAGGCCGAAACAGGTGAAATCAAAGGGCATTACTTGAATGCTACCGCAGGTACATG
CGAAGAAATGATAAAAAGGGCTGTATTTGCCAGAGAATTGGGTGTTCCTA
>P016_WC04_k90.1150 [Kasunga n. sp. CA1, 16S]
TCTCATGGAGAGTTCGATCCTGGCTCAGGATGAACGCTGGCGGCATGCTTAACACAT
GCAAGTCGGACGGGAAGTGGTGTTTCCAGTGGCGGACGGGTGAGTAACGCGTAAGA
ACCTGCCCTTGGGAGGGGAACAACAGCTGGAAACGGCTGCTAATACCCCGTAGGCT
GAGGAGCAAAAGGAGGAATCCGCCCGAGGAGGGGCTCGCGTCTGATTAGCTAGTTG
GTGAGGCAATGGCTTACCAAGGCGATGATCAGTAGCTGGTCCGAGAGGATGATCAG
CCACACTGGGACTGAGACACGGCCCAGACTCCTACGGGAGGCAGCAGTGGGGAATT
TTCCGCAATGGGCGAAAGCCTGACGGAGCAATGCCGCGTGGAGGTAGAAGGCCCAC
GGGTCGTGAACTTCTTTTCCCGGAGAAGAAGCAATGACGGTATCTGGGGAATAAGC
ATCGGCTAACTCTGTGCCAGCAGCCGCGGTAATACAGAGGATGCAAGCGTTATCCG
GAATGATTGGGCGTAAAGCGTCTGTAGGTGGCTTTTTAAGTCCGCCGTCAAATCCCA
GGGCTCAACCCTGGACAGGCGGTGGAAACTACCAAGCTGGAGTACGGTAGGGGCAG
AGGGAATTTCCGGTGGAGCGGTGAAATGCGTAGAGATCGGAAAGAACACCAACGGC
GAAAGCACTCTGCTGGGCCGACACTGACACTGAGAGACGAAAGCTAGGGGAGCGA
ATGGGATTAGATACCCCAGTAGTCCTAGCCGTAAACGATGGATACTAGGCGCTGTGC
GTATCGACCCGTGCAGTGCTGTAGCTAACGCGTTAAGTATCCCGCCTGGGGAGTACG
TTCGCAAGAATGAAACTCAAAGGAATTGACGGGGGCCCGCACAAGCGGTGGAGCAT
GTGGTTTAATTCGATGCAAAGCGAAGAACCTTACCAGGGCTTGACATGCCGCGAATC
CTCTTGAAAGAGAGGGGTGCCTTCGGGAACGCGGACACAGGTGGTGCATGGCTGTC
GTCAGCTCGTGCCGTAAGGTGTTGGGTTAAGTCCCGCAACGAGCGCAACCCTCGTGT
TTAGTTGCCGCCGTTGAGTTTGGAACCCTGAACAGACTGCCGGTGATAAGCCGGAGG
AAGGTGAGGATGACGTCAAGTCATCATGCCCCTTATGCCCTGGGCGACACACGTGCT
ACAATGGCCGGGACAAAGGGTCGCGATCCCGCGAGGGTGAGCTAACTCCAAAAACC
CGTCCTCAGTTCGGATTGCAGGCTGCAACTCGCCTGCATGAAGCCGGAATCGCTAGT
AATCGCCGGTCAGCCATACGGCGGTGAATTCGTTCCCGGGCCTTGTACACACCGCCC

GTCACACTATGGGAGCTGGCCATGCCCGAAGTCGTTACCTTAACCGCAAGGAGGGG
GATGCCGAAGGCAGGGCTAGTGACTGGAGTGAAGTCGTAACAAGGTAGCCGTACTG
GAAGGTGCGGCTGGATCACCTCCTTT
>P019_WF09_k29.50343 [Kosasia typica, matK (partial)]
TTTCAAAATAGAATACCTTGTTTTGACTGTATCGCACTATGTATCTTTAAATAATCCA
AAAAACTCCCTGCTTTTTTTTAGTTTTCGGTCTAATTTGAAATGGAAGAATTCCAAAG
ATATATAGAACTAGATAGGTCTTGGCAACACAACTTTTTCTATCCACTTATCTTTCAG
GAATATATTTATGGATTTGCATATGATCATGGTTTAAATAAATCGATTTTGTTGGAA
AA
>P018_WA09_k90.17656 [Narecho sp., 16S]
TCTCATGGAGAGTTCGATCCTGGCTCAGGATGAACGCTGGCGGCATGCTTAACACAT
GCAAGTCGGACGGGAAGTGGTGTTTCCAGTGGCGGACGGGTGAGTAACGCGTAAGA
ACCTGCCCTTGGGAGGGGAACAACAGCTGGAAACGGCTGCTAATACCCCGTAGGCT
GAGGAGCAAAAGGAGGAATCCGCCCGAGGAGGGGCTCGCGTCTGATTAGCTAGTTG
GTGAGGTAATAGCTTACCAAGGCGATGATCAGTAGCTGGTCCGAGAGGATGATCAG
CCACACTGGGACTGAGACACGGCCCAGACTCCTACGGGAGGCAGCAGTGGGGAATT
TTCCGCAATGGGCGAAAGCCTGACGGAGCAATGCCGCGTGGAGGTAGAAGGCCCAC
GGGTCATGAACTTCTTTTCCCGGAGAAGAAGCAATGACGGTATCTGGGGAATAAGC
ATCGGCTAACTCTGTGCCAGCAGCCGCGGTAATACAGAGGATGCAAGCGTTATCCG
GAATGATTGGGCGTAAAGCGTCTGTAGGTGGCTTTTTAAGTCCGCCGTCAAATCCCA
GGGCTCAACTCTGGACAGGCGGTGGAAACTACCAAGCTCGAGTACGGTAGGGGCAG
AGGGAATTTCCGGTGGAGCGGTGAAATGCGTAGAGATCGGAAAGAACACCAACGGC
GAAAGCACTCTGCTGGGCCGACACTGACACTGAGAGACGAAAGCTAGGGGAGCGA
ATGGGATTAGATACCCCAGTAGTCCTAGCCGTAAACGATGGATACTAGGCGCTGTGC
GTATCGACCCGTGCAGTGCTGTAGCTAACGCGTTAAGTATCCCGCCTGGGGAGTACG
TTCGCAAGAATGAAACTCAAAGGAATTGACGGGGGCCCGCACAAGCGGTGGAGCAT
GTGGTTTAATTCGATGCAAAGCGAAGAACCTTACCAGGGCTTGACATGCCGCGAATC
CTCTTGAAAGAGAGGGGTGCCTTCGGGAACGCGGACACAGGTGGTGCATGGCTGTC
GTCAGCTCGTGCCGTAAGGTGTTGGGTTAAGTCCCGCAACGAGCGCAACCCTCGTGT
TTAGTTGCCATCATTGAGTTTGGAACCCTGAACAGACTGCCGGTGATAAGCCGGAGG
AAGGTGAGGATGACGTCAAGTCATCATGCCCCTTATGCCCTGGGCGACACACGTGCT
ACAATGGCCGGGACAAAGGGTCGCGATCCCGCGAGGGTGAGCTAACTCCAAAAACC
CGTCCTCAGTTCGGATTGCAGGCTGCAACTCGCCTGCATGAAGCCGGAATCGCTAGT
AATCGCCGGTCAGCCATACGGCGGTGAATCCGTTCCCGGGCCTTGTACACACCGCCC
GTCACACTATGGGAGCTGGCCATGCCCGAAGTCGTTACCTTAACCGCAAGGAGGGG
GATGCCGAAGGCAGGGCTAGTGACTGGAGTGAAGTCGTAACAAGGTAGCCGTACTG
GAAGGTGCGGCTGGATCACCTCCTTT
>P018_WA11_k50.3899 [Neonirvana sp., matK (partial)]
GTGTATCATTCGAAAATTCCCAAATCTTATGCCTTCATTTCAAATATACTATTAAATG
GAGGAAATCCAAGGCTATTTACAGCTTGATAGATCTCAACAGCCCGGCTTTCTATAT
CCACTTATCTTTCAGGAGTATATTTACGGACTTGCTCATGATTATAGTTTAAATCGAT
CTCGCTTGTTGAAAAATCCGGGTTATGACAATAAATACAGTTTACTACTTGTGAAAC
GTTTAATTACTCGAATGTATGG
>P019_WH06_k29.5190 [Friscanus friscanus, matK (partial)]
ATATACTAGTAAATGGAGGAAATCCAAAGCTATTTACAACTTGATCGATCTCAACAG
CCCGGCTTTCTCTATCCACTTATCTTTCAGGAATATATTTACGGGCTTGCTCATGATT

ATAGTTTAAATCGATCTTGCTTGTTGCAAAATACGGGTTATGACAATAAATATAAAT
ACAGTTTACTACTTGTGAAACG
>P018_WB01_k90.723 [Pagaronia tredecimpunctata, 16S (partial)]
GGAGTACGGTAGGGGCAGAGGGAATTTCCGGTGGAGCGGTGAAATGCGTAGAGATC
GGAAAGAACACCAACGGCGAAAGCACTCTGCTGGGCCGACACTGACACTGAGAGA
CGAAAGCTAGGGGAGCGAATGGGATTAGATACCCCAGTAGTCCTAGCCGTAAACGA
TGGATACTAGGCGCTGTGCGTATCGACCCGTGCAGTGCTGTAGCTAACGCGTTAAGT
ATCCCGCCTGGGGAGTACGTTCGCAAGAATGAAACTCAAAGGAATTGACGGGGGCC
CGCACAAGCGGTGGAGCATGTGGTTTAATTCGATGCAAAGCGAAGAACCTTACCAG
GGCTTGACATGCCGCGAATCCTCTTGAAAGAGAGGGGTGCCTTCGGGAACGCGGAC
ACAGGTGGTGCATGGCTGTCGTCAGCTCGTGCCGTAAGGTGTTGGGTTAAGTCCCGC
AACGAGCGCAACCCTCGTGTTTAGTTGCCACCGTTGAGTTTGGAACCCTGAGCAGAC
TGCCGGTGATAAGCCGGAGGAAGGTGAGGATGACGTCAAGTCATCATGCCCCTTAT
GCCCTGGGCGACACACGTGCTACAATGGCCGGGACAAAGGGTCGCGATCCCGCGAG
GGTGAGCTAACCCCAAAAACCCGTCCTCAGTTCGGATTGTAGGCTGCAACTCGCCTG
CATGAAGCCGGAATCGCTAGTAATCGCCGGTCAGCCATACGGCGGTGAATTCGTTCC
CGGGCCTTGTACACACCGCCCGTCACACTATGGGAGCTGGCCATGCCCGAAGTCGTT
ACCTTAACCACAAGGAGGGGGATGCCGAAGGCAGGGCTAGTGACTGGAGTGAAGTC
GTAACAAGGTAGCCGTACTGGAAGGTGCGGCTGGATCACCTCCTTT
>P019_WE05_k29.7629 [Pentoffia tridenta, rbcL (partial)]
TTCTACTGGTACATGGACAACTGTATGGACCGACGGACTTACCAGTCTTGATCGTTA
CAAAGGACGATGCTACCACATCGATGCCGTTCCTGGAGAAGACAATCAATATATTTG
TTATGTAGCTTACCCCTTAGACCTTTTTGAAGAAGGTTCTGTTACTAATATGTTTACT
TCCATTGTGGGTAATGTATTTGGGTTCAAAGCCCTGCGTGCTCTACGTTTGGAGGATT
TGCGAATCCCTGTTGCTTATATAAAAACTTTCCAAGGCCCGCCTCACGGTATCCAAG
TTGAGAGAGATAAATTGAACAAGTATGGCCGTCCTCTACTGGGATGCACTATTAAGC
CGAAATTGGGGTTATCTGCTAAAAACTATGGTCGAGCGGTTTATGAATGTCTTCGCG
GTGGACTTGATTTTACCAAAGATGACGAAAACGTGAACTCCCAACCATTTATGCGTT
GGAGAGACCGTTTCTTATTTTGTGCCGAAGCAATTTATAAAGCACAGGCCGAAACAG
GTGAAATCAAAGGGCATTACTTGAATGCTACCGCAGGTACATGCGAAGAAATGATA
AAAAGGGCTGTATTTGCCAGAGAATTGGGTGTTCCT
>P017_WF03_k50.644 [Jassulus granulosus, rbcL (partial)]
ATTGCGCGATGATTTTATTGAAAAAGATCGTTCTCGCGGTATCTTTTTCACTCAGGAC
TGGGTATCCATGCCAGGTGTTATACCGGTGGCTTCAGGGGGTATTCATGTTTGGCAT
ATGCCAGCTCTGACCGAAATCTTTGGAGATGATTCCGTATTACAATTTGGTGGAGGA
ACTTTAGGACATCCTTGGGGAAATGCACCTGGTGCAGCAGCTAATCGTGTGGCTTTA
GAAGCCTGTGTACAAGCTCG
>P019_WD01_k29.7116 [Agallidwipa n. sp., matK (partial)]
ATCTTCTACCTTTGGTTCAAATCTAATATCAAATGGAGGAAATCAAAAGATATTTAC
AGCTTGATAGATCTCAACAACACGGCTTTCTATATCCACTTATCTTTCAGGAGTATAT
TTATGCACTTGCTCATGATCATAGTTTAAACCGATCTATTAGATCGGAAGAGCACAC
GTCTGAA
>P019_WD01_k29.18541 [Agallidwipa n. sp., rbcL (partial)]
TTTTACCAAAGATGACGAAAACGTGAACTCCCAACCATTTATGCGTTGGAGAGACCG
TTTCTTATTTTGTGCCGAAGCAATTTATAAAGCACAGGCCGAAACAGGTGAAATCAA
AGGGCATTACTTGAATGCTACCGCAGGTACATGCGAAGAAATGAT

>P017_WE07_k50.14533 [Paranagallia takiyae, matK (partial)]
ACTTTTCTTTCCATGGATCACAAAGTGAGCGTATTTGGTATTTGGATATTATCCGTAT
CAATGACCTGGTGAATCCTCTTACTCTTAATTAATCATTAGACAAAATTAATAAACT
AGAAAAGATCGATATCAAGATAAAACTTGCACATTTTCCATTCTGAAATGTTCCATA
GTAGGTGAATCACTTTAACTAATTAAAAATTCTAAAAATTAGTGGACTTCCTCTTCG
GAATAGAAATTGGCTA
>P019_WA01_k29.120535 [Brasa sp., rbcL (partial)]
TTCAAAGCCCTGCGTGCTCTACGTTTGGAGGATTTGCGAATCCCTGTTGCTTATATAA
AAACTTTCCAAGGCCCGCCTCACGGTATCCAAGTTGAGAGAGATAAATTGAACAAG
TATGGCCGTCCTCTACTGGGATGCACTATTAAGCCGAAATTGGGGTTATCTGCTAAA
AACTATGGTCGAGCGGTTTATGAATGTCTTCGCGGTGGACTTGATTTTACCAAAGAT
GACGAAAACGTGAACTCCCAACCATTTATGCGTTGGAGAGACCGTTTCTTATTTTGT
GCCGAAGCAATTTATAAAGCACAGGCCGAAACAGGTGAAATCAAAGG
>P017_WE10_k90.1481 [Megophthalmus scabripennis, 16S (partial)]
ATGGAGAGTTCGATCCTGGCTCAGGATGAACGCTGGCGGCATGCTTAACACATGCA
AGTCGGACGGGAAGTGGTGTTTCCAGTGGCGGACGGGTGAGTAACGCGTAAGAACC
TGCCCTTGGGAGGGGAACAACAGCTGGAAACGGCTGCTAATACCCCGTAGGCTGAG
GAGCAAAAGGAGGAATCCGCCCGAGGAGGGGCTCGCGTCTGATTAGCTAGTTGGTG
AGGTAATAGCTTACCAAGGCGATGATCAGTAGCTGGTCCGAGAGGATGATCAGCCA
CACTGGGACTGAGACACGGCCCAGACTCCTACGGGAGGCAGCAGTGGGGAATTTTC
CGCAATGGGCGAAAGCCTGACGGAGCAATGCCGCGTGGAGGTAGAAGGCCCACGG
GTCATGAACTTCTTTTCCCGGAGAAGAAGCAATGACGGTATCTGGGGAATAAGCATC
GGCTAACTCTGTGCCAGCAGCCGCGGTAATACAGAGGATGCAAGCGTTATCCGGAA
TGATTGGGCGTAAAGCGTCTGTAGGTGGCTTTTTAAGTCCGCCGTCAAATCCCAGGG
CTCAACTCTGGACAGGCGGTGGAAACTACCAAGCTGGAGTACGGTAGGGGCAGAGG
GAATTTCCGGTGGAGCGGTGAAATGCGTAGAGATCGGAAAGAACACCAACGGCGAA
AGCACTCTGCTGGGCCGACACTGACACTGAGAGACGAAAGCTAGGGTAGCGAATGG
GATTAGATACCCCAGTAGTCCTAGCCGTAAACGATGGATACTAGGCGCTGTGCGTAT
CGACCCGTGCAGTGCTGTAGCTAACGCGTTAAGTATCCCGCCTGGGGAGTACGTTCG
CAAGAATGAAACTCAAAGGAATTGACGGGGGCCCGCACAAGCGGTGGAGCATGTG
GTTTAATTCGATGCAAAGCGAAGAACCTTACCAGGGCTTGACATGCCGCGAATCCTC
TTGAAAGAGAGGGGTGCCTTCGGGAACGCGGACACAGGTGGTGCATGGCTGTCGTC
AGCTCGTGCCGTAAGGTGTTGGGTTAAGTCCCGCAACGAGCGCAACCCTCGTGTTTA
GTTGCCATCATTTAGTTTGGAACCCTGAACAGACTGCCGGTGATAAGCCGGAGGAA
GGTGAGGATGACGTCAAGTCATCATGCCCCTTATGCCCTGGGCGACACACGTGCTAC
AATGGCCGGGACAAAGGGTCGCGATCCCGCGAGGGTGAGCTAACTCCAAAAACCCG
TCCTCAGTTCGGATTGCAGGCTGCAACTCGCCTGCATGAAGCCGGAATCGCTAGTAA
TCGCCGGTCAGCCATACGGCGGTGAATCCGTTCCCGGGCCTTGTACACACCGCCCGT
CACACTATGGGAGCTGGCCATGCCCGAAGTCGTTACCTTAACCGCAAGGAGGGGGA
TGCCGAAGGCAGGGCTAGTGACTGGAGTGAAGTCGTAACAAGGTAGCCGTACTGGA
AGGTGCGGCTGGATCACCTCCTTT
>P017_WD11_k29.5965 [Makilingia panayensis, matK (partial)]
TGGAGGAAATCAAAAGCTATTTACAACTTGATAGATCTCAACAGCCCGGCTTTCTAT
ATCCACTTATTTTTCAGGAGTATATTTACGGGCTTGCTCATGATTATATTTTAAATAG
ATCTCGCTTGTTGGGAAATCCGGGTTATGACAATAAATACA
>P010_WA03_k90.383 [Alebrini New Genus PE1 n. sp., 16S]

TCTCATGGAGAGTTCGATCCTGGCTCAGGATGAACGCTGGCGGCATGCTTAACACAT
GCAAGTCGGACGGGAAGTGGTGTTTCCAGTGGCGGACGGGTGAGTAACGCGTAAGA
ACCTGCCCTTGGGAGGGGAACAACAGCTGGAAACGGCTGCTAATACCCCGTAGGCT
GAGGAGCAAAAGGAGGAATCCGCCCGAGGAGGGGCTCGCGTCTGATTAGCTAGTTG
GTGAGGCAATAGCTTACCAAGGCGATGATCAGTAGCTGGTCCGAGAGGATGATCAG
CCACACTGGGACTGAGACACGGCCCAGACTCCTACGGGAGGCAGCAGTGGGGAATT
TTCCGCAATGGGCGAAAGCCTGACGGAGCAATGCCGCGTGGAGGTAGAAGGCCCAC
GGGTCGTGAACTTCTTTTCCCGGAGAAGAAGCAATGACGGTATCTGGGGAATAAGC
ATCGGCTAACTCTGTGCCAGCAGCCGCGGTAATACAGAGGATGCAAGCGTTATCCG
GAATGATTGGGCGTAAAGCGTCTGTAGGTGGCTTTTTAAGTCCGCCGTCAAATCCCA
GGGCTCAACCCTGGACAGGCGGTGGAAACTACCAAGCTGGAGTACGGTAGGGGCAG
AGGGAATTTCCGGTGGAGCGGTGAAATGCGTAGAGATCGGAAAGAACACCAACGGC
GAAAGCACTCTGCTGGGCCGACACTGACACTGAGAGACGAAAGCTAGGGGAGCGA
ATGGGATTAGATACCCCAGTAGTCCTAGCCGTAAACGATGGATACTAGGCGCTGTGC
GTATCGACCCGTGCAGTGCTGTAGCTAACGCGTTAAGTATCCCGCCTGGGGAGTACG
TTCGCAAGAATGAAACTCAAAGGAATTGACGGGGGCCCGCACAAGCGGTGGAGCAT
GTGGTTTAATTCGATGCAAAGCGAAGAACCTTACCAGGGCTTGACATGCCGCGAATC
CTCTTGAAAGAGAGGGGTGCCTTCGGGAACGCGGACACAGGTGGTGCATGGCTGTC
GTCAGCTCGTGCCGTAAGGTGTTGGGTTAAGTCCCGCAACGAGCGCAACCCTCGTGT
TTAGTTGCCACCGTTGAGTTTGGAACCCTGAGCAGACTGCCGGTGATAAGCCGGAGG
AAGGTGAGGATGACGTCAAGTCATCATGCCCCTTATGCCCTGGGCGACACACGTGCT
ACAATGGACGGGACAAAGGGTCGCGATCCCGCGAGGGTGAGCTAACTCCAAAAACC
CGTCCTCAGTTCGGATTGCAGGCTGCAACTCGCCTGCATGAAGCCGGAATCGCTAGT
AATCGCCGGTCAGCCATACGGCGGTGAATTCGTTCCCGGGCCTTGTACACACCGCCC
GTCACACTATGGGAGCTGGCCATGCCCGAAGTCGTTACCTTAACCGCAAGGAGGGG
GATGCCGAAGGCAGGGCTAGTGACTGGAGTGAAGTCGTAACAAGGTAGCCGTACTG
GAAGGTGCGGCTGGATCACCTCCTTT
>P010_WB07_k29.36449 [Alconeura quadrimaculata, ITS1, 5.8S (partial)]
TCGAAACCTGCCCAGCAGAACGACCCGCGAACCTGTGACACCACGCCGAGGGGTGG
AGGAGGGGTGCGAGCCCCGATCTCCCTCCCTCTTTGGGTCGTGTGGGCTGTCGTTTC
TCCGTAAGTCCGTTGCCCGGGCCCTCGGCACGTGGCACGGCGCTTGGGGGGCGGCA
GCCTCGCAGACCCGACCAAACAACGAACCCGGCGCGAACGGCGCCAAGGAAATCA
AAACGGAACGGGCGTGCCTTCGCTTCACCGTCGGTGTTGCGGGGCGTCGTCAAAACT
CCGACCTCAAAACGACTCTCGGCAACGGATATCTCGGCTCTCGCATCGA
>P021_WC01_k90.811 [Dikrella n. sp. PE2, 16S (partial)]
GGCTCAACCCTGGACAGGCGGTGGAAACTACCAAGCTGGAGTACGGTAGGGGCAGA
GGGAATTTCCGGTGGAGCGGTGAAATGCGTAGAGATCGGAAAGAACACCAACGGCG
AAAGCACTCTGCTGGGCCGACACTGACACTGAGAGACGAAAGCTAGGGGAGCGAAT
GGGATTAGATACCCCAGTAGTCCTAGCCGTAAACGATGGATACTAGGCGCTGTGCGT
ATCGACCCGTGCAGTGCTGTAGCTAACGCGTTAAGTATCCCGCCTGGGGAGTACGTT
CGCAAGAATGAAACTCAAAGGAATTGACGGGGGCCCGCACAAGCGGTGGAGCATGT
GGTTTAATTCGATGCAAAGCGAAGAACCTTACCAGGGCTTGACATGCCGCGAATCCT
CTTGAAAGAGAGGGGTGCCTTCGGGAACGCGGACACAGGTGGTGCATGGCTGTCGT
CAGCTCGTGCCGTAAGGTGTTGGGTTAAGTCCCGCAACGAGCGCAACCCTCGTGTTT
AGTTGCCACCGTTGAGTTTGGAACCCTGAGCAGACTGCCGGTGATAAGCCGGAGGA
AGGTGAGGATGACGTCAAGTCATCATGCCCCTTATGCCCTGGGCGACACACGTGCTA

CAATGGCCGGGACAAAGGGTCGCGATCCCGCGAGGGTGAGCTAACTCCAAAAACCC
GTCCTCAGTTCGGATTGTAGGCTGCAACTCGCCTACATGAAGCCGGAATCGCTAGTA
ATCGCCGGTCAGCCATACGGCGGTGAATTCGTTCCCGGGCCTTGTACACACCGCCCG
TCACACTATGGGAGCTGGCCATGCCCGAAGTCGTTACCTTAACCGCAAGGAGGGGG
ATGCCGAAGGCAGGGCTAGTGACTGGAGTGAAGTCGTAACAAGGTAGCCGTACTGG
AAGGTG
>P015_WD12_k50.6889 [Dziwneono n. sp. 1, ITS1, 5.8S, ITS2]
TCGAATCCTGCCTAGCAGAATGACCAGAGAACCAGTAACAAACTCAATGGGGATGG
CGGGCTTTTCGCTCGACGTCCCTCGTCGCTCGAAATTTTGAACGGACGGCTACGGCG
GCCGTCAAGGTTGCGGGCGGCACAACGAACCCCGGCGCGGAACGCGCCAAGGAAC
CTGAACAAGAGAGCGGTGCTCCCATCACCCCAGACATGGTGCGTGCATGGGATGCC
ATGCAATCTCCTATTATTCACAACGACTCTCGGCAACGGATATCTCGGCTCTCGCAT
CGATGAAGAACGTAGCGAACTGCGATACTTGGTGTGAATTGCAGAATCCCGTGAAC
CATCGAGTCTTTGAACGCAAGTTGCGCCCGAAACCGTTTGGTCGAGGGCACGTTTGC
CTGGGTGTCACACATGGCGTTGCCCCTAATCCCTCGCCTCGAATCGGGGCGAGCGGG
ACTTGGGAGCGTAAGTTGGCCTCCCGCGACAACCTCGTCCCGGTTGGCCCAAAATCG
AGCGTCGGAGCGATTAGCACCACGACATTCGGTGGTTGATGAGACCCCAATGATCA
ATGTCGCGCGTGCCGCTCTCGCACACGCTCCACGAATCTACTCCTTACCAACG
>P019_WH02_k29.33792 [Forcipata loca, matK (partial)]
TTGGTTCAAATCTAATATTAAATGGAGGAAATCCAAAGATATTTACAGCTTGATAGA
TCTCAACAACACGGCTTTCTATATCCACTTATCTTTCAGGAGTATATTTATGCACTTG
CTCATGATCATAGTTTAAACCGATCTATTTGTTGGAAAATCTAGGTTATGACAATC
AATTCAGTTTCCTAATTGTGAAACGTTTAATTACTCGAATGTAT
>P015_WD06_k50.8991 [Kahaono negrea, 16S (partial)]
TGGGACTGAGACACGGCCCAGACTCCTACGGGAGGCAGCAGTGGGGAATTTTCCGC
AATGGGCGAAAGCCTGACGGAGCAATGCCGCGTGGAGGTAGAAGGCCCACGGGTC
GTGAACTTCTTTTCCCGGAGAAGAAGCAATGACGGTATCTGGGGAATAAGCATCGG
CTAACTCTGTGCCAGCAGCCGCGGTAATACAGAGGATGCAAGCGTTATCCGGAATG
ATTGGGCGTAAAGCGTCTGTAGGTGGCTTTTTAAGTCCGCCGTCAAATCCCAGGGCT
CAACCCTGGACAGGCGGTGGAAACTACCAAGCTGGAGTACGGTAGGGGCAGAGGG
AATTTCCGGTGGAGCGGTGAAATGCGTAGAGATCGGAAAGAACACCAACGGCGAAA
GCACTCTGCTGGGCCGACACTGACACTGAGAGGCGAAAGCTAGGGGAGCGAATGGG
ATTAGATACCCCAGTAGTCCTAGCCGTAAACGATGGATACTAGGCGCTGTGCGTATC
GACCCGTGCAGTGCTGTAGCTAACGCGTTAAGTATCCCGCCTGGGGAGTACGTTCGC
AAGAATGAAACTCAAAGGAATTGACGGGGGCCCGCACAAGCGGTGGAGCATGTGGT
TTAATTCGATGCAAAGCGAAGAACCTTACCAGGGCTTGACATGCCGCGAATCCTCTT
GAAAGAGAGGTGTGCCTTCGGGAACGCGGACACAGGTGGTGCATGGCTGTCGTCAG
CTCGTGCCGTAAGGTGTTGGGTTAAGTCCCGCAACGAGCGCAACCCTCGTGTTTAGT
TGCCACCGTTGAGTTTGGAACCCTGAACAGACTGCCGGTGATAAGCCGGAGGAAGG
TGAGGATGACGTCAAGTCATCATGCCCCTTATGCCCTGGGCGACACACGTGCTACAA
TGGCCGGGACAAAGGGTCGCGATCCCGCGAGGGTGAGCTAACTCCAAAAACCCGTC
CTCAGTTCGGATTGCAGGCTGCAACTCGCCTGCATGAAGCCGGAATCGCTAGTAATC
GCCGGTCAGCCATACGGCGGTGAATTCGTTCCCGGGCCTTGTACACACCGCCCGTCA
CACTATGGGAGCTGGCCATGCCCGAAGTCGTTACCTTAACCGCAAGGAGGGGGATG
CCGAAGGCAGGGCTAGTGACTGGAGTGAAGTCGTAACAAGGTAGCCGTACTGGAAG
GTGCGGCTGGATCACCTCCTTT

>P010_WA12_k90.2851 [Dikraneurini New Genus PE2 n. sp. 1, 16S]
TCTCATGGAGAGTTCGATCCCGGCTCAGGATGAACGCTGGCGGCATGCCTAACACAT
GCAAGTCGGACGGGAAGTGGTGTTTCCAGTGGCGGACGGGTGAGTAACGCGTAAGA
ACCTGCCCTTGGGAGGGGAACAACAACTGGAAACGGCTGCTAATACCCCGTAGGCT
GAGGAGCAAAAGGAGGAATCTGCCCGAGGAGGGGCTTGCGTCTGATTAGCTAGTTG
GTGAGGCAATAGCTTACCAAGGCGATGATCAGTAGCTGGTCCGAGAGGATGATCAG
CCACACTGGGACTGAGACACGGCCCAGACTCCTACGGGAGGCAGCAGTGGGGAATT
TTCCGCAATGGGCGAAAGCCTGACGGAGCAATGCCGCGTGGAGGTAGAAGGCCCAC
GGGTCGTGAACTTCTTTTCCCGGAGAAGAAGCAATGACGGTATCTGAGGAATAAGC
ATCGGCTAACTCTGTGCCAGCAGCCGCGGTAAGACAGAGGATGCAAGCGTTATCTG
GAATGATTGGGCGTAAGGCGTCTGTAGGTGGCTTTTCAAGTCCGCCGTCCAATCCCA
GGGCTCAACCCTGGACAGGCGGTGGAAACTACCAAGCTGGAGTACGGTAGGGGCAG
AGGGAATTTCCGGTGGAGCGGTGAAATGCGTAGAGATCGGAAAGAACACCAACGGC
GAAAGCACCCTGCTGGGCCGACACTGACACTGAGAGACGAAAGCTAGGGGAGCGA
ATGGGATTAGATACCCCAGTAGTCCTAGCCGTAAACGATGGATACTAGGCGCTGTGC
GTATCGACCCGTGCAGTGCTGTAGCTAACGCGTTAAGTATCCCGCCTGGGGAGTACG
TTCGCAAGAATGAAACTCAAAGGAATTGACGGGGGCCCGCACAAGCGGTGGAGCAT
GTGGTTTAATTCGATGCAAAGCGAAGAACCTTACCAGGGCTTGACATGCCGTGAATC
CTCTTGAAAGAGAGGGGTGCCTTCGGGAACGCGGACACAGGTGGTGCATGGCTGTC
GTCAGCTCGTGCCGTAAGGTGTTGGGTTAAGTCCCGCAACGAGCGCAACCCTCGTGT
TTAGTTGCCACCATTGAGTTTGGAACCCTGAACAGACCGCCGGTGATAAGCCGGAG
GAAGGTGAGGATGACGTCAAGTCATCATGCCCCCTATGCCCTGGGCGACACACGTG
TTACAATGGCCGGGACAAAGGGTCGCGATCCCGCCAGGGTGAGCTAACTCCAAAAA
CCCGTCCTAAGTTCGGATTGCAGGCTGCAACTCGCCTGCATGAAGCCGGAATCGCTA
GTAATCGCCGGTCAGCCATACGGCGGTGAATTCGTTCCCGGGCCTTGTACACACCGC
CCGTCACACTGTGGGAGCTGGCTATGCCCGAAGTCGTTACCTTAACCGCAAGGAGG
GGGATGCCGAAGGCGGGGCTAGTGACTGGAGTGAAGTCGTAACAAGGTAGCCGTAC
TGGAAGGTGCGGCTGGATCACCTCCTTT
>P021_WB12_k29.23922 [Dikraneurini New Genus PE4 n. sp. 2, rbcL (partial)]
ATAAATTGAACAAGTATGGCCGTCCTCTACTGGGATGCACTATTAAGCCGAAATTGG
GGTTATCTGCTAAAAACTATGGTCGAGCGGTTTATGAATGTCTTCGCGGTGGACTTG
ATTTTACCAAAGATGACGAAAACGTGAACTCCCAACCATTTATGCGTTGGAGAGACC
G
>P019_WB03_k50.3807 [Condensella filamenta, matK (partial)]
ACTGTATCGCACTGTGTATCATTGGAAAATTTCCCAATCTTCTACCTTCATTTCAAAT
ATACTATTAAATGGAGGAAATCCAAAGCTATTTACAACTTGATAGATCTCAACAGCC
CGGCTTTCTATATCCACTTATTTTTCAGGAGTATATTTACGGGCTTGCTCATGATTAT
ATTTTAAATCGATCTCGCTTGTTGGGAAATCCGGGTTATGACAATAAATACAGTTTA
CTATTTGTGAAACGTTTAATTACTCGAATGTATGGACAAAATCATTTTATTATTTTTG
CTAATGATTCTAATAAAAATTACTTTTTTGGTCGCAA
>P021_WC02_k29.21508 [Kotwaria n. sp. TW1, matK (partial)]
AGAATACTTTGGTTTAACTATATCGCACTATGTATCATTTGATAATCCCAAAATCTTC
TACCTTTGGTTCAAATCTAATATCAAATGGAGGAAATCCAAAGATATTTACAGCTTG
ATAGATCTCAACAACACGGCTTTCTATATCCACTTATCT
>P021_WB09_k90.1190 [Matsumurasca n. sp., 16S]

TCTCATGGAGAGTTCGATCCTGGCTCAGGATGAACGCTGGCGGCATGCTTAACACAT
GCAAGTCGGACGGGAAGTGGTGTTTCCAGTGGCGGACGGGTGAGTAACGCGTAAGA
ACCTGCCCTTGGGAGGGGAACAACAGCTGGAAACGGCTGCTAATACCCCGTAGGCT
GAGGAGCAAAAGGAGGAATCCGCCCGAGGAGGGGCTCGCGTCTGATTAGCTAGTTG
GTGAGGCAATAGCTTACCAAGGCGATGATCAGTAGCTGGTCCGAGAGGATGATCAG
CCACACTGGGACTGAGACACGGCCCAGACTCCTACGGGAGGCAGCAGTGGGGAATT
TTCCGCAATGGGCGAAAGCCTGACGGAGCAATGCCGCGTGGAGGTAGAAGGCCCAC
GGGTCGTGAACTTCTTTTCCCGGAGAAGAAGCAATGACGGTATCTGGGGAATAAGC
ATCGGCTAACTCTGTGCCAGCAGCCGCGGTAAGACAGAGGATGCAAGCGTTATCCG
GAATGATTGGGCGTAAAGCGTCTGTAGGTGGCTTTTAAGTCCGCCGTCAAATCCCA
GGGCTCAACCCTGGACAGGCGGTGGAAACTACCAAGCTGGAGTACGGTAGGGGCAG
AGGGAATTTCCGGTGGAGCGGTGAAATGCGTAGAGATCGGAAAGAACACCAACGGC
GAAAGCACTCTGCTGGGCCGACACTGACACTGAGAGACGAAAGCTAGGGGAGCGA
ATGGGATTAGATACCCCAGTAGTCCTAGCCGTAAACGATGGATACTAGGCGCTGTGC
GTATCGACCCGTGCAGTGCTGTAGCTAACGCGTTAAGTATCCCGCCTGGGGAGTACG
TTCGCAAGAATGAAACTCAAAGGAATTGACGGGGGCCCGCACAAGCGGTGGAGCAT
GTGGTTTAATTCGATGCAAAGCGAAGAACCTTACCAGGGCTTGACATGCCGCGAAC
CCTCTTGAAAGAGAGGGGTGCCTTCGGGAACGCGGACACAGGTGGTGCATGGCTGT
CGTCAGCTCGTGCCGTAAGGTGTTGGGTTAAGTCCCGCAACGAGCGCAACCCTCGTG
TTTAGTTGCCATCGTTGAGTTTGGAACCCTGAACAGACTGCCGGTGATAAGCCGGAG
GAAGGTGAGGATGACGTCAAGTCATCATGCCCCTTATGCCCTGGGCGACACACGTG
CTACAATGGCCGGGACAAAGGGTCGCGATCCCGCGAGGGTGAGCTAACCCCAAAAA
CCCGTCCTCAGTTCGGATTGCAGGCTGCAACTCGCCTGCATGAAGCCGGAATCGCTA
GTAATCGCCGGTCAGCCATACGGCGGTGAATCCGTTCCCGGGCCTTGTACACACCGC
CCGTCACACTATGGGAGCTGGCCATGCCCGAAGTCGTTACCTTAACCGCAAGGAGG
GGGATGCCGAAGGCAGGGCTAGTGACTGGAGTGAAGTCGTAACAAGGTAGCCGTAC
TGGAAGGTGCGGCTGGATCACCTCCTTT
>P010_WC05_k90.4532 [Empoascini New Genus PE1 n. sp. 1, 16S (partial)]
TCTCATGGAGAGTTCGATCCCGGCTCAGGATGAACGCTGGCGGCATGCCTAACACAT
GCAAGTCGGACGGGAAGTGGTGTTTCCAGTGGCGGACGGGTGAGTAACGCGTAAGA
ACCTGCCCTTGGGAGGGGAACAACAACTGGAAACGGCTGCTAATACCCCGTAGGCT
GAGGAGCAAAAGGAGGAATCTGCCCGAGGAGGGGCTTGCGTCTGATTAGCTAGTTG
GTGAGGCAATAGCTTACCAAGGCGATGATCAGTAGCTGGTCCGAGAGGATGATCAG
CCACACTGGGACTGAGACACGGCCCAGACTCCTACGGGAGGCAGCAGTGGGGAATT
TTCCGCAATGGGCGAAAGCCTGACGGAGCAATGCCGCGTGGAGGTAGAAGGCCCAC
GGGTCGTGAACTTCTTTTCCCGGAGAAGAAGCAATGACGGTATCTGAGGAATAAGC
ATCGGCTAACTCTGTGCCAGCAGCCGCGGTAAGACAGAGGATGCAAGCGTTATCTG
GAATGATTGGGCGTAAGGCGTCTGTAGGTGGCTTTTCAAGTCCGCCGTCCAATCCCA
GGGCTCAACCCTGGACAGGCGGTGGAAACTACCAAGCTGGAGTACGGTAGGGGCAG
AGGGAATTTCCGGTGGAGCGGTGAAATGCGTAGAGATCGGAAAGAACACCAACGGC
GAAAGCACCCTGCTGGGCCGACACTGACACTGAGAGACGAAAGCTAGGGGAGCGA
ATGGGATTAGATACCCCAGTAGTCCTAGCCGTAAACGATGGATACTAGGCGCTGTGC
GTATCGACCCGTGCAGTGCTGTAGCTAACGCGTTAAGTATCCCGCCTGGGGAGTACG
TTCGCAAGAATGAAACTCAAAGGAATTGACGGGGGCCCGCACAAGCGGTGGAGCAT
GTGGTTTAATTCGATGCAAAGCGAAGAACCTTACCA
>P021_WG04_k50.3697 [Empoascini New Genus T1 n. sp. 2, matK (partial)]

AAAGAGAGGATAGAGAATCTGTTGATAAGTTTACCCCCGTCTCCGAGGTATTCTATT
CTTACTAGAATACTTTGGTTTAACTATATCGCACTATGTATCATTTGATAATCCCAAA
ATCTTCTACCTTTGGTTCAAATCTAATATCAAATGGAGGAAATCCAAAGATATTTAC
AGCTTGATAGATCTCAACAACACGGCTTTCTATATCCACTTATCTTTCAGGAGTATAT
TTATGCACTTGCTCATGATCATAGTTTAAACCGATCTATTTTGTTGGAAAATCCAGGT
TATGACAATCAATTCAGTTTCCTAATTGTGAAACGTTTAATTACTCGAATGTATCAAC
AAAATCATT
>P015_WC10_k90.4551 [Unitra bufonis, 16S (partial)]
ACAGGCGGTGGAAACTGCCAAGCTGGAGTACGGTAGGGGCAGAGGGAATTTCCGGT
GGAGCGGTGAAATGCGTAGAGATCGGAAAGAACACCAACGGCGAAAGCACTCTGCT
GGGCCGACACTGACACTGAGAGACGAAAGCTAGGGGAGCGAATGGGATTAGATAC
CCCAGTAGTCCTAGCCGTAAACGATGGATACTGGGCGCTGTGCGTATCGACCCGTGC
AGTGCTGTAGCTAACGCGTTAAGTATCCCGCCTGGGGAGTACGTTCGCAAGAATGA
AACTCAAAGGAATTGACGGGGGCCCGCACAAGCGGTGGAGCATGTGGTTTAATTCG
ATGCAAAGCGAAGAACCTTACCAGGGCTTGACATGCCGCGAATCCTCTTGAAAGAG
AGGGGTGCCTTCGGGAACGCGGACACAGGTGGTGCATGGCTGTCGTCAGCTCGTGC
CGTAAGGTGTTGGGTTAAGTCCCGCAACGAGCGCAACCCTCGTGCTTAGTTGCCACC
GTTGAGTTTGGAACCCTGAGCAGACTGCCGGTGATAAGCCGGAGGAAGGTGAGGAT
GACGTCAAGTCATCATGCCCCTTATGCCCTGGGCGACACACGTGCTACAATGGCCGG
GACAAAGGGTCGCGATCCCGCGAGGGTGAGCTAACTCCAAAAACCCGTCCTCAGTT
CGGATTGCAGGCTGCAACTCGCCTGCATGAAGCCGGAATCGCTAGTAATCGCCGGT
CAGCCATACGGCGGTGAATTCGTTCCCGGGCCTTGTACACACCGCCCGTCACACTAT
GGGAGCTGACCATGCCCGAAGTCGTTACCTTAACCGCAAGGAGGGGGATGCCGAAG
GCAGGGCTAGTGACTGGAGTGAAGTCGTAACAAGGTAGCCGTACTGGAAGGTGCGG
CTGGATCACCTCCTTT
>P019_WC11_k90.2229 [Usharia sp., 16S (partial)]
GAACACCAACGGCGAAAGCACTCTGCTGGGCCGACACTGACACTGAGAGACGAAA
GCTAGGGGAGCGAATGGGATTAGATACCCCAGTAGTCCTAGCCGTAAACGATGGAT
ACTGGGCGCTGTGCGTATCGACCCGTGCAGTGCTGTAGCTAACGCGTTAAGTATCCC
GCCTGGGGAGTACGTTCGCAAGAATGAAACTCAAAGGAATTGACGGGGGCCCGCAC
AAGCGGTGGAGCATGTGGTTTAATTCGATGCAAAGCGAAGAACCTTACCAGGGCTT
GACATGCCGCGAATCCTCTTGAAAGAGAGGGGTGCCTTCGGGAACGCGGACACAGG
TGGTGCATGGCTGTCGTCAGCTCGTGCCGTAAGGTGTTGGGTTAAGTCCCGCAACGA
GCGCAACCCTCGTGTTTAGTTGCCACCGTTGAGTTTGGAACCCTGAGCAGACTGCCG
GTGATAAGCCGGAGGAAGGTGAGGATGACGTCAAGTCATCATGCCCCTTATGCCCT
GGGCGACACACGTGCTACAATGGCCGGGACAAAGGGTCGCGATCCCGCGAGGGTGA
GCTAACTCCAAAAACCCGTCCTCAGTTCGGATTGCAGGCTGCAACTCGCCTGCATGA
AGCCGGAATCGCTAGTAATCGCCGGTCAGCCATACGGCGGTGAATTCGTTCCCGGG
CCTTGTACACACCGCCCGTCACACTATGGGAGCTGACCATGCCCGAAGTCGTTACCT
TAACCGCAAGGAGGGGGATGCCGAAGGCAGGGCTAGTGACTGGAGTGAAGTCGTA
ACAAGGTAGCCGTACTGGAAGGTGCGGCTGGATCACCTCCTTT
>P021_WC05_k50.5034 [Aisa terna, matK (partial)]
TCGCACTATGTATCATTTGATAATCCCAAAATCTTCTACCTTTGGTTCAAATCTAATA
TCAAATGGAGGAAATCCAAAGATATTTACAGCTTGATAGATCTCAACAACACGGCTT
TCTATATCCACTTATCTTTCAGGAGTATATTTATGCACTTGCTCATGATCATAGTTTA
AACCGA

>P015_WD05_k90.2560 [Anzygina zealandica, 16S (partial)]
TCTCATGGAGAGTTCGATCCTGGCTCAGGATGAACGCTGGCGGCAGGCCTTACACAT
GCAAGTCGGACGGGAAGTGGTGTTTCCAGTGGCGGACGGGTGAGTAACGCGTAAGA
ACCTGCCCTTGGGAGGGGAACAACAGCTGGAAACGGTTGCTAATACCCCGTAGGCT
GAGGAGCAAAAGGAGGAATCCGCCCGAGGAGGGGCTCGCGTCTGATTAGCTAGTTG
GTGAGGCAATAGCTTACCAAGGCGATGATCAGTAGCTGGTCCGAGAGGATGATCAG
CCACACTGGGACTGAGACACGGCCCAGACTCCTACGGGAGGCAGCAGTGGGGAATT
TTCCGCAATGGGCGAAAGCCTGACGGAGCAATGCCGCGTGGAGGTAGAAGGCCTAC
GGGTCGTGAACTTCTTTTCCCGGAGAAGAAGCAATGACGGTATCCGGGGAATAAGC
ATCGGCTAACTCTGTGCCAGCAGCCGCGGTAAGACAGAGGATGCAAGCGTTATCCG
GAATGATTGGGCGTAAAGCGTCTGTAGGTGGCTTTTTAAGTTCGCCGTCAAATCCCA
GGGCTCAACCCTGGACAGGCGGTGGAAACTACCAAGCTGGAGTACGGTAGGGGCAG
AGGGAATTTCCGGTGGAGCGGTGAAATGCGTAGAGATCGGAAAGAACACCAACGGC
GAAAGCACTCTGCTGGGCTGACACTGACACTGAGAGACGAAAGCTAGGGGAGCGAA
TGGGATTAGATACCCCAGTAGTCCTAGCCGTAAACGATGGATACTAGGCGCTGTGCG
TATCGACCCGTGCAATGCTGTAGCTAACGCG
>P019_WB12_k90.938 [Gladkara albida, 16S]
TCTCATGGAGAGTTCGATCCTGGCTCAGGATGAACGCTGGCGGCATGCTTAACACAT
GCAAGTCGGACGGGAAGTGGTGTTTCCAGTGGCGGACGGGTGAGTAACGCGTAAGA
ACCTGCCCTTGGGAGGGGAACAACAGCTGGAAACGGCTGCTAATACCCCGTAGGCT
GAGGAGCAAAAGGAGGAATCCGCCCGAGGAGGGGCTCGCGTCTGATTAGCTAGTTG
GTGAGGCAATAGCTTACCAAGGCGATGATCAGTAGCTGGTCCGAGAGGATGATCAG
CCACACTGGGACTGAGACACGGCCCAGACTCCTACGGGAGGCAGCAGTGGGGAATT
TTCCGCAATGGGCGAAAGCCTGACGGAGCAATGCCGCGTGGAGGTAGAAGGCCTAC
GGGTCGTGAACTTCTTTTCCCGGAGAAGAAGCAATGACGGTATCTGGGGAATAAGC
ATCGGCTAACTCTGTGCCAGCAGCCGCGGTAATACAGAGGATGCAAGCGTTATCCG
GAATGATTGGGCGTAAAGCGTCTGTAGGTGGCTTTTTAAGTCCGCCGTCAAATCCCA
GGGCTCAACCCTGGACAGGCGGTGGAAACTGCCAAGCTGGAGTACGGTAGGGGCAG
AGGGAATTTCCGGTGGAGCGGTGAAATGCGTAGAGATCGGAAAGAACACCAACGGC
GAAAGCACTCTGCTGGGCCGACACTGACACTGAGAGACGAAAGCTAGGGGAGCGA
ATGGGATTAGATACCCCAGTAGTCCTAGCCGTAAACGATGGATACTGGGCGCTGTGC
GTATCGACCCGTGCAGTGCTGTAGCTAACGCGTTAAGTATCCCGCCTGGGGAGTACG
TTCGCAAGAATGAAACTCAAAGGAATTGACGGGGGCCCGCACAAGCGGTGGAGCAT
GTGGTTTAATTCGATGCAAAGCGAAGAACCTTACCAGGGCTTGACATGCCGCGAATC
CTCTTGAAAGAGAGGGGTGCCTTCGGGAACGCGGACACAGGTGGTGCATGGCTGTC
GTCAGCTCGTGCCGTAAGGTGTTGGGTTAAGTCCCGCAACGAGCGCAACCCTCGTGT
TTAGTTGCCACCGTTGAGTTTGGAACCCTGAGCAGACTGCCGGCGATAAGCCGGAG
GAAGGTGAGGATGACGTCAAGTCATCATGCCCCTTATGCCCTGGGCGACACACGTG
CTACAATGGCCGGGACAAAGGGTCGCGATCCCGCGAGGGTGAGCTAACTCCAAAAA
CCCGTCCTCAGTTCGGATTGCAGGCTGCAACTCGCCTGCATGAAGCCGGAATCGCTA
GTAATCGCCGGTCAGCCATACGGCGGTGAATTCGTTCCCGGGCCTTGTACACACCGC
CCGTCACACTATGGGAGCTGACCATGCCCGAAGTCGTTACCTTAACCGCAAGGAGG
GGGATGCCGAAGGCAGGGCTAGTGACTGGAGTGAAGTCGTAACAAGGTAGCCGTAC
TGGAAGGTGCGGCTGGATCACCTCCTTT
>P019_WB12_k50.16171 [Gladkara albida, rbcL (partial)]

AATTGAACAAGTATGGCCGTCCTCTACTGGGATGCACTATTAAGCCGAAATTGGGGT
TATCTGCTAAAAACTATGGTCGAGCGGTTTATGAATGTCTTCGCGGTGGACTTGATT
TTACCAAAGATGACGAAAACGTGAACTCCCAACCATTTATGCGTTGGAGAGACCGTT
TCTTAT
>P015_WD08_k90.3846 [Gredzinskiya lamellaris, 16S (partial)]
TCTCATGGAGAGTTCGATCCTGGCTCAGGATGAACGCTGGCGGCATGCTTAACACAT
GCAAGTCGGACGGGAAGTGGTGTTTCCAGTGGCGGACGGGTGAGTAACGCGTAAGA
ACCTGCCCTTGGGAGGGGAACAACAGCTGGAAACGGCTGCTAATACCCCGTAGGCT
GAGGAGCAAAAGGAGGAATCCGCCCGAGGAGGGGCTCGCGTCTGATTAGCTAGTTG
GTGAGGCAATAGCTTACCAAGGCGATGATCAGTAGCTGGTCCGAGAGGATGATCAG
CCACACTGGGACTGAGACACGGCCCAGACTCCTACGGGAGGCAGCAGTGGGGAATT
TTCCGCAATGGGCGAAAGCCTGACGGAGCAATGCCGCGTGGAGGTAGAAGGCCTAC
GGGTCGTTAACTTCTTTTCCTGGAGAAGAAGTGATGACGGTATCTGGGGAATAAGCA
TCGGCTAACTCTGTGCCAGCAGCCGCGGTAAGACAGAGGATGCAAGCGTTATCCGG
AATGATTGGGCGTAAAGCGTCTGTAGGTGGCTTTTTAAGTTCGCCGTCAAATCCCAG
GGCTCAACCCTGGACAGGCGGTGGAAACTACCAAGCTGGAGTACGGTAGGGGCAGA
GGGAATTTCCGGTGGAGCGGTGAAATGCGTAGAGATCGGAAAGAACACCAACGGCG
AAAGCACTCTGCTGGGCCGACACTGACACTGAGAGACGAAAGCTAGGGGAGCGAAT
GGGATTAGATACCCCAGTAGTCCTAGCCGTAAACGATGGATACTAGGCGCTGTGCGT
ATCGACCCGTGCAGTGCTGTAGCTAACGCGTTAAGTATCCCGCCTGGGGAGTACGTT
CGCAAGAATGAAACTCAAAGGAATTGACGGGGGCCCGCACAAGCGGTGGAGCATGT
GGTTTAATTCGATGCAAAGCGAAGAACCTTACCAGGG
>P015_WD09_k50.15617 [Matsumurina kagina, 16S (partial)]
TCTCATGGAGAGTTCGATCCTGGCTCAGGATGAACGCTGGCGGCATGCTTAACACAT
GCAAGTCGGACGGGAAGTGGTGTTTCCAGTGGCGGACGGGTGAGTAACGCGTAAGA
ACCTGCCCTTGGGAGGGGAACAACAGCTGGAAACGGCTGCTAATACCCCGTAGGCT
GAGGAGCAAAAGGAGGAATCCGCCCGAGGAGGGGCTCGCGTCTGATTAGCTAGTTG
GTGAGGCAATAGCTTACCAAGGCGATGATCAGTAGCTGGTCCGAGAGGATGATCAG
CCACACTGGGACTGAGACACGGCCCAGACTCCTACGGGAGGCAGCAGTGGGGAATT
TTCCGCAATGGGCGAAAGCCTGACGGAGCAATGCCGCGTGGAGGTAGAAGGCCCAC
GGGTCGTGAACTTCTTTTCCCGGAGAAGAAGCAATGACGGTATCTGGGGAATAAGC
ATCGGCTAACTCTGTGCCAGCAGCCGCGGTAATACAGAGGATGCAAGCGTTATCCG
GAATGATTGGGCGTAAAGCGTCTGTAGGTGGCTTTTTAAGTCCGCCGTCAAATCCCA
GGGCTCAACCCTGGACAGGCGGTGGAAACTACCAAGCTGGAGTACGGTAGGGGCAG
AGGGAATTTCCGGTGGAGCGGTGAAATGCGTAGAGATCGGAAAGAACACCAACGGC
GAAAGCACTCTGCTGGGCCGACACTGACACTGAGAGACGAAAGCTAGGGGAGCGA
ATGGGATTAGATACCCCAGTAGTCCTAGCCGTAAACGATGGATACTAGGCGCTGTGC
GTATCGACCCGTGCAGTGCTGTAGCTAACGCGTTAAGTATCCCGCCTGGGGAGTACG
TTCGCAAGAATGAAACTCAAAGGAATTGACGGGGGCCCGCACAAGCGGTGGAGCAT
GTGGTTTAATTCGATGCAAAGCGAAGAACCTTACCAGGGCTTGACATGCCGCGAATC
CTCTTGAAAGAGAGGGGTGCCTTCGGGAACGCGGACACAGGTGGTGCATGGCTGTC
GTCAGCTCGTGCCGTAAGGTGTTGGGTTAAGTCCCGCAACGAGCGCAACCCTCGTGC
TTAGTTGCCACCGTTGAGTTTGGAACCCTGAGCAGACTGCCGGTGATAAGCCGGAGG
AAGGTGAGGATGACGTCAAGTCATCATGCCCCTTATGCCCTGGGCGACACACGTGCT
ACAATGGCCGGGACAAAGGGTCGCGATCCCGCGAGGGTGAGCTAACTCCAAAAACC

CGTCCTCAGTTCGGATTGCAGGCTGCAACTCGCCTGCATGAAGCCGGAATCGCTAGT
AATCGCCGGTCAGCCATACGGCGGTGAATTCGTTCCCGGGCCTTGTACACACCGCCC
>P019_WH01_k90.1902 [Mitjaevia amseli, 16S (partial)]
AGCGGTGAAATGCGTAGAGATCGGAAAGAACACCAACGGCGAAAGCACTCTGCTGG
GCCGACACTGACACTGAGAGACGAAAGCTAGGGGAGCAAATGGGATTAGATACCCC
AGTAGTCCTAGCCGTAAACGATGGATACTAGGCGCTGTGCGTATCGACCCGTGCAGT
GCTGTAGCTAACGCGTTAAGTATCCCGCCTGGGGAGTACGTTCGCAAGAATGAAACT
CAAAGGAATTGACGGGGGCCCGCACAAGCGGTGGAGCATGTGGTTTAATTCGATGC
AAAGCGAAGAACCTTACCAGGGCTTGACATGCCGTGAATCCTCTTGAAAGAGAGGG
GTGCCTTCGGGAACGCGGACACAGGTGGTGCATGGCTGTCGTCAGCTCGTGCCGTAA
GGTGTTGGGTTAAGTCCCGCAACGAGCGCAACCCTCGTGTTTAGTTGCCACCATTGA
GTTTGGAACCCTGAACAGACTGCCGGTGATAAGCCGGAGGAAGGTGGGGATGACGT
CAAGTCATCATGCCCCTTATGCCCTGGGCGACACACGTGCTACAATGGCCGGGACA
AAGGGTCGCGATCCCGCGAGGGTGAGCTAACCCCAAAAACCCGTCCTCAGTTCGGA
TTGCAGGCTGCAACTCGCCTGCATGAAGCCGGAATCGCTAGTAATCGCCGGTCAGCC
ATACGGCGGTGAATTCGTTCCCGGGCCTTGTACACACCGCCCGTCACACTATGGGAG
CTGGCCATGCCCGAAGTCGTTACCTTAACCGCAAGGAGGGGGATGCCGAAGGCGGG
GCTAGTGACTGGAGTGAAGTCGTAACAAGGTAGCCGTACTGGAAGGTGCGGCTGGA
TCACCTCCTTT
>P019_WH01_k50.1932 [Mitjaevia amseli, rbcL (partial)]
GCCCTGCGTGCTCTACGTTTGGAGGATTTGCGAATCCCTGTTGCTTATATAAAAACTT
TCCAAGGCCCGCCTCACGGTATCCAAGTTGAGAGAGATAAATTGAACAAGTATGGC
CGTCCTCTACTGGGATGCACTATTAAGCCGAAATTGGGGTTATCTGCTAAAAACTAT
GGTCGAGCGGTTTATGAATGTCTTCGCGGTGGACTTGATTTTACC
>P019_WH01_k29.10253 [Mitjaevia amseli, rbcL (partial)]
TTGACTTATTATACTCCTGAATATCAACCCCAGGATACCGATATCTTGGCAGCATTCC
GAGTAACTCCTCAACCAGGAGTTCCGTCAGAAGAAGCAGGAGCCGCAGTAGCTGCC
GAATCTTCTACTGGTACATGGACAACTGTATGGACCGACGGACTTACCAGTCTTGAT
CGTTACAAAGGACGATGCTACCACATCGATGCCGTTCCTGGAGAAGACAATCAATA
TATTTGTTATGTAGCTTACCCCTTAGACCTTTTTG
>P021_WG02_k90.1788 [Molopopterus n. sp. 1, matK (partial)]
CTATATCGCACTATGTATCATTTGATAATCCCAAAATCTTCTACCTTTGGTTCAAATC
TAATATCAAATGGAGGAAATCCAAAGATATTTACAGCTTGATAGATCTCAACAACA
CGGCTTTCTATATCCACTTATCTTTCAGGAGTATATTTATGCACTTGCTCATGATCAT
AGTTTAAACCGATCTATTTTGTTGGAAAATCCAGGTCATGACAATCAATTCAGTTTC
CTAATTGTGAAACGTTTA
>P010_WG01_k50.12538 [Musbrnoia corollaris, 16S (partial)]
CTGGACAGGCGGTGGAAACTACCAAGCTGGAGTACGGTAGGGGCAGAGGGAATTTC
CGGTGGAGCGGTGAAATGCGTAGAGATCGGAAAGAACACCAACGGCGAAAGCACT
CTGCTGGGCCGACACTGACACTGAGAGACGAAAGCTAGGGGAGCGAATGGGATTAG
ATACCCCAGTAGTCCTAGCCGTAAACGATGGATACTAGGCGCTGTGCGTATCGACCC
GTGCAGTGCTGTAGCTAACGCGTTAAGTATCCCGCCTGGGGAGTACGTTCGCAAGAA
TGAAACTCAAAGGAATTGACGGGGGCCCGCACAAGCGGTGGAGCATGTGGTTTAAT
TCGATGCAAAGCGAAGAACCTTACCAGGGATTGACATGCCGCGAATCCTCTTGAAA
GAGAGGGGTGCCTTCGGGAACGCGGACACAGGTGGTGCATGGCTGTCGTCAGCTCG
TGCCGTAAGGTGTTGGGTTAAGTCCCGCAACGAGCGCAACCCTCGTGTTTAGTTGCC

ACCATTAAGTTTGGAACCCTGAGCAGACTGCCGGTGATAAGCCGGAGGAAGGTGAG
GATGACGTCAAGTCATCATGCCCCTTATGCCCTGGGCGACACACGTGCTACAATGGC
CGGGACAAAGGGTCGCGATCCCGCGAGGGTGAGCTAACTCCAAAAACCCGTCCTCA
GTTCGGATTGCAGGCTGCAACTCGCCTGCATGAAGCCGGAATCGCTAGTAATCGCCG
GTCAGCCATACGGCGGTGAATTCGTTCCCGGGCCTTGTACACACCGCCCGTCACACT
ATGGGAGCTGGCCA
>P010_WB12_k90.7302 [Neozygina penapacha, 16S (partial)]
AGGCGGTGGAAACTACCAAGCTGGAGTACGGTAGGGGCAGAGGGAATTTCCGGTGG
AGCGGTGAAATGCGTAGAGATCGGAAAGAACACCAACGGCGAAAGCACTCTGCTGG
GCCGACACTGACACTGAGAGACGAAAGCTAGGGGAGCGAATGGGATTAGATACCCC
AGTAGTCCTAGCCGTAAACGATGGATACTAGGCGCTGTGCGTATCGACCCGTGCAGT
GCTGTAGCTAACGCGTTAAGTATCCCGCCTGGGGAGTACGTTCGCAAGAATGAAACT
CAAAGGAATTGACGGGGGCCCGCACAAGCGGTGGAGCATGTGGTTTAATTCGATGC
AAAGCGAAGAACCTTACCAGGGCTTGACATGCCGCGAATCCTCTTGAAAGAGAGGG
GTGCCTTCGGGAACGCGGACACAGGTGGTGCATGGCTGTCGTCAGCTCGTGCCGTAA
GGTGTTGGGTTAAGTCCCGCAACGAGCGCAACCCTCGTGTTTAGTTGCCAACGTTGA
GTTTGGAACCCTGAGCAGACTGCCGGTGATAAGCCGGAGGAAGGTGAGGATGACGT
CAAGTCATCATGCCCCTTATGCCCTGGGCGACACACGTGCTACAATGGCCGGGACA
AAGGATCGCGATCCCGCGAGGGTGAGCTAACTCCAAAAACCCGTCCTCAGTTCGGA
TTGTAGGCTGCAACTCGCCTGCATGAAGCCGGAATCGCTAGTAATCGCCGGTCAGCC
ATACGGCGGTGAATTCGTTCCCGGGCCTTGTACACACCGCCCGTCACACTATGGGAG
CTGGCCATGCCCGAAGTCGTTACCTTAACCGCAAGGAGGGGGATGCCGAAGGCAGG
GCTAGTGACTGGAGTGAAGTCGTAACAAGGTAGCCGTACTGGAAGGTGCGGCTGGA
TCACCTCCTTT
>P010_WC01_k90.6234 [Neozygina zapatai, 16S (partial)]
AAACTACCAAGCTGGAGTACGGTAGGGGCAGAGGGAATTTCCGGTGGAGCGGTGAA
ATGCGTAGAGATCGGAAAGAACACCAACGGCGAAAGCACTCTGCTGGGCCGACACT
GACACTGAGAGACGAAAGCTAGGGGAGCGAATGGGATTAGATACCCCAGTAGTCCT
AGCCGTAAACGATGGATACTAGGCGCTGTGCGTATCGACCCGTGCAGTGCTGTAGCT
AACGCGTTAAGTATCCCGCCTGGGGAGTACGTTCGCAAGAATGAAACTCAAAGGAA
TTGACGGGGGCCCGCACAAGCGGTGGAGCATGTGGTTTAATTCGATGCAAAGCGAA
GAACCTTACCAGGGCTTGACATGCCGCGAATCCTCTTGAAAGAGAGGGGTGCCTTCG
GGAACGCGGACACAGGTGGTGCATGGCTGTCGTCAGCTCGTGCCGTAAGGTGTTGG
GTTAAGTCCCGCAACGAGCGCAACCCTCGTGTTTAGTTGCCAACGTTGAGTTTGGAA
CCCTGAGCAGACTGCCGGTGATAAGCCGGAGGAAGGTGAGGATGACGTCAAGTCAT
CATGCCCCTTATGCCCTGGGCGACACACGTGCTACAATGGCCGGGACAAAGGATCG
CGATCCCGCGAGGGTGAGCTAACTCCAAAAACCCGTCCTCAGTTCGGATTGTAGGCT
GCAACTCGCCTGCATGAAGCCGGAATCGCTAGTAATCGCCGGTCAGCCATACGGCG
GTGAATTCGTTCCCGGGCCTTGTACACACCGCCCGTCACACTATGGGAGCTGGCCAT
GCCCGAAGTCGTTACCTTAACCGCAAGGAGGGGGATGCCGAAGGCAGGGCTAGTGA
CTGGAGTGAAGTCGTAACAAG
>P010_WG03_k90.3253 [Nkumba omani, 16S (partial)]
TCTCATGGAGAGTTCGATCCTGGCTCAGGATGAACGCTGGCGGCATGCTTAACACAT
GCAAGTCGGACGGGAAGTGGTGTTTCCAGTGGCGGACGGGTGAGTAACGCGTAAGA
ACCTGCCCTTGGGAGGGGAACAACAGCTGGAAACGGCTGCTAATACCCCGTAGGCT
GAGGAGCAAAAGGAGGAATCCGCCCGAGGAGGGGCTCGCGTCTGATTAGCTAGTTG

GTGAGGCAATAGCTTACCAAGGCGATGATCAGTAGCTGGTCCGAGAGGATGATCAG
CCACACTGGGACTGAGACACGGCCCAGACTCCTACGGGAGGCAGCAGTGGGGAATT
TTCCGCAATGGGCGAAAGCCTGACGGAGCAATGCCGCGTGGAGGTAGAAGGCCTAC
GGGTCGTTAACTTCTTTTCCCGGAGAAGAAGTGATGACGGTATCTGGGGAATAAGCA
TCGGCTAACTCTGTGCCAGCAGCCGCGGTAAGACAGAGGATGCAAGCGTTATCCGG
AATGATTGGGCGTAAAGCGTCTGTAGGTGGCTTTTTAAGTTCGCCGTCAAATCCCAG
GGCTCAACCCTGGACAGGCGGTGGAAACTACCAAGCTGGAGTACGGTAGGGGCAGA
GGGAATTTCCGGTGGAGCGGTGAAATGCGTAGAGATCGGAAAGAACACCAACGGCG
AAAGCACTCTGCTGGGCCGACACTGACACTGAGAGACGAAAGCTAGGGGAGCGAAT
GGGATTAGATACCCCAGTAGTCCTAGCCGTAAACGATGGATACTAGGCGCTGTGCGT
ATCGACCCGTGCAGTGCTGTAGCTAACGCGTTAAGTATCCCGCC
>P010_WG04_k90.90 [Nsesa cameroonica, 16S]
TCTCATGGAGAGTTCGATCCTGGCTCAGGATGAACGCTGGCGGCATGCTTAACACAT
GCAAGTCGGACGGGAAGTGGTGTTTCCAGTGGCGGACGGGTGAGTAACGCGTAAGA
ACCTGCCCTTGGGAGGGGAACAACAGCTGGAAACGGCTGCTAATACCCCGTAGGCT
GAGGAGTAAAAGGAGGAATCCGCCCGAGGAGGGGCTCGCGTCTGATTAGCTAGTTG
GTGAGGCAATAGCTTACCAAGGCGATGATCAGTAGCTGGTCCGAGAGGATGATCAG
CCACACTGGGACTGAGACACGGCCCAGACTCCTACGGGAGGCAGCAGTGGGGAATT
TTCCGCAATGGGCGAAAGCCTGACGGAGCAATGCCGCGTGGAGGTAGAAGGCCTAC
GGGTCGTGAACTTCTTTTCCCGGAGAAGAAGCAATGACGGTATCTGGGGAATAAGC
ATCGGCTAACTCTGTGCCAGCAGCCGCGGTAATACAGAGGATGCAAGCGTTATCCG
GAATGATTGGGCGTAAAGCGTCTGTAGGTGGCTTTTTAAGTCCGCCGTCAAATCCCA
GGGCTCAACCCTGGACAGGCGGTGGAAACTGCCAAGCTGGAGTACGGTAGGGGCAG
AGGGAATTTCCGGTGGAGCGGTGAAATGCGTAGAGATCGGAAAGAACACCAACGGC
GAAAGCACTCTGCTGGGCCGACACTGACACTGAGAGACGAAAGCTAGGGGAGCGA
ATGGGATTAGATACCCCAGTAGTCCTAGCCGTAAACGATGGATACTGGGCGCTGTGC
GTATCGACCCGTGCAGTGCTGTAGCTAACGCGTTAAGTATCCCGCCTGGGGAGTACG
TTCGCAAGAATGAAACTCAAAGGAATTGACGGGGGCCCGCACAAGCGGTGGAGCAT
GTGGTTTAATTCGATGCAAAGCGAAGAACCTTACCAGGGCTTGACATGCCGCGAATC
CTCTTGAAAGGGAGGGGTGCCTTCGGGAACGCGGACACAGGTGGTGCATGGCTGTC
GTCAGCTCGTGCCGTAAGGTGTTGGGTTAAGTCCCGCAACGAGCGCAACCCTCGTGT
TTAGTTGCCACCGTTGAGTTTGGAACCCTGAGCAGACTGCCGGTGATAAGCCGGAGG
AAGGTGAGGATGACGTCAAGTCATCATGCCCCTTATGCCCTGGGCGACACACGTGCT
ACAATGGCCGGGACAAAGGGTCGCGATCCCGCGAGGGTGAGCTAACTCCAAAAACC
CGTCCTCAGTTCGGATTGCAGGCTGCAACTCGCCTGCATGAAGCCGGAATCGCTAGT
AATCGCCGGTCAGCCATACGGCGGTGAATTCGTTCCCGGGCCTTGTACACACCGCCC
GTCACACTATGGGAGCTGACCATGCCCGAAGTCGTTACCTTAACCGTAAGGAGGGG
GATGCCGAAGGCAGGGCTAGTGACTGGAGTGAAGTCGTAACAAGGTAGCCGTACTG
GAAGGTGCGGCTGGATCACCTCCTTT
>P019_WB11_k29.57859 [Seriana ochrata, rbcL (partial)]
CACGGTATCCAAGTTGAGAGAGATAAATTGAACAAGTATGGCCGTCCTCTACTGGG
ATGCACTATTAAGCCGAAATTGGGGTTATCTGCTAAAAACTATGGTCGAGCGGTTTA
TGAATGTCTTCGCGGTGGACTTGATTTACCAAAGATGACGAAAACGTGAACTCCCA
ACCATTTATGCGTTGGAGAGACCGTTTCTTATTTGTGCCGAAGCAATTTATAAAGC
ACAGGCCGAAACAGGTGAAATCAAAGGACATTACTTGAATGCTACCGCAGGTACAT

GCGAAGAAATGATAAAAAGGGCTGTATTTGCCAGAGAATTGGGTGTTCCTATCATA
ATGCATGACTACTTAACAGGTGGATTCACTGCAAATACTAGCTTGGCTCTTTATT
>P019_WB08_k90.1632 [Szymczakowskia linnavuorii, 16S (partial)]
TCTCATGGAGAGTTCGATCCTGGCTCAGGATGAACGCTGGCGGCATGCTTAACACAT
GCAAGTCGGACGGGAAGTGGTGTTTCCAGTGGCGGACGGGTGAGTAACGCGTAAGA
ACCTGCCCTTGGGAGGGGAACAACAGCTGGAAACGGCTGCTAATACCCCGTAGGCT
GAGGAGCAAAAGGAGGAATCCGCCCGAGGAGGGGCTCGCGTCTGATTAGCTAGTTG
GTGAGGCAATAGCTTACCAAGGCGATGATCAGTAGCTGGTCCGAGAGGATGATCAG
CCACACTGGGACTGAGACACGGCCCAGACTCCTACGGGAGGCAGCAGTGGGGAATT
TTCCGCAATGGGCGAAAGCCTGACGGAGCAATGCCGCGTGGAGGTAGAAGGCCTAC
GGGTCGTTAACTTCTTTTCCCGGAGAAGAAGTAATGACGGTATCTGGGGAATAAGCA
TCGGCTAACTCTGTGCCAGCAGCCGCGGTAAGACAGAGGATGCAAGCGTTATCCGG
AATGATTGGGCGTAAAGCGTCTGTAGGTGGCTTTTTAAGTTCGCCGTCAAATCCCAG
GGCTCAACCCTGGACAGGCGGTGGAAACTACCAAGCTGGAGTACGGTAGGGGCAGA
GGGAATTTCCGGTGGAGCGGTGAAATGCGTAGAGATCGGAAAGAACACCAACGGCG
AAAGCACTCTGCTGGGCCGACACTGACACTGAGAGACGAAAGCTAGGGGAGCGAAT
GGGATTAGATACCCCAGTAGTCCTAGCCGTAAACGATGGATACTAGGCGCTGTGCGT
ATCGACCCGTGCAGTGCTGTAGCTAACGCGTTAAGTATCCCGCCTGGGGAGTACGTT
CGCAAGAATGAAACTCAAAGGAATTGACGGGGGCCCGCACAAGCGGTGGAGCATGT
GGTTTAATTCGATGCAAAGCGAAGAACCTTACCAGG
>P019_WC03_k50.28525 [Thaia barbata, matK (partial)]
TGTATCATTTGATAATCCCAAAATCTTCTACCTTTGGTTCAAATCTAATATCAAATGG
AGGAAATCCAAAGATATTTACAGCTTGATAGATCTCAACAACACGGCTTTCTATATC
CACTTATCTTTCAGGAGTATATTTATGCACTTGCTCATGATCATAGTTTAAACCGATC
TATTTTGTTGGAAAATCCAGGTTATGACAATCAATTCAGTTTCCTAATTGTGAAACGT
TTAATTAC
>P014_WH08_k90.8281 [Variolosa meni, 16S]
TCTCATGGAGAGTTCGATCCCGGCTCAGGATGAACGCTGGCGGCATGCCTAACACAT
GCAAGTCGGACGGGAAGTGGTGTTTCCAGTGGCGGACGGGTGAGTAACGCGTAAGA
ACCTGCCCTTGGGAGGGGAACAACAACTGGAAACGGCTGCTAATACCCCGTAGGCT
GAGGAGCAAAAGGAGGAATCTGCCCGAGGAGGGGCTCGCGTCTGATTAGCTAGTTG
GTGAGGCAATAGCTTACCAAGGCGATGATCAGTAGCTGGTCCGAGAGGATGATCAG
CCACACTGGGACTGAGACACGGCCCAGACTCCTACGGGAGGCAGCAGTGGGGAATT
TTCCGCAATGGGCGAAAGCCTGACGGAGCAATGCCGCGTGGAGGTAGAAGGCCCAC
GGGTCATGAACTTCTTTTCCCGGAGAAGAAGCAATGACGGTATCTGAGGAATAAGC
ATCGGCTAACTCTGTGCCAGCAGCCGCGGTAAGACAGAGGATGCAAGCGTTATCTG
GAATGATTGGGCGTAAGGCGTCTGTAGGTGGCTTTTCAAGTCCGCCGTCCAATCCCA
GGGCTCAACCCTGGACAGGCGGTGGAAACTACCAAGCTGGAGTACGGTAGGGGCAG
AGGGAATTTCCGGTGGAGCGGTGAAATGCGTAGAGATCGGAAAGAACACCAACGGC
GAAAGCACCCTGCTGGGCCGACACTGACACTGAGAGACGAAAGCTAGGGGAGCGA
ATGGGATTAGATACCCCAGTAGTCCTAGCCGTAAACGATGGATACTAGGCGCTGTGC
GTATCGACCCGTGCAGTGCTGTAGCTAACGCGTTAAGTATCCCGCCTGGGGAGTACG
TTCGCAAGAATGAAACTCAAAGGAATTGACGGGGGCCCGCACAAGCGGTGGAGCAT
GTGGTTTAATTCGATGCAAAGCGAAGAACCTTACCAGGGCTTGACATGCCGTGAATC
CTCTTGAAAGAGAGGGGTGCCTTCGGGAACGCGGACACAGGTGGTGCATGGCTGTC
GTCAGCTCGTGCCGTAAGGTGTTGGGTTAAGTCCCGCAACGAGCGCAACCCTCGTGT

TTAGTTGCCACCATTGAGTTTGGAACCCTGAACAGACCGCCGGTGATAAGCCGGAG
GAAGGTGAGGATGACGTCAAGTCATCATGCCCCCTATGCCCTGGGCGACACACGTG
TTACAATGGCCGGGACAAAGGGTCGCGATCCCGCCAGGGTGAGCTAACTCCAAAAA
CCCGTCCTAAGTTCGGATTGCAGGCTGCAACTCGCCTGCATGAAGCCGGAATCGCTA
GTAATCGCCGGTCAGCCATACGGCGGTGAATTCGTTCCCGGGCCTTGTACACACCGC
CCGTCACACTGTGGGAGCTGGCTATGCCCGAAGTCGTTACCTTAACCGCAAGGAGG
GGGATGCCGAAGGCGGGGCTAGTGACTGGAGTGAAGTCGTAACAAGGTAGCCGTAC
TGGAAGGTGCGGCTGGATCACCTCCTTT
>P010_WB11_k90.2115 [Zyginama n. sp. 2, 16S (partial)]
CGAGAGGATGATCAGCCACACTGGGACTGAGACACGGCCCAGACTCCTACGGGAGG
CAGCAGTGGGGAATTTTCCGCAATGGGCGAAAGCCTGACGGAGCAATGCCGCGTGG
AGGTAGAAGGCCTACGGGTCGTGAACTTCTTTTCCCGGAGAAGAAGCAATGACGGT
ATCTGGGGAATAAGCATCGGCTAACTCTGTGCCAGCAGCCGCGGTAATACAGAGGA
TGCAAGCGTTATCCGGAATGATTGGGCGTAAAGCGTCTGTAGGTGGCTTTTTAAGTC
CGCCGTCAAATCCCAGGGCTCAACCCTGGACAGGCGGTGGAAACTACCAAGCTGGA
GTACGGTAGGGGCAGAGGGAATTTCCGGTGGAGCGGTGAAATGCGTAGAGATCGGA
AAGAACACCAACGGCGAAAGCACTCTGCTGGGCCGACACTGACACTGAGAGACGA
AAGCTAGGGGAGCGAATGGGATTAGATACCCCAGTAGTCCTAGCCGTAAACGATGG
ATACTAGGCGCTGTGCGTATCGACCCGTGCAGTGCTGTAGCTAACGCGTTAAGTATC
CCGCCTGGGGAGTACGTTCGCAAGAATGAAACTCAAAGGAATTGACGGGGGCCCGC
ACAAGCGGTGGAGCATGTGGTTTAATTCGATGCAAAGCGAAGAACCTTACCAGGGC
TTGACATGCCGCGAATCCTCTTGAAAGAGAGGGGTGCCTTCGGGAACGCGGACACA
GGTGGTGCATGGCTGTCGTCAGCTCGTGCCGTAAGGTGTTGGGTTAAGTCCCGCAAC
GAGCGCAACCCTCGTGTTTAGTTGCCACCGTTGAGTTTGGAACCCTGAGCAGACTGC
CGGTGATAAGCCGGAGGAAGGTGAGGATGACGTCAAGTCATCATGCCCCTTATGCC
CTGGGCGACACACGTGCTACAATGGCCGGGACAAAGGGTCGCGATCCCGCGAGGGT
GAGCTAACCCCAAAAACCCGTCCTCAGTTCGGATTGTAGGCTGCAACTCGCCTGCAT
GAAGCCGGAATCGCTAGTAATCGCCGGTCAGCCATACGGCGGTGAATTCGTTCCCG
GGCCTTGTACACACCGCCCGTCACACTATGGGAGCTGGCCATGCCCGAAGTCGTTAC
CTTAACCACAAGGAGGGGGATGCCGAAGGCAGGGCTAGTGACTGGAGTGAAGTCGT
AACAAGGTAGCCGTACTGGAAGGTGCGGCTGGATCACCTCCTTT
>P010_WC11_k90.1686 [Typhlocybini New Genus T2 n. sp. 1, 16S]
TCTCATGGAGAGTTCGATCCTGGCTCAGGATGAACGCTGGCGGCATGCTTAACACAT
GCAAGTCGGACGGGAAGTGGTGTTTCCAGTGGCGGACGGGTGAGTAACGCGTAAGA
ACCTGCCCTTGGGAGGGGAACAACAGCTGGAAACGGCTGCTAATACCCCGTAGGCT
GAGGAGCAAAAGGAGGAATCCGCCCGAGGAGGGGCTCGCGTCTGATTAGCTAGTTG
GTGAGGCAATAGCTTACCAAGGCGATGATCAGTAGCTGGTCCGAGAGGATGATCAG
CCACACTGGGACTGAGACACGGCCCAGACTCCTACGGGAGGCAGCAGTGGGGAATT
TTCCGCAATGGGCGAAAGCCTGACGGAGCAATGCCGCGTGGAGGTAGAAGGCCTAC
GGGTCGTGAACTTCTTTTCCCGGAGAAGAAGCAATGACGGTATCTGGGGAATAAGC
ATCGGCTAACTCTGTGCCAGCAGCCGCGGTAATACAGAGGATGCAAGCGTTATCCG
GAATGATTGGGCGTAAAGCGTCTGTAGGTGGCTTTTTAAGTCCGCCGTCAAATCCCA
GGGCTCAACCCTGGACAGGCGGTGGAAACTACCAAGCTGGAGTACGGTAGGGGCAG
AGGGAATTTCCGGTGGAGCGGTGAAATGCGTAGAGATCGGAAAGAACACCAACGGC
GAAAGCACTCTGCTGGGCCGACACTGACACTGAGAGACGAAAGCTAGGGGAGCAA
ATGGGATTAGATACCCCAGTAGTCCTAGCCGTAAACGATGGATACTAGGCGCTGTGC

GTATCGACCCGTGCAGTGCTGTAGCTAACGCGTTAAGTATCCCGCCTGGGGAGTACG
TTCGCAAGAATGAAACTCAAAGGAATTGACGGGGGCCCGCACAAGCGGTGGAGCAT
GTGGTTTAATTCGATGCAAAGCGAAGAACCTTACCAGGGCTTGACATGCCGCGAATC
CTCTTGAAAGAGAGGGGTGCCTTCGGGAACGCGGACACAGGTGGTGCATGGCTGTC
GTCAGCTCGTGCCGTAAGGTGTTGGGTTAAGTCCCGCAACGAGCGCAACCCTCGTGT
TTAGTTGCCACCGTTGAGTTTGGAACCCTGAGCAGACTGCCGGTGATAAGCCGGAGG
AAGGTGAGGATGACGTCAAGTCATCATGCCCCTTATGCCCTGGGCGACACACGTGCT
ACAATGGCCGGGACAAAGGGTCGCGATCCCGCGAGGGTGAGCTAACTCCAAAAACC
CGTCCTCAGTTCGGATTGTAGGCTGCAACTCGCCTACATGAAGCCGGAATCGCTAGT
AATCGCCGGTCAGCCATACGGCGGTGAATTCGTTCCCGGGCCTTGTACACACCGCCC
GTCACACTATGGGAGCTGGCCATGCCCGAAGTCGTTACCTTAACCGCAAGGGGGGG
GATGCCGAAGGCAGGGCTAGTGACTGGAGTGAAGTCGTAACAAGGTAGCCGTACTG
GAAGGTGCGGCTGGATCACCTCCTTT
>P021_WG12_k90.19 [Zyginella pulchra, 16S (partial)]
GAACACCAACGGCGAAAGCACTCTGCTGGGCCGACACTGACACTGAGAGACGAAA
GCTAGGGGAGCGAATGGGATTAGATACCCCAGTAGTCCTAGCCGTAAACGATGGAT
ACTAGGCGCTGTGCGTATCGACCCGTGCAGTGCTGTAGCTAACGCGTTAAGTATCCC
GCCTGGGGAGTACGTTCGCAAGAATGAAACTCAAAGGAATTGACGGGGGCCCGCAC
AAGCGGTGGAGCATGTGGTTTAATTCGATGCAAAGCGAAGAACCTTACCAGGGCTT
GACATGCCGCGAATCCTCTTGAAAGAGAGGAGTGCCTTCGGGAACGCGGACACAGG
TGGTGCATGGCTGTCGTCAGCTCGTGCCGTAAGGTGTTGGGTTAAGTCCCGCAACGA
GCGCAACCCTCGTGTTTAGTTGCCACCGTTGAGTTTGGAACCCTGAACAGACTGCCG
GTGATAAGCCGGAGGAAGGTGAGGATGACGTCAAGTCATCATGCCCCTCATGCCCT
GGGCGACACACGTGCTACAATGGCCGGGACAAAGGGTCGCGATCCCGCGAGGGTGA
GCTAACTCCAAAAACCCGTCCTCAGTTCGGATTGCAGGCTGCAACTCGCCTGCATGA
AGCCGGAATCGCTAGTAATCGCCGGTCAGCCATACGGCGGTGAATTCGTTCCCGGG
CCTTGTACACACCGCCCGTCACACTATGGGAGCTGGCCATGCCCGAAGTCGTTACCT
TAACCGCGAGGAGGGGGATGCCGAAGGCAGGGCTAGTGACTGGAGTGAAGTCGTA
ACAAGGTAGCCGTACTGGAAGGTGCGGCTGGATCACCTCCTTT
>P015_WC12_k90.2297 [Tataka boulardi, 16S (partial)]
TGAAATGCGTAGAGATCGGAAAGAACACCAACGGCGAAAGCACTCTGCTGGGCCGA
CACTGACACTGAGAGACGAAAGCTAGGGGAGCGAATGGGATTAGATACCCCAGTAG
TCCTAGCCGTAAACGATGGATACTAGGCGCTGTGCGTATCGACCCGTGCAGTGCTGT
AGCTAACGCGTTAAGTATCCCGCCTGGGGAGTACGTTCGCAAGAATGAAACTCAAA
GGAATTGACGGGGGCCCGCACAAGCGGTGGAGCATGTGGTTTAATTCGATGCAAAG
CGAAGAACCTTACCAGGGCTTGACATGCCGCGAATCCTCTTGAAAGAGAGGGGTGC
CTTCGGGAACGCGGACACAGGTGGTGCATGGCTGTCGTCAGCTCGTGCCGTAAGGT
GTTGGGTTAAGTCCCGCAACGAGCGCAACCCTCGTGTTTAGTTGCCACTGTTGAGTT
TGGAACCCTGAGCAGACTGCCGGTGATAAGCCGGAGGAAGGTGAGGATGACGTCAA
GTCATCATGCCCCTTATGCCCTGGGCGACACACGTGCTACAATGGCCGGGACAAAG
GGTCGCGATCCCGCGAGGGTGAGCTAACTCCAAAAACCCGTCCTCAGTTCGGATTGT
AGGCTGCAACTCGCCTACATGAAGCCGGAATCGCTAGTAATCGCCGGTCAGCCATA
CGGCGGTGAATTCGTTCCCGGGCCTTGTACACACCGCCCGTCACACTATGGGAGCTG
GCCATGCCCGAAGTCGTTACCTTAACCGCAAGGAGGGGGATGCCGAAGGCAGGGCT
AGTGACTGGAGTGAAGTCGTAACAAGGTAGCCGTACTGGAAGGTGCGGCTGGATC
